# Efficient Organocatalytic Dehydrogenation of Ammonia Borane

**DOI:** 10.1002/anie.201910636

**Published:** 2019-12-10

**Authors:** Max Hasenbeck, Jonathan Becker, Urs Gellrich

**Affiliations:** ^1^ Institut für Organische Chemie Justus-Liebig-Universität Gießen Heinrich-Buff-Ring 17 35392 Gießen Germany; ^2^ Institut für Anorganische und Analytische Chemie Justus-Liebig-Universität Gießen Heinrich-Buff-Ring 17 35392 Gießen Germany

**Keywords:** ammonia borane, coupled-cluster computations, hydrogen storage, inorganic retro-ene reactions, organocatalysis

## Abstract

Dehydrogenation of ammonia borane by sterically encumbered pyridones as organocatalysts is reported. With 6‐*tert*‐butyl‐2‐thiopyridone as the catalyst, a turnover frequency (TOF) of 88 h^−1^ was achieved. Experimental mechanistic investigations, substantiated by DLPNO‐CCSD(T) computations, indicate a mechanistic scenario that commences with the protonation of a B−H bond by the mercaptopyridine form of the catalyst. The reactive intermediate formed by this initial protonation was observed by NMR spectroscopy and the molecular structure of a surrogate determined by SCXRD. An intramolecular proton transfer in this intermediate from the NH_3_ group to the pyridine ring with concomitant breaking of the S−B bond regenerates the thiopyridone and closes the catalytic cycle. This step can be described as an inorganic retro‐ene reaction.

## Graphical abstract

**H_2_ go**: Efficient dehydrogenation of ammonia borane by the organic catalyst 6‐*tert*‐butyl‐2‐thiopyridone is described. Mechanistic investigations show that the reaction commences with dehydrogenative S−B coupling. An inorganic retro‐ene reaction closes the catalytic cycle and regenerates the catalyst.

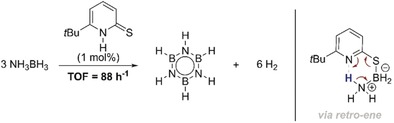

The controlled release of dihydrogen from ammonia borane (AB) with its H_2_ content of 19.7 wt % is of interest considering its potential use as a hydrogen‐storage material.^1^ Several transition metals catalyze the dehydrogenation of AB efficiently.^2^ Among the most effective catalysts are nickel carbene complexes and noble transition‐metal complexes with pincer‐type phosphine ligands, but iron pincer complexes have also proved to be effective.^3^, ^4^, ^5^ Since Wegner and co‐workers showed that this reaction can also be catalyzed by a bidentate Lewis acid, the dehydrogenation of AB by main group systems has attracted considerable attention.^6^, ^7^ Slootweg, Uhl, and co‐workers reported a phosphine/aluminium‐based frustrated Lewis Pair (FLP) that effects the stochiometric dehydrogenation of AB and the catalytic dehydrogenation of dimethylamine‐borane (DMAB).^8^ Aldridge et al. showed that a xanthene‐based FLP catalyzes hydrogen release from AB and provided evidence for a chain‐growth mechanism.^9^ However, the reported turnover frequencies (TOF) of 4 h^−1^ are moderate compared to transition‐metal catalysts. Earlier this year, the field was advanced by the report that a geometrically constrained phosphine‐borane FLP displays improved activity for the dehydrogenation of DMAB, but the catalyst showed only moderate activity regarding dehydrogenation of AB.^10^ For practical applications, an efficient and easily accessible organic catalyst is desirable. Dixon and co‐workers showed that strong Brønsted acids initiate the dehydrogenation of AB, presumably by protonation of the hydridic B−H group.^11^ We thus envisioned that an organic molecule possessing an acidic group and a basic site could serve as an organocatalyst for the dehydrogenation of AB by protonation of the BH_3_ group and deprotonation of the NH_3_ group (Scheme 1).

**Scheme 1:**
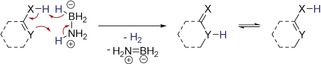
The working hypothesis of this research project: Dehydrogenation of AB by an organocatalyst through simultaneous protonation and deprotonation.

This organocatalyst would have to be able to revert to its initial form in order to form a catalytic cycle. 2‐Hydroxypyridine satisfies the criteria of an acidic OH group and a basic pyridine ring. Furthermore, the tautomers 2‐pyridone and 2‐hydroxypyridine are almost isoenergetic. We therefore considered 2‐pyridone as a suitable candidate for the catalytic dehydrogenation of AB. Aside from simple 2‐pyridone **1**, the sterically more encumbered 6‐*tert*‐butyl‐2‐pyridone (**2**) was tested as a catalyst. Furthermore, the more acidic thiopyridones **3** and **4** were used.^12^ We attempted the dehydrogenation of AB by reacting 1 mol % of the respective organocatalyst with AB at reflux in THF (Scheme 2). The results of the catalytic reactions are summarized in Table 1.

**Scheme 2:**
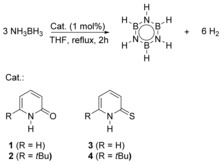
Dehydrogenation of AB by various pyridine derivatives.

**Table 1:** Results of the catalytic dehydrogenations of AB by pyridone derivatives (conditions shown in Scheme 2).

Catalyst	equiv H_2_^[a]^	TOF [h^−1^]^[b]^	conv. AB [%]^[c]^
**1**	0.06	3.2	13
**2**	0.62	30.8	28
**3**	0.10	4.8	17
**4**	1.76	88.0	99

[a] Based on the volumetric determination of H_2_. [b] Calculated as [H_2_]⋅[cat.]^−1^ h^−1^. [c] Determined by ^11^B NMR using a linear fit of the AB signal/integration ratio.

The parent pyridone **1** shows only moderate catalytic activity. However, 1 mol % of the sterically more encumbered 6‐*tert*‐butyl‐2‐pyridone (**2**) catalyzes hydrogen release from AB with a notably higher efficiency: 0.6 equivalents of hydrogen were liberated within 2 h, which corresponds to a TOF of 31 h^−1^. Borazine is the main product of this reaction, as demonstrated by ^11^B NMR. Thiopyridone **3** is less active than **2** but displays a slightly higher activity than parent pyridone **1**. This result indicates that the combination of steric demand and increased acidity should lead to an active catalyst. Indeed, 1 mol % 6‐*tert*‐butyl‐2‐thiopyridone (**4**) catalyzes the liberation of 1.8 equiv H_2_ from AB within 2 h, which corresponds to a TOF of 88 h^−1^. This is, to the best of our knowledge, hitherto the highest TOF for H_2_ release from AB reported for a metal‐free system. Analysis of the reaction mixture shows that AB is completely converted into borazine and polyborazylene.

At 120 °C in toluene, dehydrogenation of DMAB was efficiently catalyzed by **4** (1 mol %) within 4 h (Scheme 3). This experiment demonstrates the chemical robustness of **4**.

**Scheme 3:**
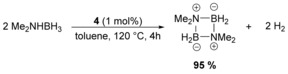
Dehydrogenation of DMAB catalyzed by **4**.

With these unexpected results in hand, we aimed for a mechanistic understanding regarding the mode of action by which **4** catalyzes hydrogen release from AB. To verify that **4** does not act as a Brønsted acid and initiates the dehydrogenation of AB through a chain‐growth mechanism, a catalytic reaction using 1 mol % thiophenol (which is more acidic than thiopyridone), was performed. This reaction led to the formation of B‐(cyclotriborazanyl)‐amine‐borane as the main product (Scheme 4). The observed TOF of 27 h^−1^ is significantly lower than that achieved with **4** as a catalyst. This corroborates the importance of catalyst bifunctionality, that is, the presence of the basic pyridine ring for the catalytic activity of **4**. It is tempting to attribute the higher activity of the *tert*‐butyl derivatives **2** and **4** to the destabilization of their respective dimers. The synthesis of **4** has been described previously, but its SCXRD structure has not been reported yet.^13^ Single crystals suitable for X‐ray analysis were obtained in the course of this study.^14^ The SCXRD structure is that of the thiopyridone dimer **4_2_** (Figure 1). The N−H⋅⋅⋅S distance of 3.46 Å is elongated by 0.17 Å compared to the *C*_2*h*_ symmetric dimer of **3**.^15^

**Figure 1:**
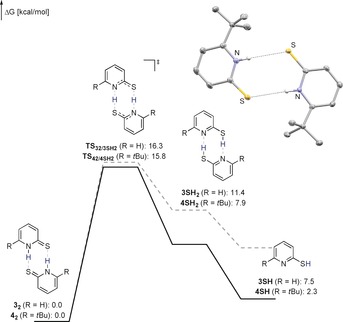
Gibbs free energies for the formation of **4SH** and **3SH** computed at the TightPNO‐DLPNO‐CCSD(T)/def2‐QZVPP//PBE0‐D3(BJ)/def2‐TZVP level. Solvent effects were implicitly considered using the SMD model for THF. The inset shows the molecular structure of **4_2_** derived from SCXRD (ellipsoids shown at 50 % probability, all hydrogen atoms attached to carbons omitted for clarity). Selected bond lengths and angles: N(H)⋅⋅⋅S: 3.46 Å, C−S: 1.70 Å, N−H⋅⋅⋅S: 173.2 °.^16^

**Scheme 4:**
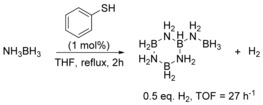
Attempted dehydrogenation of AB by thiophenol. The formal charges on nitrogen and boron are omitted for clarity.

The formation of monomeric **4SH** that is assumed to be the active catalyst was further investigated computationally at the SMD(THF)‐TightPNO‐DLPNO‐CCSD(T)/def2‐QZVPP//PBE0‐D3(BJ)/def2‐TZVP level (Figure 2).^17^, ^18^ Tautomerization of **4_2_** requires an activation energy of 15.8 kcal mol^−1^. The formation of **4SH** from **4_2_** is slightly endergonic. In comparison, the formation of **3SH** from **3_2_** is thermodynamically disfavored by 5.2 kcal mol^−1^. This result indicates that a ground‐state effect, that is the destabilization of **4_2_**, contributes to the activity of **4**.

**Figure 2:**
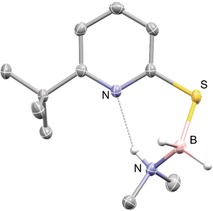
Molecular structure of **5_Me2_** derived from SCXRD (ellipsoids shown at 50 % probability, all hydrogen atoms attached to carbons omitted for clarity). Selected bond lengths and angles: N(H)⋅⋅⋅N: 2.8777(16) Å, B−S: 1.9107(18) Å, C‐S‐B: 102.61(7)°.

We then focused our attention on the detection of potential reactive intermediates. Upon monitoring a stoichiometric reaction of **4** with AB at 60 °C by NMR, the formation of the mercaptopyridine‐borane complex **5** was observed within 5 h. The NH_3_ group of **5** gives rise to a coalescent signal at 5.48 ppm in the ^1^H NMR spectrum. The BH_2_ group shows a signal at 2.62 ppm that integrates to two. A triplet at −13.3 ppm is observed by ^11^B NMR, which is a typical shift for a tetracoordinated borane.^19^ A NOE contact detected by NOSY NMR confirms spatial proximity between the NH_3_ group and the *tert*‐butyl group of the thiopyridone. Attempts to isolate **5** from solution were not successful. However, upon reaction of **4** with DMAB, a stable surrogate of **5** was obtained. The molecular structure of this surrogate **5_Me2_**, derived from SCXRD, supports the structural assignment of **5** (Figure 2). The structure shows a short N(H)⋅⋅⋅N hydrogen bond that indicates the possibility of an intramolecular proton transfer to the pyridine ring.

It is reasonable to assume that **5** is the product of a dehydrogenative coupling between the mercaptopyridine form of **4** and AB. That implies that the dehydrogenation of AB commences with this dehydrogenative coupling, which liberates the first equivalent H_2_ and yielding **5**.

When NH_3_BD_3_ was used as the substrate in the catalytic reaction, a kinetic isotope effect (KIE) of 1.20±0.15 was observed. This result is consistent with the computed transition state for the dehydrogenative coupling: While the S−H bond is ruptured, the B−H bond is only slightly distorted (Figure 3).^20^ Indeed, the computed KIE for the dehydrogenative coupling of 1.01 agrees favorable with experimentally observed KIE.^21^ A second AB molecule is required to stabilize the partial negative charge on the thiolate in the transition state.

**Figure 3:**
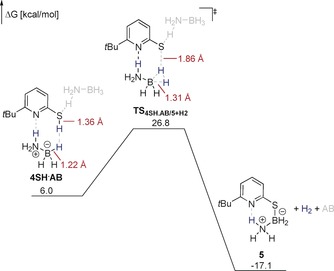
Gibbs free energies of the dehydrogenative coupling between **4** and AB computed at the TightPNO‐DLPNO‐CCSD(T)/def2‐QZVPP//PBE0‐D3(BJ)/def2‐TZVP level. All Gibbs free energies are given with respect to **4_2_** and AB_2_. Solvent effects were implicitly considered using the SMD model for THF. Formal charges on nitrogen and boron in the transition state are omitted for clarity.

Upon prolonged heating of a solution of **5**, the formation of borazine and regeneration of **4** was observed (Scheme 5). The reactivity of **5** was further investigated computationally (Figure 4). Proton transfer from the NH_3_ group to the pyridine ring and the concomitant breaking of the S−B bond requires a free activation energy of 11.0 kcal mol^−1^.^22^ This result indicates that **5** is not inert at 60 °C. However, the liberation of NH_2_BH_2_ and the regeneration of **4** are endergonic. Therefore, **5** can be observed in a stoichiometric reaction since it is thermodynamically stable with respect to the formation of NH_2_BH_2_ and **4**. The fact that **5** does react to borazine and **4** at elongated reaction times further indicates that the formation of borazine renders the stoichiometric reaction exergonic (Scheme 5).^23^ We note that, regarding the reorganization of π‐electron density, liberation of NH_2_BH_2_ from **5** can be described as an inorganic retro‐ene reaction.

**Figure 4:**
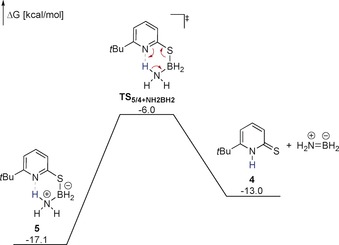
Gibbs free energies of the liberation of NH_2_BH_2_ from **5** computed at the TightPNO‐DLPNO‐CCSD(T)/def2‐QZVPP//PBE0‐D3(BJ)/def2‐TZVP level. Solvent effects were implicitly considered using the SMD model for THF. All Gibbs free energies are given with respect to **4_2_** and AB_2_. Formal charges on nitrogen and boron in the transitionstate are omitted for clarity.

**Scheme 5:**
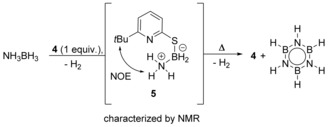
Intermediate **5** observed in the stoichiometric reaction of **4** with AB by NMR. Reaction conditions: [D_8_]THF, 60 °C.

Further evidence that the retro‐ene reaction is part of the catalytic cycle came from a stoichiometric experiment with **4** and ND_3_BH_3_. Upon reaction at elevated temperatures, the formation of borazine and incorporation of deuterium in **4** is observed (Scheme 6). A pronounced KIE of 2.4±0.3 is observed when ND_3_BH_3_ is used as the substrate in the catalytic reaction. Given the low barrier for the retro‐ene reaction, this KIE is presumably due to the deuterium incorporation in **4**. Indeed, the computed KIE for the dehydrogenative coupling (see Figure 3) starting from deuterated **4SD** and ND_3_BH_3_ is 3.2, which is in reasonable agreement with the experimentally observed KIE.

**Scheme 6:**
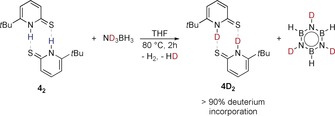
Deuterium incorporation into **4** upon reaction with ND_3_BH_3_.

Based on the experimental and computational investigations, we propose a mechanism for the dehydrogenation of AB by **4** that commences with tautomerization of **4** to the mercaptopyridine form **4SH,** presumably via its dimer (Scheme 7). A dehydrogenative coupling of AB with monomeric **4SH** yields borane **5**. The liberation of NH_2_BH_2_ regenerates monomeric **4**, which dimerizes and completes the catalytic cycle. However, contributions from an acid‐induced chain‐growth mechanism in the dehydrogenation of AB catalyzed by **4** cannot be excluded.

**Scheme 7:**
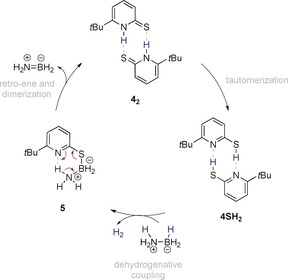
Proposed mechanism for the dehydrogenation of AB catalyzed by **4**.

In summary, we have documented that hydrogen release from AB is efficiently catalyzed by 6‐*tert*‐butyl‐2‐thiopyridone. Mechanistic investigations highlight the importance of bifunctionality of thiopyridone for the catalytic activity, while the *tert*‐butyl group facilitates the monomerization of **4**. The results reported herein are likely to stimulate the development of efficient organocatalysts for hydrogen‐storage applications.^24^

## Conflict of interest

The authors declare no conflict of interest.

## Supporting information

As a service to our authors and readers, this journal provides supporting information supplied by the authors. Such materials are peer reviewed and may be re‐organized for online delivery, but are not copy‐edited or typeset. Technical support issues arising from supporting information (other than missing files) should be addressed to the authors.

Supplementary
