# Supplementary material for: Efficient Organocatalytic Dehydrogenation of Ammonia Borane
Source: Angew Chem Int Ed Engl. 2019 Dec 10;59(4):1590–4. doi: 10.1002/anie.201910636 (PMC7003781; doi:10.1002/anie.201910636)
Supplement: Supplementary file 1 — Supplementary [file ANIE-59-1590-s001.pdf]

Angewandte Chemie
Eine Zeitschrift der Gesellschaft Deutscher Chemiker

Supporting Information

Efficient Organocatalytic Dehydrogenation of Ammonia Borane

*Max Hasenbeck, Jonathan Becker, and Urs Gellrich**

anie_201910636_sm_miscellaneous_information.pdf

1 Table of contents

2 General Specifications 4

3 General Procedure for the dehydrogenation of AB 4

4 Developing of reaction conditions for the catalytic dehydrogenation of AB and quantification ... 5

4.1 Solvent optimization 5

4.2 Quantification of conversion and residue of AB with 6-*tert*-butyl-2-pyridthione as catalyst. 5

4.3 Quantification of turnover frequency (TOF) based on H_2 production 6

4.4 Catalyst screening 7

5 Mechanistic investigations 10

5.1 Stoichiometric dehydrogenation of AB with 6-*tert*-butyl-2-thiopyridone 10

5.2 Full NMR Characterization of intermediate **5** 17

5.3 Stoichiometric dehydrogenation of dimethylammoniaborane (DMAB) with 6-*tert*-butyl-2-thiopyridone 18

5.4 Synthesis and characterization of surrogate intermediate $\mathbf{5_{Me2}}$ 25

6 X-ray analysis 26

6.1 Crystallographic data collection and processing 26

6.2 Crystallisation of surrogate intermediate $\mathbf{5_{Me2}}$ for X-Ray analysis 26

6.3 Crystallographic refinement of $\mathbf{5_{Me2}}$ 26

6.4 Crystallisation of 6-*tert*-butyl-2-thiopyridone dimer ($\mathbf{4_2}$) for X-Ray analysis 36

6.5 Crystallographic refinement of $\mathbf{4_2}$ 36

7 Kinetic isotope effect 44

8 Deuterium incooperation in 6-*tert*-butyl-2-thiopyridone 46

9 Catalytic dehydrogenation of DMAB 51

10 Characterization of intermediate **5** and a corresponding fragment ion by mass spectrometry

11 IR spectrum of 6-*tert*-butyl-2-thiopyridone in THF 54

12 Pathway without a second molecule of AB 55

13 Formation of Cyclotriborazane 56

14 Comparison of the computed PES of the dehydrogenation of AB catalyzed by **3** or **4** as catalyst

15 Dimerization of **4** 58

16 Additional NMR spectra 62

16.1 ^{11}B NMR spectra of catalysis experiments 62

16.2 NMR spectra of reaction mixture with intermediate **5** 76

16.3 NMR spectra of surrogate intermediate $\mathbf{5_{Me2}}$ 85

16.4 NMR spectra of the determination of the kinetic isotope effects 92

17 Computational details 116

17.1 Cartesian coordinates and energies 117

18 References 142

2 General Specifications

All manipulations with air and moisture sensitive compounds were carried out under a nitrogen atmosphere using standard Schlenk and glovebox techniques (nitrogen glovebox).

6-*tert*-butyl-2-thiopyridone was synthesized from 6-*tert*-butyl-2-pyridone, according to a literature known procedure.[1] The purification was done by flash column chromatography using DCM/methanol (99:1) as eluent and subsequent crystallization either in boiling *n*-hexane or by dissolving in DCM, layering with *n*-hexane (1:10 v/v) and storing overnight at -35° C.

6-*tert*-butyl-2-pyridone was synthesized from 3,3'-dimethyl-2-butanone, according to a literature known procedure.[2]

H_3NBD_3 was synthesized according to a modified literature known procedure.[3] 1.3 equivalents of $(NH_4)_2SO_4$ were used, and the reaction was monitored by ^{11}B NMR until only H_3NBD_3 was visible (about 18 h). The reaction was worked up as described and used without further purification.

D_3NBH_3 was synthesized by dissolving AB in MeOD, stirring under nitrogen for 20 minutes and evaporating the solvent at high vacuum. This procedure was done 3 times.

2-thiopyridone and 2-pyridone were commercially purchased.

Dichloroethane was dried by distillation under inert conditions over calciumhydride and stored over 4 Å molecular sieves in the glovebox at least one-day prior use.

All dry, non-deuterated solvents were, if commercially available, purchased by Acros Organics or Sigma Aldrich in a sealed bottle with a septum and stored (except of benzene) over molecular sieves.

Other dry solvents for synthesis were obtained from a MB-SPS-800 (M. Braun Inertgas-Systeme GmbH, Germany) solvent drying plant.

Deuterated solvents were distilled under inert conditions and kept in the glovebox over 4 Å molecular sieves. Ammonia borane (AB) was commercially purchased by TCI or Carbolution and stored in the glove box at -35 °C. Dimethylammonia borane was commercially purchased, sublimed under inert conditions and stored in the glove box at -35 °C.

NMR spectra were recorded on Bruker Avance II 200 MHz, Bruker Avance III HD 400 MHz, Bruker Avance II 400 MHz and Bruker Avance III HD 600 MHz spectrometers. ^{1}H and ^{13}C NMR chemical shifts are referenced to residual solvent resonance peaks. IR spectra were recorded on a Bruker Alpha FT-IR spectrometer with a resolution of 2 cm^{-1} and an accumulation of 32 scans. Mass spectra were recorded on an ESI-MS- Bruker Mikro-TOF mass spectrometer if not stated otherwise.

3 General Procedure for the dehydrogenation of AB

Inside the glovebox, a 15 mM stock solution of the catalyst in THF was prepared and stored at -35 °C. Inside a 120 mL Schlenk flask with a J Young valve and a stirring bar AB (23.2 mg, 0.75 mmol) was dissolved in 4.5 mL THF and the catalyst (0.5 mL of a 15 mM stock solution, 0.0075 mmol) was added. In the case of the blind experiments no catalyst and 5 mL of tetrahydrofuran were used. The flask was closed, taken out of the glove box and put into an oil bath preheated to 80 °C.

After 2 h the flask and solution were cooled to room temperature and the amount of generated H_2 was measured by using an inverted water-filled burette (for details see: Y.-Q. Zou, N. von Wolff, A. Anaby, Y. Xie, D. Milstein, *Nature Catalysis* **2019**, *2*, 415-422). The AB conversion was determined by ^{11}B NMR spectroscopy by using an aliquot of the reaction solution with a sealed glass capillary filled with benzene-d_6.

4 Developing of reaction conditions for the catalytic dehydrogenation of AB and quantification

4.1 Solvent optimization

For the solvent optimization THF, 1,4-dioxane, and 1,2-dichloroethane were tested. The experiments were done according to chapter 3 by using the corresponding solvent and a 15 mM stock solution of 6-*tert*-butyl-2-thiopyridone in the corresponding solvent. The performance of the catalyst in the respective solvent was evaluated by the amount of gas produced (see Table SI 1). The values are the averages of three experiments.

Table SI 1: Solvent optimization experiments.

solvent	gas volume [mL]
THF	36.1
1,4-dioxane	35.8
1,2-dichloroethane	22.7

Although THF and 1,4-dioxane give nearly identical values, we chose THF because of the lower boiling point, so the reaction is done under refluxing conditions.

4.2 Quantification of conversion and residue of AB with 6-*tert*-butyl-2-pyridthione as catalyst

The conversion of AB was checked by measuring a ^{11}B NMR spectrum of the reaction solution. For this purpose, an aliquot of the reaction solution was transferred to a NMR tube with a sealed glass capillary filled with benzene-d_6.

To quantify the conversion the absolute integral of AB in THF was fitted to a best-fit line of a dilution series of AB in THF. For this purpose, 8 stock solutions of AB in THF with the concentrations 0.02, 0.04, 0.05, 0.06, 0.07, 0.08, 0.10 and 0.14 M were prepared (Table SI 2 and Figure SI 1).

Table SI 2: Concentrations and absolute integrals of dilution series of AB in THF with benzene-d_6 glass capillary.

Absolute integral	concentration AB [M]
44990	0.02
70051	0.04
82053	0.05
118775	0.06
135260	0.07
143759	0.08
178810	0.10
251156	0.14

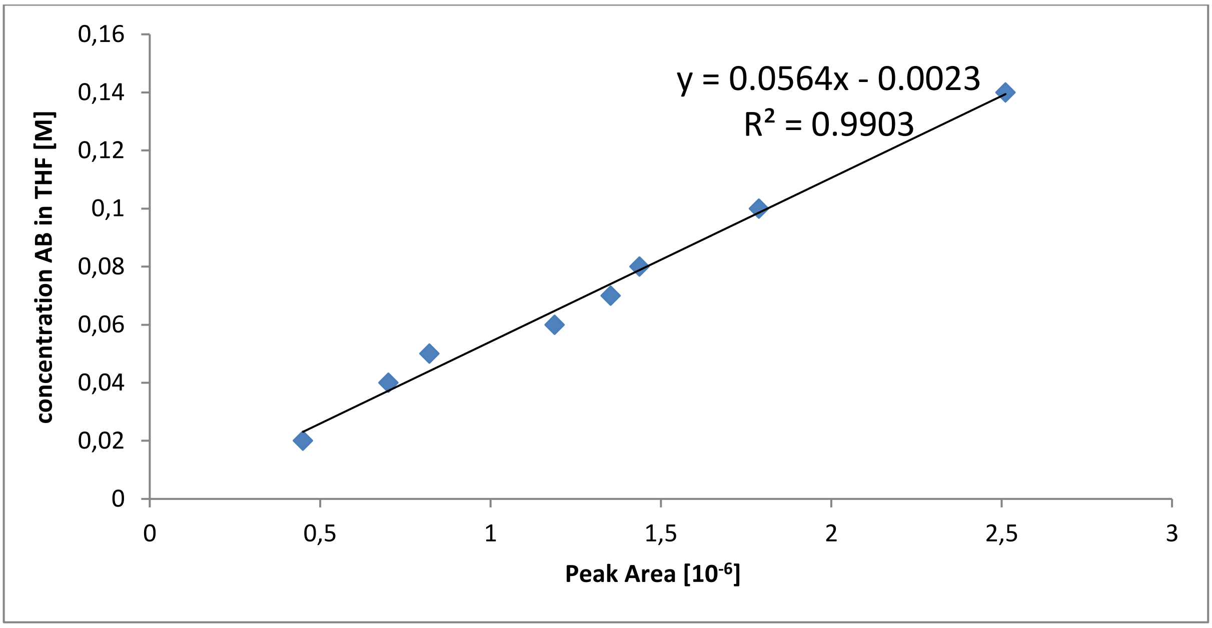

Figure SI 1: Best line fit with just equation and R^2 value of AB in THF with a benzene-d_6 filled glass capillary.

4.3 Quantification of turnover frequency (TOF) based on H_2 production

The amount of H_2 production was measured by using a method established by Milstein *et al.*[4] The gas volume was determined by an inverse water-filled burette.

For the TOF and produced equivalents of H_2 per equivalent AB the measured gas volumes were corrected by the gas volumes of the blind experiments. The amount of substance was calculated by using the ideal gas law (p = 101325 Pa, T = 295.15). The TOF was calculated as follows:

$$TOF = \frac{n\ (H2)}{n\ (catalyst) * t}$$

4.4 Catalyst screening

Several catalysts for the catalytic dehydrogenation of AB were tested (Scheme SI 1).

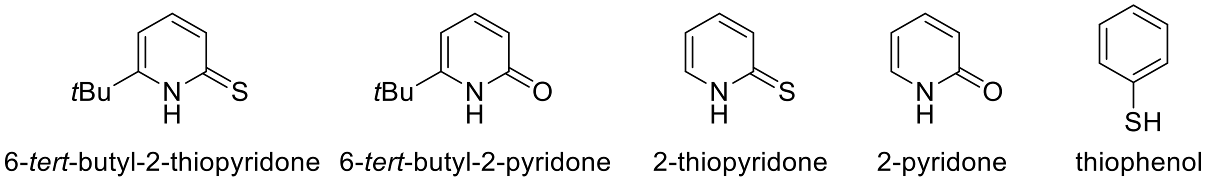

Scheme SI 1: Catalyst tested for the catalytic dehydrogenation of AB.

The blind experiments and experiments with 6-*tert*-butyl-2-thiopyridone were done three times. Every other experiment was done two times. A detailed overview is given in Table SI 3.

For the final values, the average of all separate experiments was used (see Table SI 4).

Table SI 3: Detailed overview of all tested catalysts for the dehydrogenation of AB.

catalyst	gas volume [mL]	gas volume [mL] (blind value corrected)	amount of H_2 [mmol] (blind value corrected)	equivalents H_2	TOF (based on H_2 evolution)	absolute integral of AB	concentration of AB in reaction solution [M]	AB conversion [%]
6-*tert*-butyl-2-pyridone	15.9	11.9	0.49	0.66	32.8	184144	0.102	32
6-*tert*-butyl-2-pyridone	14.5	10.5	0.43	0.58	28.9	191369	0.106	30
2-thiopyridone	5.9	1.9	0.08	0.10	5.2	209659	0.116	23
2-thiopyridone	5.6	1.6	0.07	0.09	4.4	225935	0.125	17
2-pyridone	5.2	1.2	0.05	0.07	3.3	214839	0.119	21
2-pyridone	5.1	1.1	0.05	0.06	3.0	240254	0.133	11
thiophenol	13.5	9.5	0.39	0.52	26.2	135094	0.074	51
thiophenol	14.0	10.0	0.41	0.55	27.5	137072	0.075	50
6-*tert*-butyl-2-thiopyridone	35.9	31.9	1.32	1.76	87.8	*traces*	*traces*	99
6-*tert*-butyl-2-thiopyridone	34.1	30.1	1.24	1.66	82.9	*traces*	*traces*	99
6-*tert*-butyl-2-thiopyridone	38.2	34.2	1.41	1.88	94.1	*traces*	*traces*	99
none	4.0	-	-	-	-	251121	0.139	7
none	5.0	-	-	-	-	237874	0.132	12
none	3.0	-	-	-	-	230526	0.128	15

Table SI 4: Average values of catalysis experiments.

catalyst	gas volume [mL]	gas volume [mL] (blind value corrected)	amount of H_2 [mmol] (blind value corrected)	equivalents H_2	TOF (based on H_2 evolution)	absolute integral of AB	concentration of AB in reaction solution [M]	AB conversion [%]
6-*tert*-butyl-2-pyridone	15.2	11.2	0.46	0.62	30.8	187757	0.104	31
2-thiopyridone	5.8	1.8	0.07	0.1	4.8	217797	0.121	20
2-pyridone	5.2	1.2	0.05	0.06	3.2	227547	0.126	16
thiophenol	13.8	9.8	0.40	0.54	26.8	136083	0.074	50
6-*tert*-butyl-2-thiopyridone	36.1	32.1	1.32	1.77	88.3	*traces*	*traces*	99

5 Mechanistic investigations

5.1 Stoichiometric dehydrogenation of AB with 6-*tert*-butyl-2-thiopyridone

Inside the glovebox 6-*tert*-butyl-2-thiopyridone (9.7 mg, 0.058 mmol) and AB (1.8 mg, 0.058 mmol) were dissolved in 0.4 mL THF-d_8 in a NMR tube with a J Young valve. The tube was taken out of the glovebox, and a ^{1}H, and ^{11}B NMR were recorded (Figure SI 2 and 3).

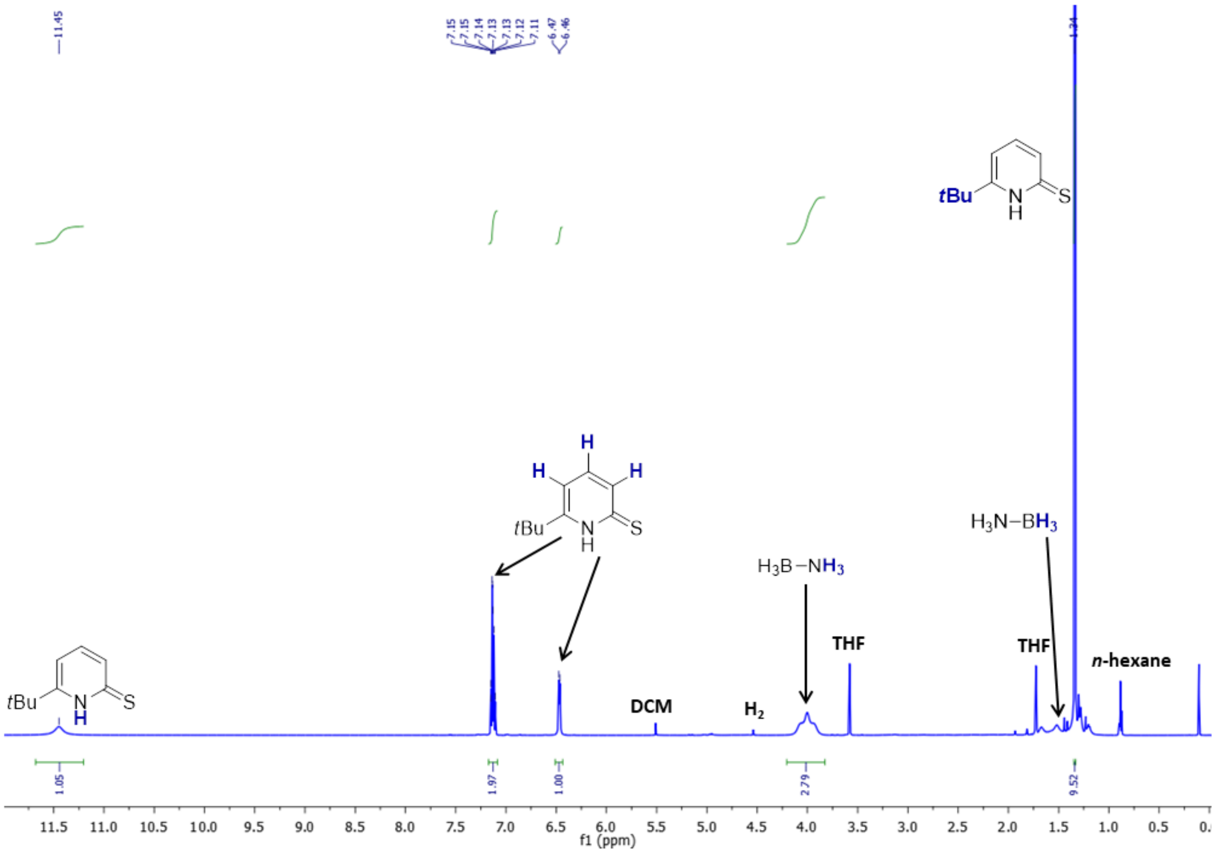

Figure SI 2: ^{1}H NMR spectrum of the stoichiometric reaction of 6-*tert*-butyl-2-thiopyridone and AB at room temperature before heating the reaction mixture (600 MHz, THF-d_8). Formal charges are omitted for clarity.

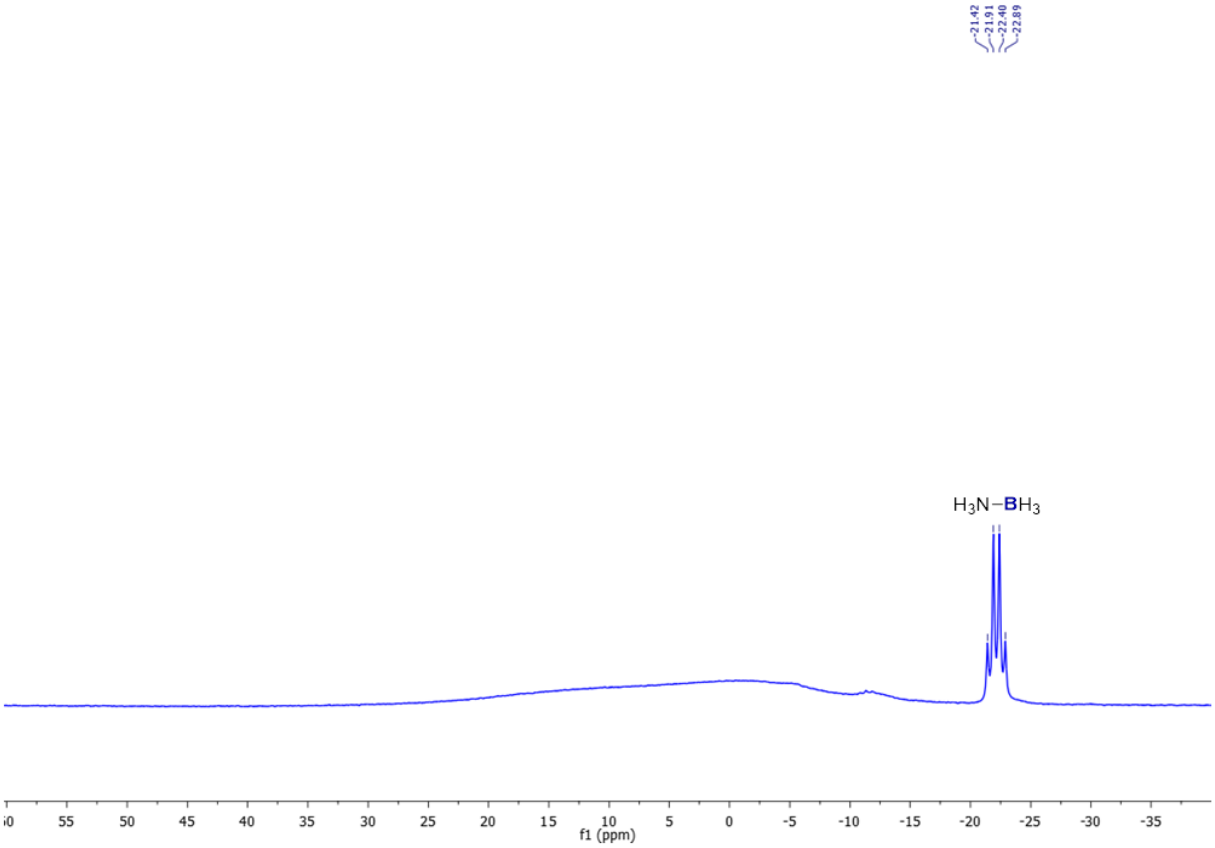

Figure SI 3: ^{11}B NMR spectrum of the stoichiometric reaction of 6-*tert*-butyl-2-thiopyridone and AB at room temperature before heating the reaction mixture (193 MHz, THF-d_8). Formal charges are omitted for clarity.

The reaction mixture was heated to 60 °C inside the NMR spectrometer, and the reaction progress was monitored by ^{1}H and ^{11}B NMR spectroscopy. During the reaction, new signals arise which could be assigned to the product of the dehydrogenative coupling **5** of AB and 6-*tert*-butyl-2-mercaptopyridine while the signals for 6-*tert*-butyl-2-thiopyridone and AB decrease in intensity (Figure SI 4 and 5, for a complete NMR characterization, see chapter 5.2). Additionally, the signal for H_2 increases in intensity.

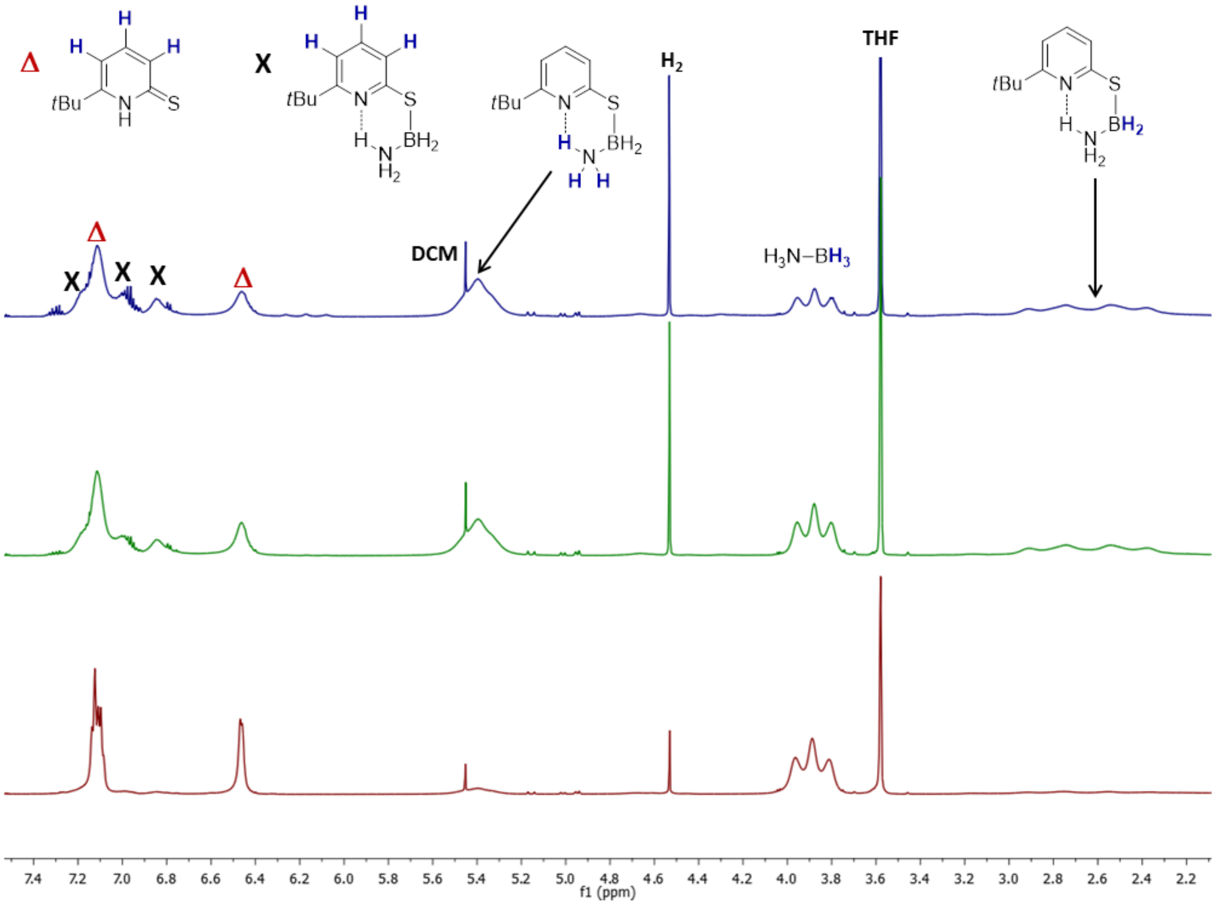

Figure SI 4: Excerpt of ^{1}H NMR spectra of reaction tracking of AB and 6-*tert*-butyl-2-thiopyridone at 60 °C after 0, 2 and 4 hours (lower, middle and upper spectrum, 600 MHz, THF-d_8). Formal charges are omitted for clarity.

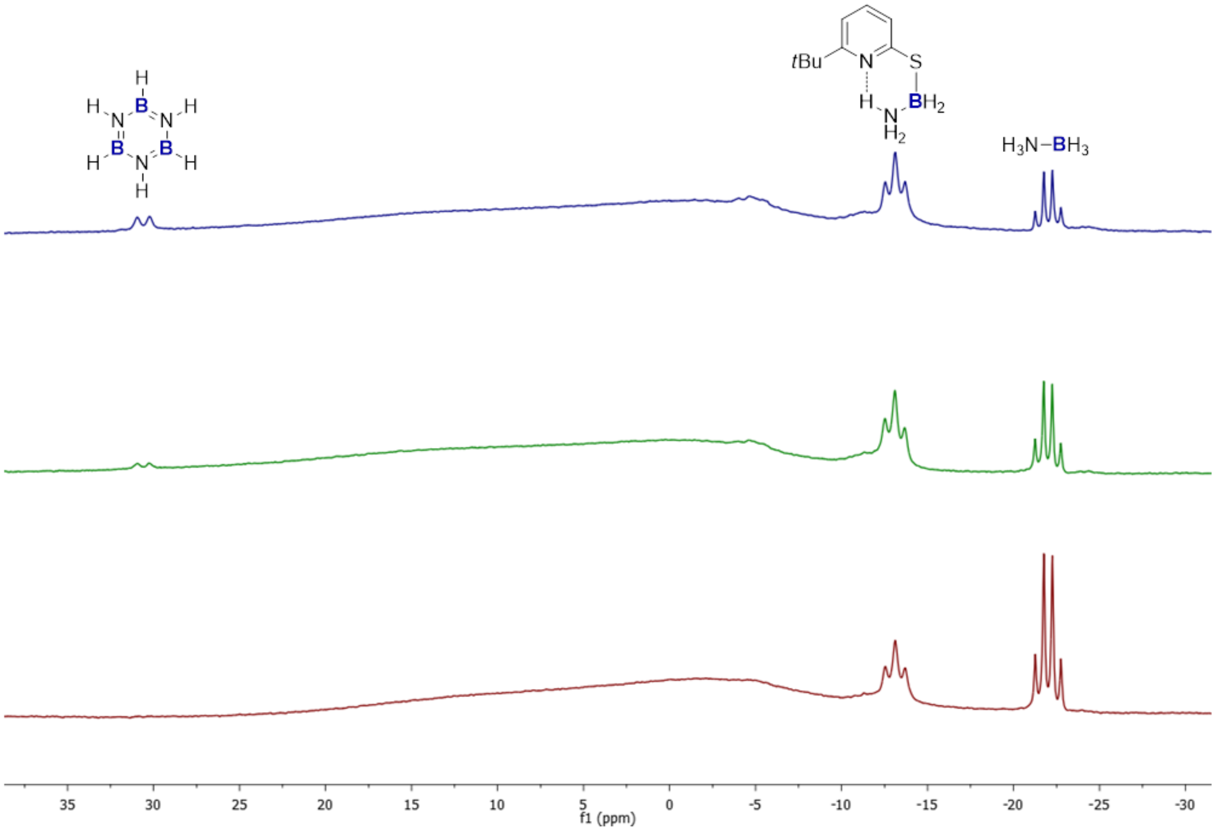

Figure SI 5: ^{11}B NMR spectra of reaction tracking of AB and 6-*tert*-butyl-2-thiopyridone at 60 °C after 1.5, 3.5, and 5 hours (lower, middle and upper spectrum, 193 MHz, THF-d_8). Formal charges are omitted for clarity.

The ^{11}B NMR spectra show that the signal for **5** increases in intensity over the reaction time to a certain point where it reaches a quasistationary state while the signals for AB decrease and the signal for the main dehydrogenation product of AB borazine increases. At this point, the reaction tracking was stopped. The sample was cooled to room temperature and the reaction mixture was characterized by ^{1}H, ^{11}B, HH COSY, ^{1}H-^{13}C HSQC, ^{1}H-^{13}C HMBC and HH NOESY NMR (for the complete NMR characterization of intermediate **5** see chapter 5.2).

After the characterization was completed, the sample was again heated to 60 °C inside the NMR spectrometer, and the reaction was again tracked by ^{1}H and ^{11}B NMR spectra. In summary, the ^{1}H NMR spectra show that over prolonged heating the signals for 6-*tert*-butyl-2-thiopyridone and AB decrease and the signals for intermediate **5** increase until a quasistationary stated is reached. After reaching the quasistationary state, the signals for AB and intermediate **5** decrease while the signals for 6-*tert*-butyl-2-thiopyridone increase, showing the regeneration of the catalyst (Figure SI 6).

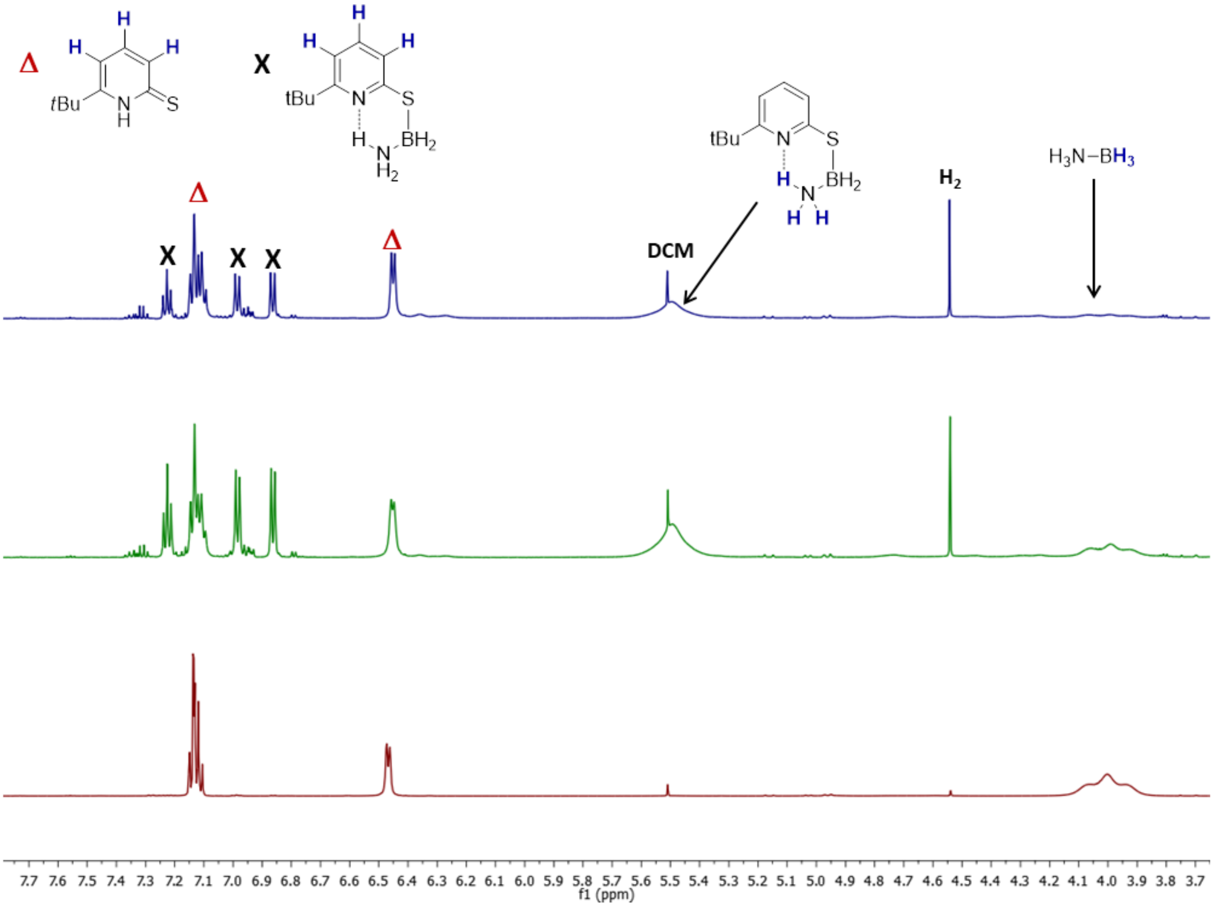

Figure SI 6: ^{1}H NMR spectra over the course of the stoichiometric reaction between 6-*tert*-butyl-2-thiopyridone and AB. The spectra were recorded at 298 K after being heated to 60 °C for 0 (lower spectrum), 5 h (middle spectrum) and 8 h (upper spectrum, 600 MHz, THF-d_8). Formal charges are omitted for clarity.

Equivalent observations could be made by ^{11}B NMR spectroscopy. The signals for AB continuously decrease while the signals for borazine increase. The signals for intermediate **5** increase until the quasistationary state is reached and subsequently decrease (Figure SI 7).

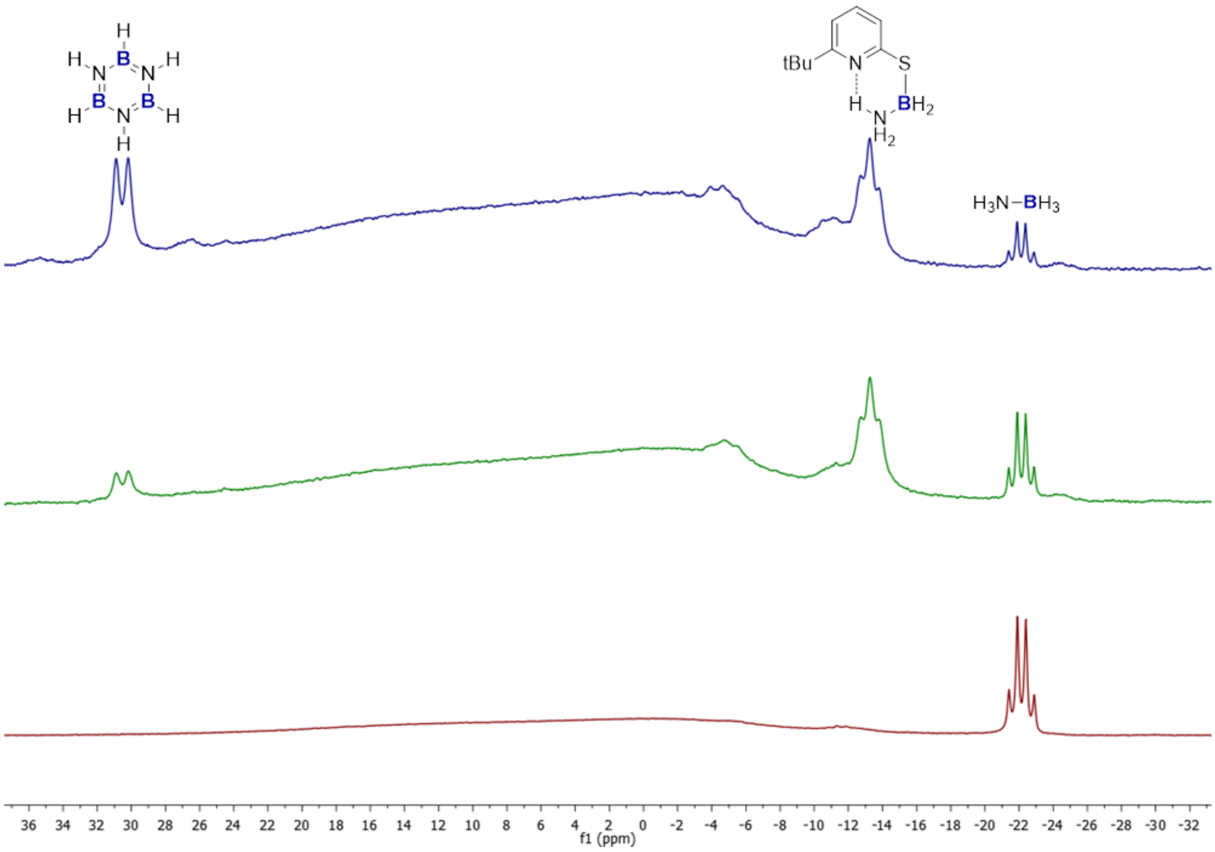

Figure SI 7: ^{11}B NMR spectra over the course of the stoichiometric reaction between 6-*tert*-butyl-2-thiopyridone and AB. The spectra were recorded at 298 K after being heated to 60 °C for 0 (lower spectrum), 5 h (middle spectrum) and 8 h (upper spectrum, 193 MHz, THF-d_8). Formal charges are omitted for clarity.

A reaction mixture with **5** obtained by an equivalent experiment was heated to 80 °C for 2 h. The ^{1}H NMR spectrum shows that **5** disappear while the catalyst is regenerated (Figure SI 8). The ^{11}B NMR spectrum shows the complete conversion of AB to borazine and polyborazylene (Figure SI 9).

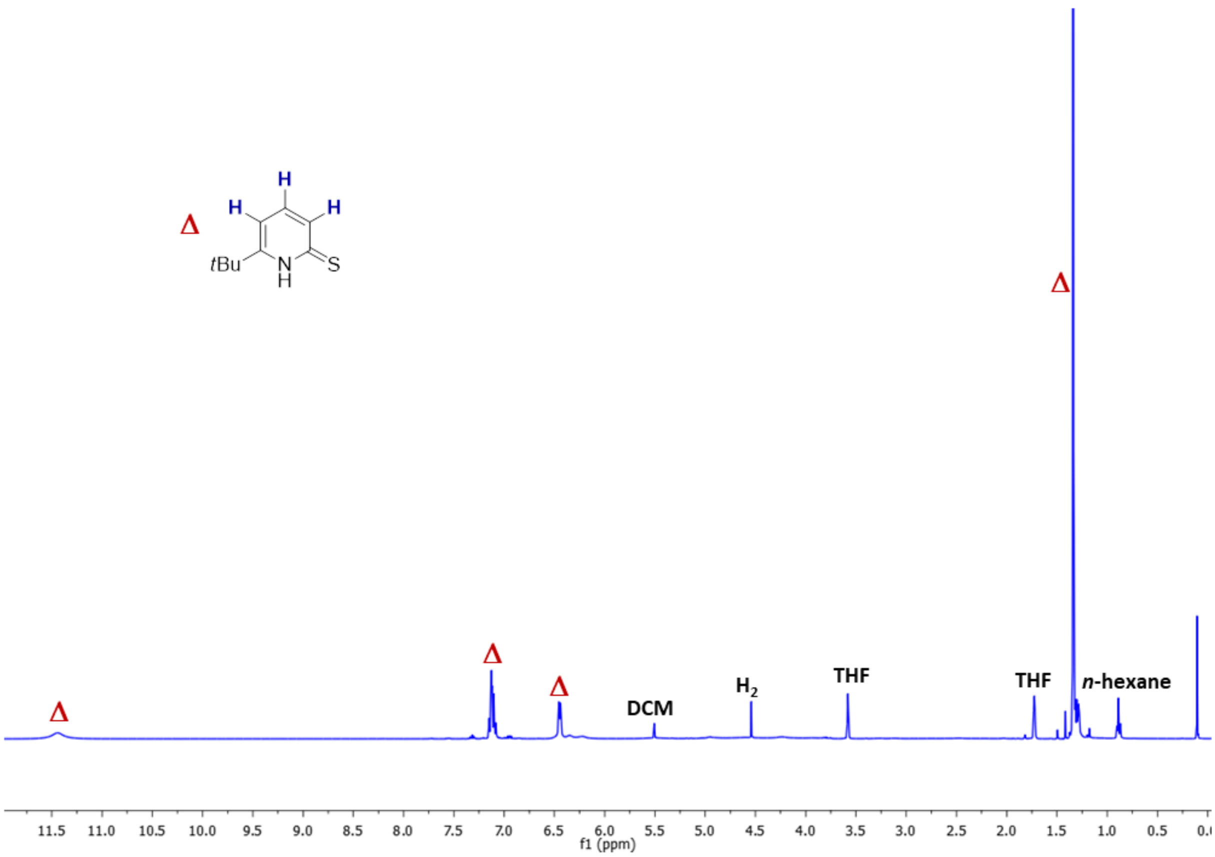

Figure SI 8: ^{1}H NMR spectrum of a reaction mixture of **5** being heated for 2 h at 80 °C (400 MHz, THF-d_8).

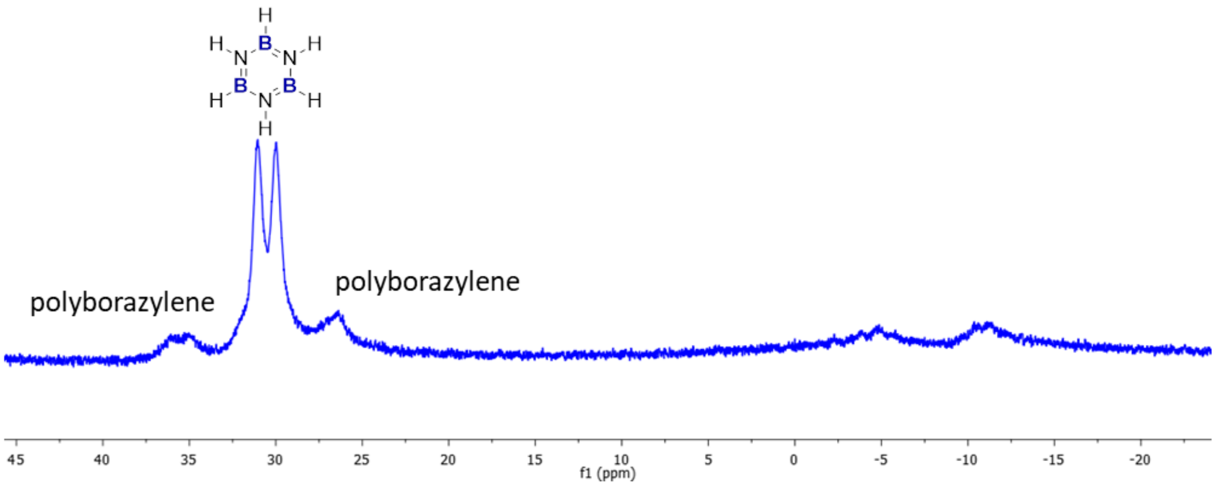

Figure SI 9: ^{11}B NMR spectrum of a reaction mixture of **5** after being heated for 2 h at 80 °C (128 MHz, THF-d_8). Formal charges are omitted for clarity.

Intermediate **5** could not be isolated presumably because of the irreversible loss of H_2NBH_2. Therefore, for further mechanistic investigations (*e.g.*, crystallographic analysis) the reaction between the surrogate DMAB and 6-*tert*-butyl-2-thiopyridone was examined (see chapter 5.3).

5.2 Full NMR Characterization of intermediate 5

The product of dehydrogenative coupling between 6-*tert*-butyl-2-thiopyridone and AB **5** was synthesized, as described in chapter 5.1. A full NMR characterization by ^1H, ^{11}B, HH COSY, ^1H-^{13}C HSQC, ^1H-^{13}C HMBC, HH NOESY was done directly from the reaction mixture *in situ* after the sample was cooled to room temperature (for additional spectra see chapter 7.2).

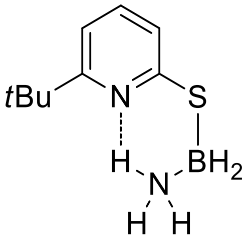

^1H NMR (600 MHz, THF-d_8) δ 7.23 (t, *J* = 7.8 Hz, *Py*-H, 1H), 6.98 (d, *J* = 7.9 Hz, *Py*-H, 1H), 6.86 (d, *J* = 7.7 Hz, *Py*-H, 1H), 5.48 (s, N*H*$_3$, 3H), 2.96 – 2.29 (m, B*H*$_2$, 2H), 1.30 (s, C*(CH$_3$)$_3$*, 9H).

^{13}C NMR (151 MHz, THF-d_8) δ 168.4 (Py-*C*), 168.1 (Py-*C*), 135.6 (Py-*C*), 123.4 (Py-*C*), 113.2 (Py-*C*), 37.7 (*C*(CH$_3$)$_3$), 30.5 (C*(CH$_3$)$_3$*).

^{11}B NMR (193 MHz, THF-d_8) δ -13.3 (t, $^1J_{B-H}$ = 105.1 Hz).

A crucial structural indication could be determined by the HH NOESY spectrum that shows the proximity between the NH_3 group of the AB moiety and the *tert*-butyl group of the mercaptopyridin moiety (Figure SI 10).

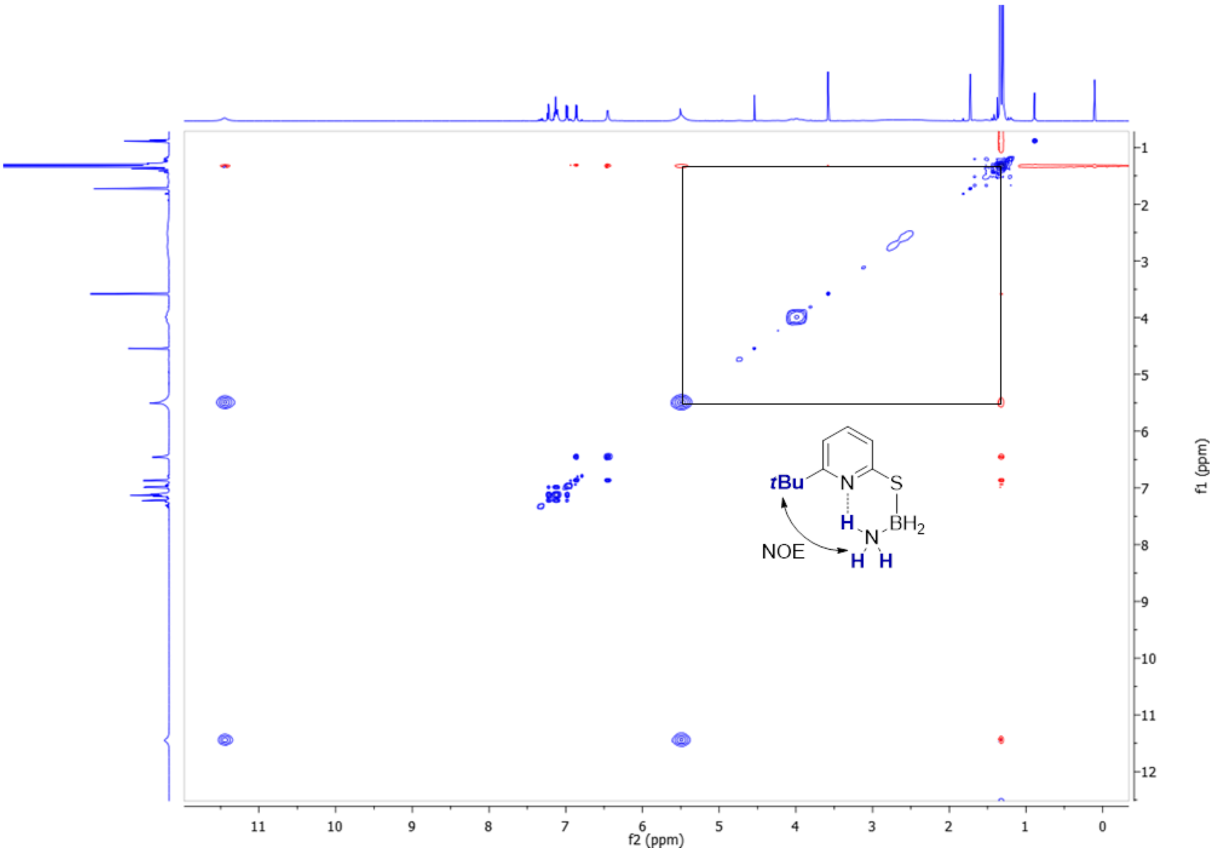

Figure SI 10: HH NOESY NMR spectrum of the reaction mixture of **5** and (600 MHz, benzene-d_6). Formal charges are omitted for clarity.

5.3 Stoichiometric dehydrogenation of dimethylammoniaborane (DMAB) with 6-*tert*-butyl-2-thiopyridone

To check if DMAB could be used as a surrogate for AB in the mechanistic investigations, we investigated the stoichiometric reaction of DMAB with 6-*tert*-butyl-2-thiopyridone.

Inside the glovebox, 6-*tert*-butyl-2-thiopyridone (10.0 mg, 0.06 mmol) and DMAB (3.5 mg, 0.06 mmol) were dissolved in 0.3 mL THF-d_8, transferred to a NMR tube with J Young valve and rinsed two times with 0.1 mL THF-d_8 each and heated to 70 °C overnight. The next day ^{1}H, ^{11}B, and COSY NMR were recorded. The ^{1}H NMR spectrum shows the formation of a new set of signals (ratio ca. 3:1) which could be assigned to structure **5**$_{\mathbf{Me2}}$ (Figure SI 11, for complete characterization, see chapter 5.4).

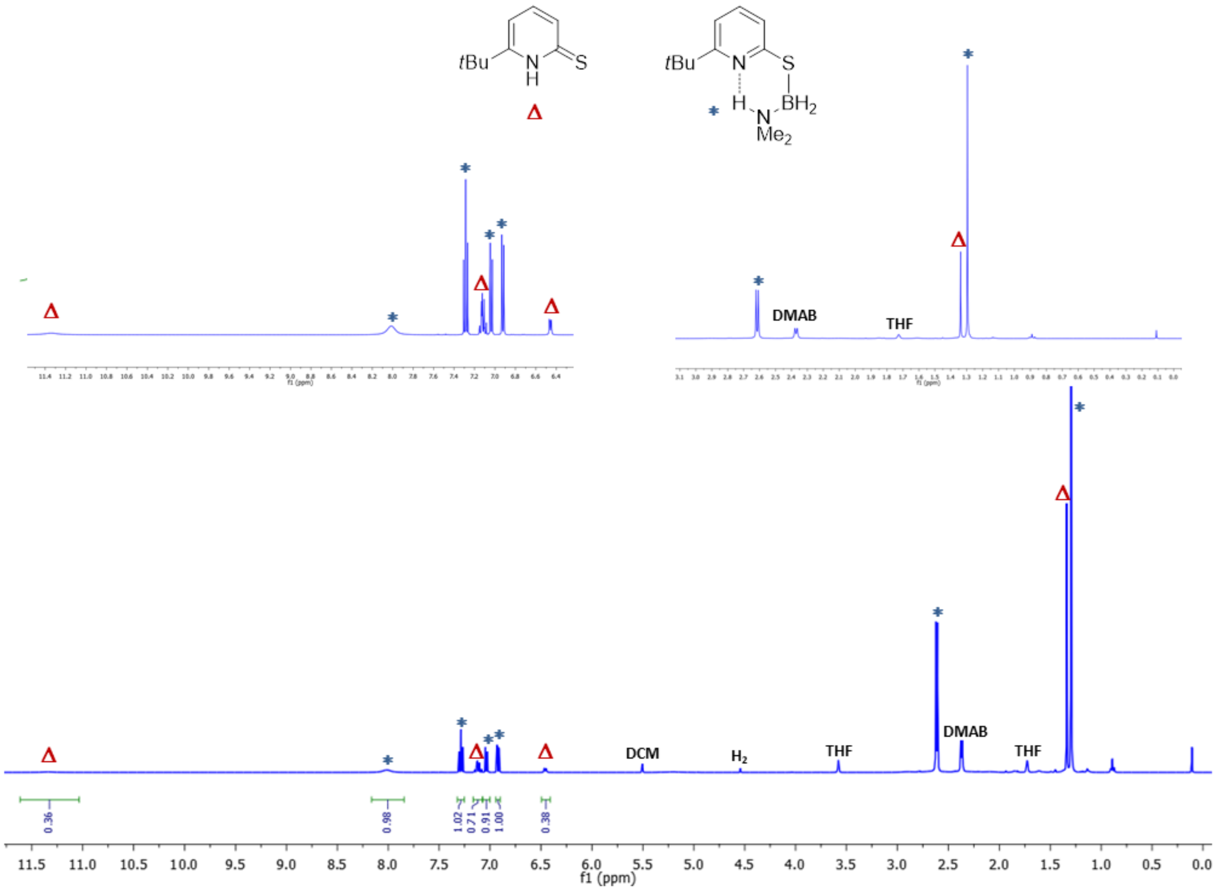

Figure SI 11: ^{1}H NMR spectrum of the stoichiometric dehydrogenation of DMAB with 6-*tert*-butyl-2-thiopyridone after heating overnight at 70 °C (400 MHz, THF-d_8). Formal charges are omitted for clarity.

The corresponding ^{11}B NMR shows only the two species DMAB and the intermediate $\mathbf{5_{Me2}}$ (Figure SI 12)

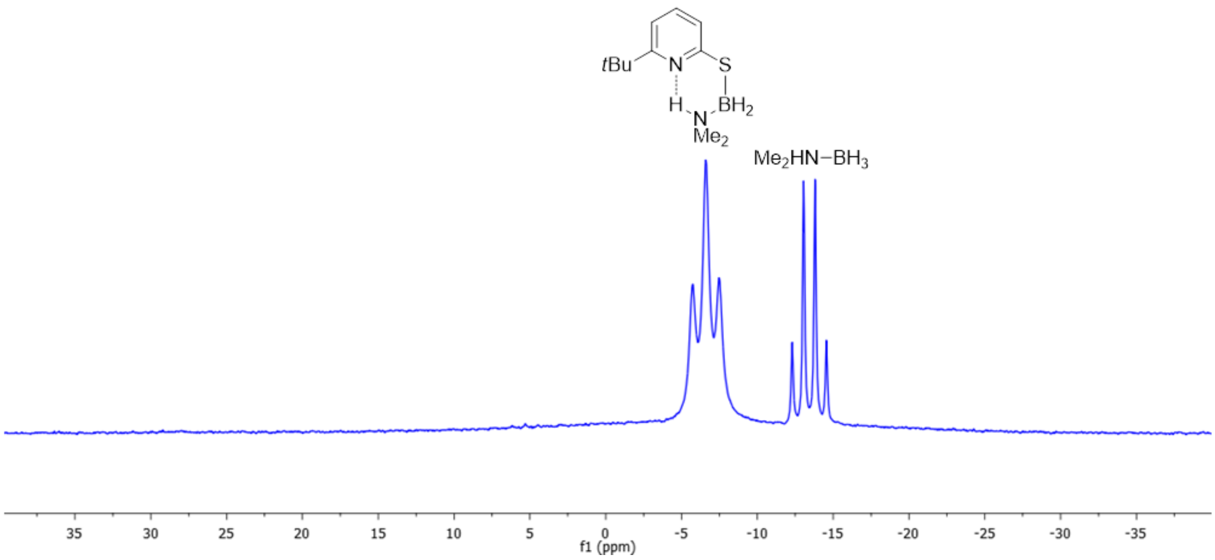

Figure SI 12: ^{11}B NMR spectrum of the stoichiometric dehydrogenation of DMAB with 6-*tert*-butyl-2-thiopyridone after heating overnight at 70 °C (128 MHz, THF-d_8). Formal charges are omitted for clarity.

Although the product of the dehydrogenative coupling of 6-*tert*-butyl-2-thiopyridone and DMAB could be observed, no formal product of the dehydrogenation of DMAB is visible in the NMR spectra even after prolonged heating.

The liberation of NMe_2BH_2 from $\mathbf{5_{Me2}}$ requires an activation energy of 20.5 kcal/mol, which explains its increased kinetic stability compared to **5** (Figure SI 13).

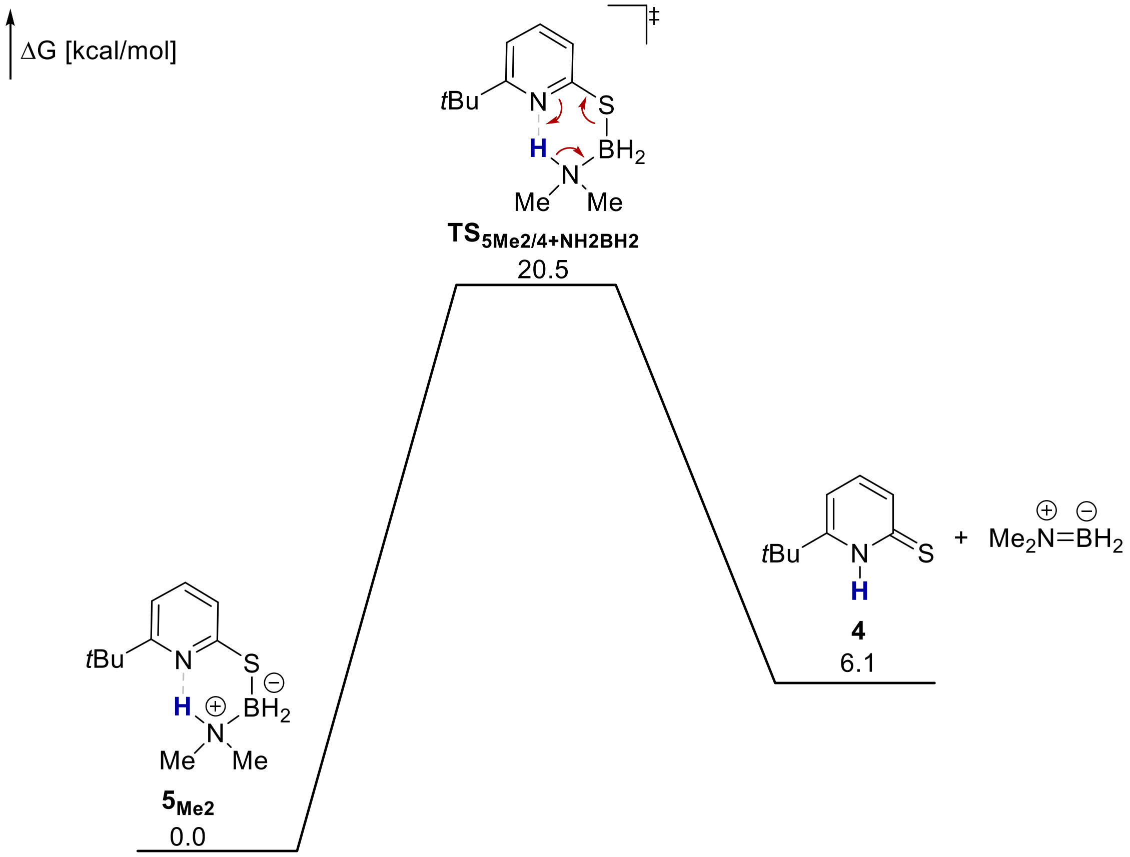

Figure SI 13: Liberation of NMe_2BH_2 from $\mathbf{5_{Me2}}$ (TightPNO-DLPNO-CCSD(T)/def2-QZVPP//PBE0-D3BJ/def2-TZVP. Solvent effects were implicitly considered with the SMD model for THF, (for computational details, see chapter 16). Formal charges on nitrogen and boron in the transition structure are omitted for clarity.

The computed thermodynamics of the reaction show that the stoichiometric dehydrogenation of DMAB is endergonic (Scheme SI 2).

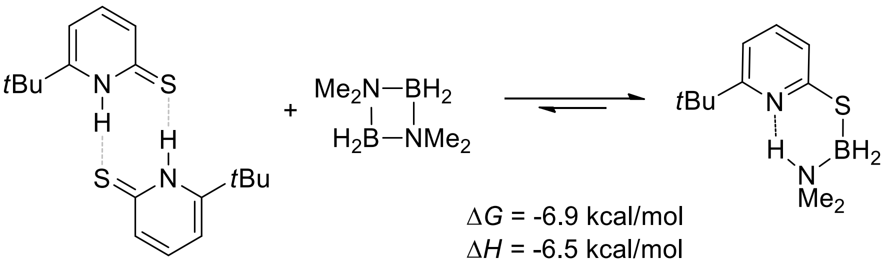

Scheme SI 2: Computed thermodynamics of the last step of the catalytic dehydrogenation of DMAB (Tight-PNO-DLPNO-CCSD(T)/def2-QZVPP//PBE0-D3(BJ)/def2-TZVP) (for computational details see chapter 16). Formal charges are omitted for clarity.

We envisioned that by adding 1 eq. of DMAB the reaction should be partially shifted to the left side and the four-membered NBNB ring consisting of two dehydrogenated DMAB molecules should be observable by means of ^{11}B NMR. For this experiment, the NMR tube was taken into the glovebox and 1 equivalent of DMAB (3.5 mg, 0.06 mmol) was added. The tube was heated to 80 °C overnight and the next day ^{1}H, ^{11}B and COSY spectra were recorded.

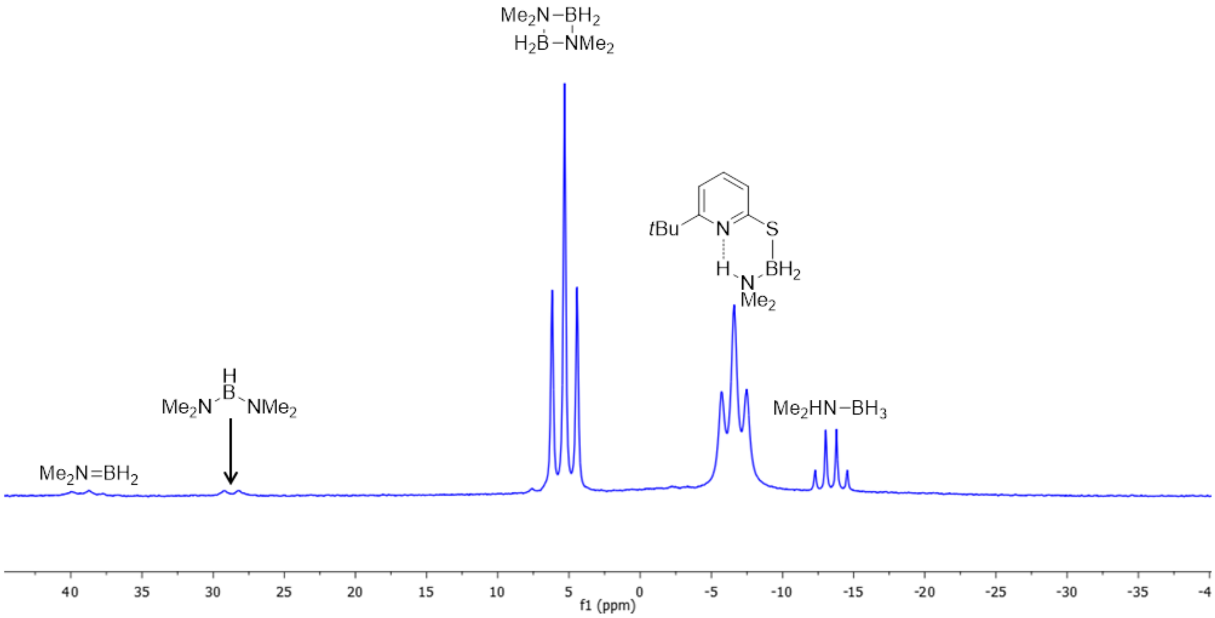

Figure SI 14: ^{11}B NMR spectrum of the stoichiometric dehydrogenation of DMAB with 6-*tert*-butyl-2-thiopyridone after adding 1 eq. of DMAB and heating overnight at 80 °C (128 MHz, THF-d_8). Formal charges are omitted for clarity.

The ^{11}B NMR (Figure SI 14) shows a new triplet at 5.3 ppm corresponding to the four-membered NBNB ring alongside traces of other dehydrogenation products of DMAB.

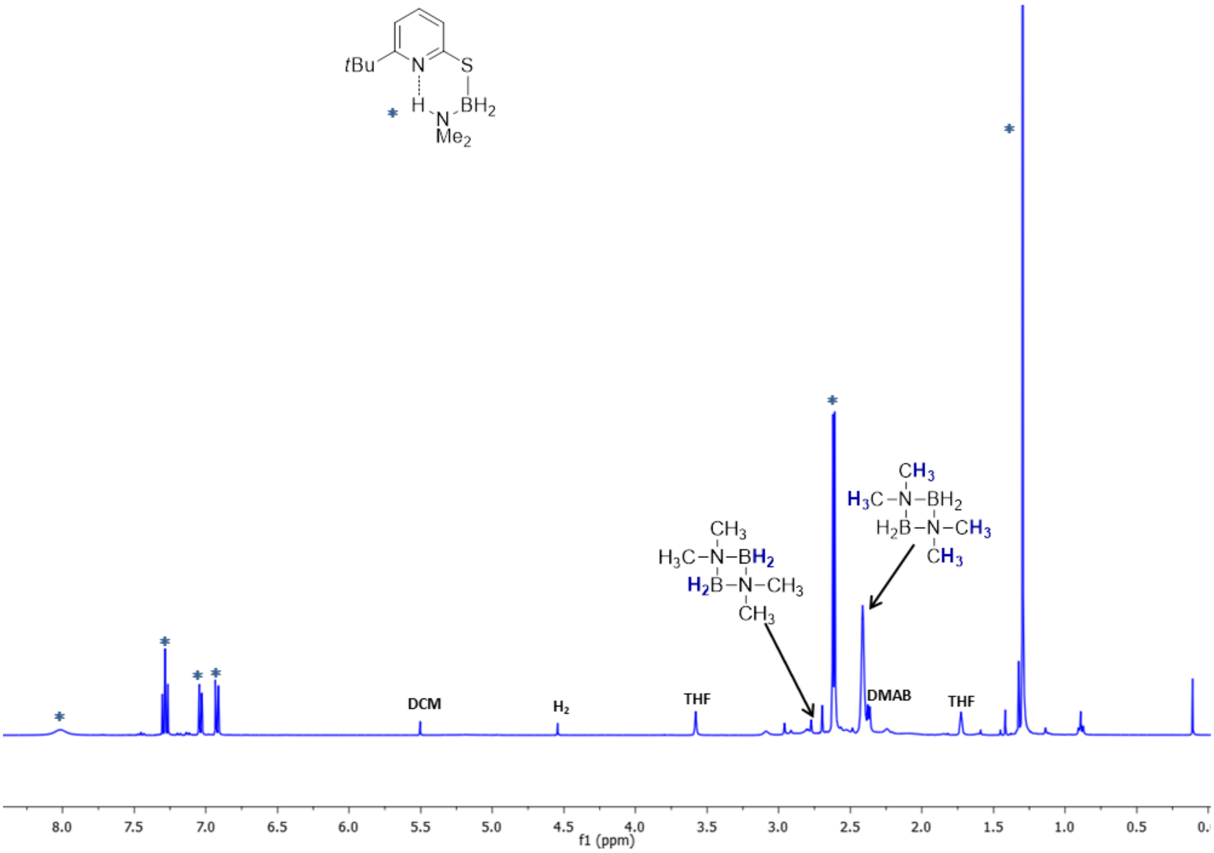

Figure SI 15: ^{1}H NMR spectrum of the stoichiometric dehydrogenation of DMAB with 6-*tert*-butyl-2-thiopyridone after adding 1 eq. of DMAB and heating overnight at 80 °C (400 MHz, THF-d_8). Formal charges are omitted for clarity.

The corresponding ^{1}H NMR spectrum (Figure SI 15) shows mainly the intermediate $\mathbf{5_{Me2}}$ while the signals for 6-*tert*-butyl-2-thiopyridone disappeared (assignment of the signals for the dehydrogenation products of DMAB are based on previously published literature).[5]

Additional information for the structural assignment of $\mathbf{5_{Me2}}$ could be obtained by X-Ray analysis (chapter 6) and HH NOESY NMR spectrum (Figure SI 16 and 17). The two excerpts show characteristic cross peak signals between the two methyl groups on the nitrogen and the *tert*-butyl-group of the surrogate intermediate $\mathbf{5_{Me}}$. Additionally, an exchange between the intermediate and 6-*tert*-butyl-2-thiopyridone is observable. Please note that the shown HH NOESY spectrum was obtained by another experiment between DMAB and 6-*tert*-butyl-2-thiopyridone (for details see chapter 5.4, and for the full HH NOESY spectrum see chapter 7.3).

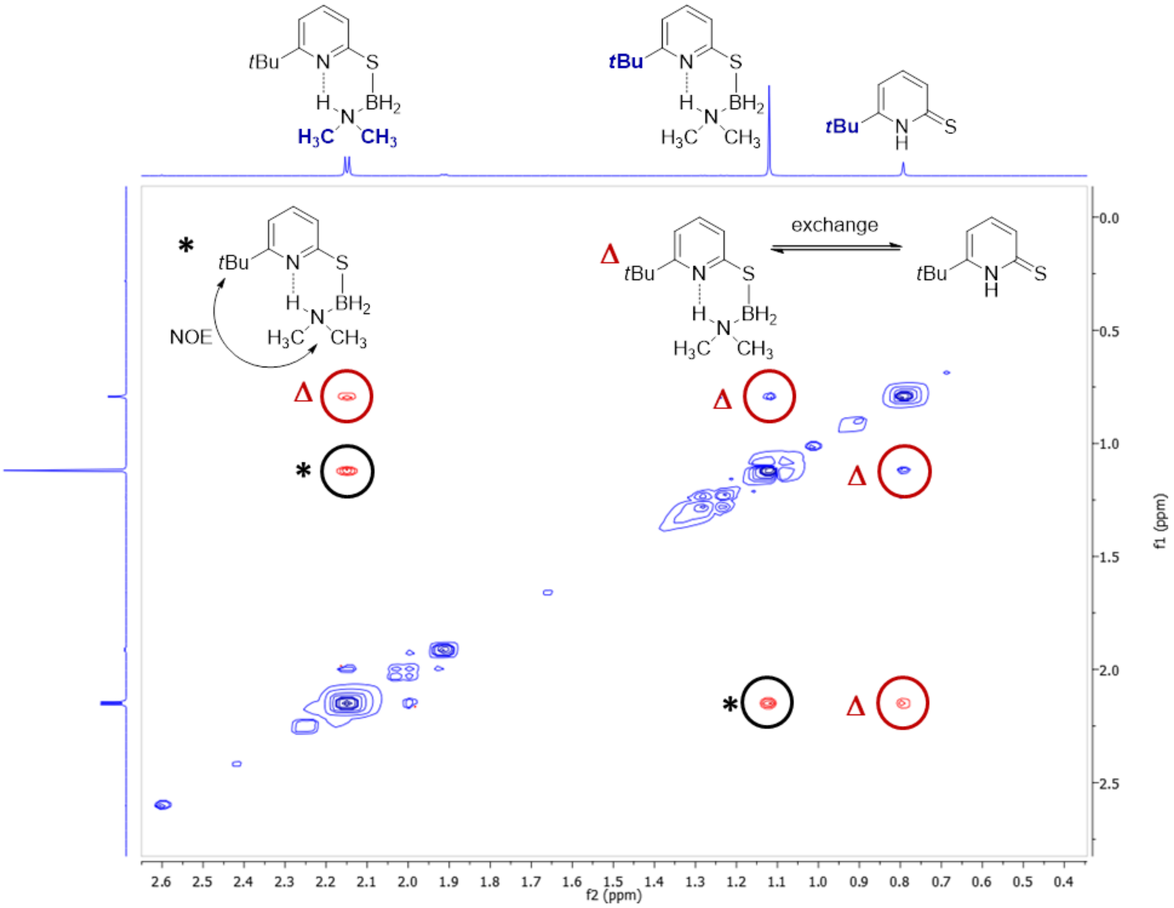

Figure SI 16: First excerpt of the HH NOESY NMR spectrum of surrogate intermediate $\mathbf{5_{Me2}}$ (600 MHz, benzene-d_6). Formal charges are omitted for clarity.

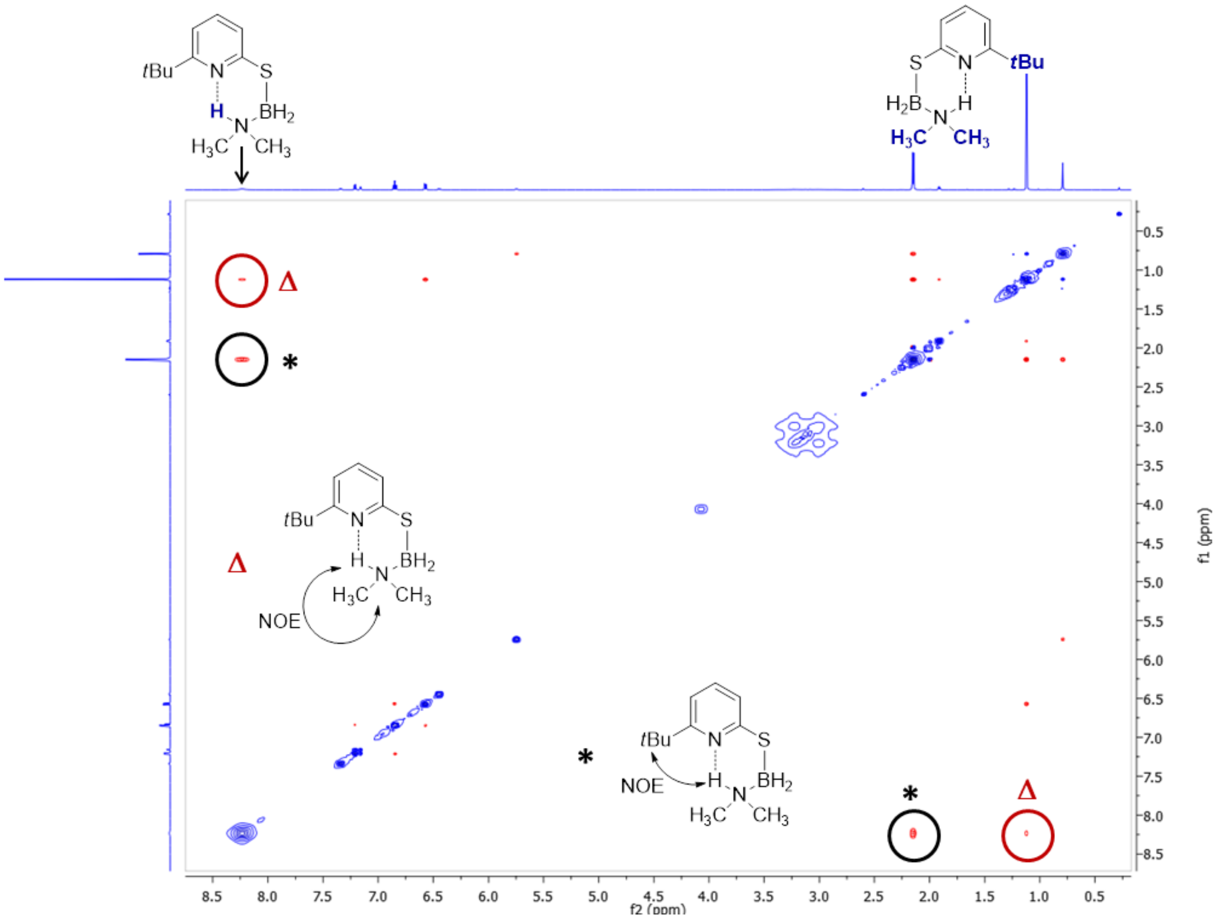

Figure SI 17: Second excerpt of the HH NOESY NMR spectrum of surrogate intermediate **5_{Me2}** (600 MHz, benzene-d_6). Formal charges are omitted for clarity.

5.4 Synthesis and characterization of surrogate intermediate 5_{Me2}

Inside the glovebox 6-*tert*-butyl-2-thiopyridone (30.0 mg, 0.18 mmol) and DMAB (10.6 mg, 0.18 mmol) were weighed into a 10 mL glass vial, dissolved in 0.4 mL benzene-d_6 and transferred into a NMR tube with J Young valve. The NMR tube was taken out of the glovebox and subjected to three freeze-pump-thaw cycles and heated for 4 d at 70 °C under passive vacuum. The formation of intermediate **5_{Me2}** was monitored by ^{1}H and ^{11}B NMR after every day during the reaction time. The reaction was stopped after ca 80% of **5_{Me2}** was produced, and a full NMR characterization was undertaken (for additional spectra see chapter 15.3).

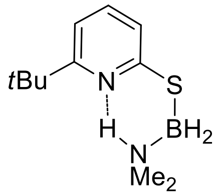

^{1}H NMR (600 MHz, benzene-d_6) δ 8.24 (s, N*H*, 1H), 7.21 (dd, *J* = 7.9, 0.8 Hz, Py-*H*, 1H), 6.85 (t, *J* = 7.8 Hz, Py-*H*, 1H), 6.57 (dd, *J* = 7.7, 0.8 Hz, Py-*H*, 1H), 3.12 (q, $^{1}J_{B\text{-}H}$ = 110.6 Hz, B*H*$_2$, 2H), 2.15 (d, *J* = 5.8 Hz, N*(CH$_3$)$_2$*, 6H), 1.12 (s, C*(CH$_3$)$_3$* ,9H).

^{13}C NMR (151 MHz, benzene-d_6) δ 167.7 (Py-*C*), 136.0 (Py-*C*), 124.0 (Py-*C*), 113.6 (Py-*C*), 39.6 (N*Me$_2$*), 37.0 (*C*(CH$_3$)$_3$), 30.3 (C*(CH$_3$)$_3$*).

^{11}B NMR (193 MHz, benzene-d_6) δ -6.1 (t, $^1J_{B-H}$ = 110.6 Hz)

HR-ESI-MS found (M+H$^+$), 225.1579, $C_{11}H_{21}BN_2S$ predicted (M+H$^+$), 225.1591

6 X-ray analysis

6.1 Crystallographic data collection and processing

Diffraction data were collected at low temperatures (100K) using ϕ- and ω-scans on a BRUKER D8 Venture system equipped with dual IµS microfocus sources, a PHOTON100 detector and an OXFORD CRYOSYSTEMS 700 low-temperature system. Mo-Kα radiation with wavelength 0.71073 Å and a collimating Quazar multilayer mirror were used. Semi-empirical absorption correction from equivalents was applied using SADABS-2016/2[6], and the structures were solved by the dual space algorithm implemented in SHELXT2014/5.[7] Refinement was performed against F^2 on all data by full-matrix least squares using SHELXL2018/3.[8] All non-hydrogen atoms were refined anisotropically and C-H hydrogen atoms were positioned at geometrically calculated positions and refined using a riding model. N-H and B-H hydrogen atoms were located in the difference map and were set to ideal distances. The isotropic displacement parameters of all hydrogen atoms were fixed to 1.2x or 1.5x (CH_3 hydrogens) the U_{eq} value of the atoms they are linked to.

The crystallographic data have been deposited with the Cambridge Crystallographic Data Centre as CCDC No. 1944361 and 1944362 and can be obtained free of charge at https://www.ccdc.cam.ac.uk/structures/.

6.2 Crystallisation of surrogate intermediate $\mathbf{5_{Me2}}$ for X-Ray analysis

Inside the glovebox 1 eq. of 6-*tert*-butyl-2-pyridone and 1 eq. of DMAB (ca. 100 mg overall) were dissolved in benzene and heated overnight at 60 °C. The solvent was removed at high vacuum. The residue was taken into the glovebox and extracted 5 times with 1 mL *n*-hexane each. The solution was stored at -35 °C overnight, filtered again to remove residual 6-*tert*-butyl-2-pyridone, concentrated and cooled -35 °C.

6.3 Crystallographic refinement of $\mathbf{5_{Me2}}$

The unit cell of $\mathbf{5}_{Me2}$ was determined using 9818 reflections and the structure was solved in the monoclinic space group $P2_1/n$. The asymmetric unit contains two independent molecules of $\mathbf{5_{Me2}}$.

Table SI 5: Crystal data and structure refinement for **5_{Me2}**.

CCDC No	1944361	
Empirical formula	C_{11} H_{21} B N_2 S	
Formula weight	224.17	
Temperature	100(2) K	
Wavelength	0.71073 Å	
Crystal system	Monoclinic	
Space group	$P2_1/n$	
Unit cell dimensions	a = 11.8014(7) Å	α= 90°.
	b = 13.4650(7) Å	β= 105.3735(16)°.
	c = 17.2579(9) Å	γ = 90°.
Volume	2644.3(3) $Å^3$	
Z	8	
Density (calculated)	1.126 Mg/m^3	
Absorption coefficient	0.217 mm^{-1}	
F(000)	976	
Crystal size	0.299 x 0.216 x 0.040 mm^3	
Theta range for data collection	2.343 to 26.021°.	
Index ranges	-14<=h<=14, -16<=k<=16, -21<=l<=21	
Reflections collected	161295	
Independent reflections	5204 [R(int) = 0.0715]	
Completeness to theta = 25.242°	100.0 %	
Absorption correction	Semi-empirical from equivalents	
Refinement method	Full-matrix least-squares on F^2	
Data / restraints / parameters	5204 / 6 / 299	
Goodness-of-fit on F^2	1.040	
Final R indices [I>2sigma(I)]	R_1 = 0.0344, wR_2 = 0.0809	
R indices (all data)	R_1 = 0.0464, wR_2 = 0.0878	
Largest diff. peak and hole	0.271 and -0.235 e.$Å^{-3}$	

Table SI 6: Atomic coordinates (x 10^4) and equivalent isotropic displacement parameters ($Å^2$x 10^3) for 5_{Me2}. U(eq) is defined as one third of the trace of the orthogonalized U^{ij} tensor.

	x	y	z	U(eq)
S(1)	2579(1)	5138(1)	1408(1)	23(1)
N(1)	4370(1)	5561(1)	2696(1)	17(1)
N(2)	4005(1)	6824(1)	1306(1)	22(1)
C(1)	3533(1)	4893(1)	2366(1)	18(1)
C(2)	3402(1)	4000(1)	2743(1)	22(1)
C(3)	4155(1)	3802(1)	3482(1)	24(1)
C(4)	5015(1)	4494(1)	3835(1)	22(1)
C(5)	5102(1)	5365(1)	3429(1)	18(1)
C(6)	5993(1)	6182(1)	3782(1)	20(1)
C(7)	6618(1)	6544(1)	3164(1)	28(1)
C(8)	5322(1)	7046(1)	4034(1)	31(1)
C(9)	6931(1)	5809(1)	4519(1)	30(1)
C(10)	4251(1)	7895(1)	1452(1)	30(1)
C(11)	4301(1)	6490(1)	565(1)	32(1)
B(1)	2691(1)	6547(1)	1322(1)	21(1)
S(21)	7481(1)	2639(1)	3799(1)	25(1)
N(21)	5768(1)	2062(1)	2514(1)	18(1)
N(22)	6189(1)	860(1)	3933(1)	21(1)
C(21)	6536(1)	2790(1)	2824(1)	19(1)
C(22)	6590(1)	3675(1)	2416(1)	24(1)
C(23)	5810(1)	3814(1)	1673(1)	27(1)
C(24)	5020(1)	3065(1)	1347(1)	24(1)
C(25)	5027(1)	2192(1)	1778(1)	19(1)
C(26)	4211(1)	1331(1)	1416(1)	22(1)
C(27)	4269(1)	492(1)	2023(1)	30(1)
C(28)	4601(1)	923(1)	695(1)	31(1)
C(29)	2932(1)	1692(1)	1136(1)	32(1)
C(30)	6126(1)	-238(1)	3959(1)	30(1)
C(31)	5673(1)	1309(1)	4546(1)	28(1)
B(21)	7490(1)	1233(1)	3977(1)	23(1)

Table SI 7: Bond lengths [Å] and angles [°] for $\mathbf{5_{Me2}}$.

S(1)-C(1)	1.7673(14)	S(21)-B(21)	1.9167(18)
S(1)-B(1)	1.9107(18)	N(21)-C(21)	1.3457(18)
N(1)-C(1)	1.3454(17)	N(21)-C(25)	1.3491(17)
N(1)-C(5)	1.3552(17)	N(22)-C(31)	1.4821(19)
N(2)-C(10)	1.4795(19)	N(22)-C(30)	1.4824(19)
N(2)-C(11)	1.4811(19)	N(22)-B(21)	1.598(2)
N(2)-B(1)	1.602(2)	N(22)-H(22)	0.874(13)
N(2)-H(2)	0.879(13)	C(21)-C(22)	1.395(2)
C(1)-C(2)	1.395(2)	C(22)-C(23)	1.379(2)
C(2)-C(3)	1.374(2)	C(22)-H(22A)	0.9500
C(2)-H(2A)	0.9500	C(23)-C(24)	1.386(2)
C(3)-C(4)	1.394(2)	C(23)-H(23)	0.9500
C(3)-H(3)	0.9500	C(24)-C(25)	1.391(2)
C(4)-C(5)	1.385(2)	C(24)-H(24)	0.9500
C(4)-H(4)	0.9500	C(25)-C(26)	1.530(2)
C(5)-C(6)	1.5322(19)	C(26)-C(27)	1.528(2)
C(6)-C(7)	1.529(2)	C(26)-C(29)	1.537(2)
C(6)-C(9)	1.5330(19)	C(26)-C(28)	1.539(2)
C(6)-C(8)	1.534(2)	C(27)-H(27A)	0.9800
C(7)-H(7A)	0.9800	C(27)-H(27B)	0.9800
C(7)-H(7B)	0.9800	C(27)-H(27C)	0.9800
C(7)-H(7C)	0.9800	C(28)-H(28A)	0.9800
C(8)-H(8A)	0.9800	C(28)-H(28B)	0.9800
C(8)-H(8B)	0.9800	C(28)-H(28C)	0.9800
C(8)-H(8C)	0.9800	C(29)-H(29A)	0.9800
C(9)-H(9A)	0.9800	C(29)-H(29B)	0.9800
C(9)-H(9B)	0.9800	C(29)-H(29C)	0.9800
C(9)-H(9C)	0.9800	C(30)-H(30A)	0.9800
C(10)-H(10A)	0.9800	C(30)-H(30B)	0.9800
C(10)-H(10B)	0.9800	C(30)-H(30C)	0.9800
C(10)-H(10C)	0.9800	C(31)-H(31A)	0.9800
C(11)-H(11A)	0.9800	C(31)-H(31B)	0.9800
C(11)-H(11B)	0.9800	C(31)-H(31C)	0.9800
C(11)-H(11C)	0.9800	B(21)-H(21A)	1.116(13)
B(1)-H(1A)	1.131(12)	B(21)-H(21B)	1.118(13)
B(1)-H(1B)	1.122(13)		
S(21)-C(21)	1.7662(14)	C(1)-S(1)-B(1)	102.61(7)

C(1)-N(1)-C(5)	118.61(12)	H(8B)-C(8)-H(8C)	109.5
C(10)-N(2)-C(11)	111.19(13)	C(6)-C(9)-H(9A)	109.5
C(10)-N(2)-B(1)	111.78(12)	C(6)-C(9)-H(9B)	109.5
C(11)-N(2)-B(1)	113.24(11)	H(9A)-C(9)-H(9B)	109.5
C(10)-N(2)-H(2)	108.8(11)	C(6)-C(9)-H(9C)	109.5
C(11)-N(2)-H(2)	109.1(11)	H(9A)-C(9)-H(9C)	109.5
B(1)-N(2)-H(2)	102.3(11)	H(9B)-C(9)-H(9C)	109.5
N(1)-C(1)-C(2)	122.63(12)	N(2)-C(10)-H(10A)	109.5
N(1)-C(1)-S(1)	119.00(10)	N(2)-C(10)-H(10B)	109.5
C(2)-C(1)-S(1)	118.35(10)	H(10A)-C(10)-H(10B)	109.5
C(3)-C(2)-C(1)	118.44(13)	N(2)-C(10)-H(10C)	109.5
C(3)-C(2)-H(2A)	120.8	H(10A)-C(10)-H(10C)	109.5
C(1)-C(2)-H(2A)	120.8	H(10B)-C(10)-H(10C)	109.5
C(2)-C(3)-C(4)	119.52(14)	N(2)-C(11)-H(11A)	109.5
C(2)-C(3)-H(3)	120.2	N(2)-C(11)-H(11B)	109.5
C(4)-C(3)-H(3)	120.2	H(11A)-C(11)-H(11B)	109.5
C(5)-C(4)-C(3)	119.21(13)	N(2)-C(11)-H(11C)	109.5
C(5)-C(4)-H(4)	120.4	H(11A)-C(11)-H(11C)	109.5
C(3)-C(4)-H(4)	120.4	H(11B)-C(11)-H(11C)	109.5
N(1)-C(5)-C(4)	121.58(12)	N(2)-B(1)-S(1)	108.63(10)
N(1)-C(5)-C(6)	115.54(12)	N(2)-B(1)-H(1A)	107.8(8)
C(4)-C(5)-C(6)	122.85(12)	S(1)-B(1)-H(1A)	108.2(8)
C(7)-C(6)-C(5)	111.07(11)	N(2)-B(1)-H(1B)	107.0(8)
C(7)-C(6)-C(9)	107.97(12)	S(1)-B(1)-H(1B)	110.7(8)
C(5)-C(6)-C(9)	111.29(12)	H(1A)-B(1)-H(1B)	114.4(11)
C(7)-C(6)-C(8)	109.69(13)	C(21)-S(21)-B(21)	103.98(7)
C(5)-C(6)-C(8)	107.79(11)	C(21)-N(21)-C(25)	118.76(12)
C(9)-C(6)-C(8)	109.01(12)	C(31)-N(22)-C(30)	110.57(12)
C(6)-C(7)-H(7A)	109.5	C(31)-N(22)-B(21)	114.21(12)
C(6)-C(7)-H(7B)	109.5	C(30)-N(22)-B(21)	111.55(12)
H(7A)-C(7)-H(7B)	109.5	C(31)-N(22)-H(22)	107.7(11)
C(6)-C(7)-H(7C)	109.5	C(30)-N(22)-H(22)	109.4(11)
H(7A)-C(7)-H(7C)	109.5	B(21)-N(22)-H(22)	102.9(11)
H(7B)-C(7)-H(7C)	109.5	N(21)-C(21)-C(22)	122.45(13)
C(6)-C(8)-H(8A)	109.5	N(21)-C(21)-S(21)	118.96(10)
C(6)-C(8)-H(8B)	109.5	C(22)-C(21)-S(21)	118.55(11)
H(8A)-C(8)-H(8B)	109.5	C(23)-C(22)-C(21)	118.53(14)
C(6)-C(8)-H(8C)	109.5	C(23)-C(22)-H(22A)	120.7
H(8A)-C(8)-H(8C)	109.5	C(21)-C(22)-H(22A)	120.7

C(22)-C(23)-C(24)	119.33(14)
C(22)-C(23)-H(23)	120.3
C(24)-C(23)-H(23)	120.3
C(23)-C(24)-C(25)	119.34(13)
C(23)-C(24)-H(24)	120.3
C(25)-C(24)-H(24)	120.3
N(21)-C(25)-C(24)	121.56(13)
N(21)-C(25)-C(26)	117.81(12)
C(24)-C(25)-C(26)	120.61(12)
C(27)-C(26)-C(25)	111.82(11)
C(27)-C(26)-C(29)	108.14(13)
C(25)-C(26)-C(29)	110.40(12)
C(27)-C(26)-C(28)	108.75(13)
C(25)-C(26)-C(28)	108.01(12)
C(29)-C(26)-C(28)	109.71(12)
C(26)-C(27)-H(27A)	109.5
C(26)-C(27)-H(27B)	109.5
H(27A)-C(27)-H(27B)	109.5
C(26)-C(27)-H(27C)	109.5
H(27A)-C(27)-H(27C)	109.5
H(27B)-C(27)-H(27C)	109.5
C(26)-C(28)-H(28A)	109.5
C(26)-C(28)-H(28B)	109.5
H(28A)-C(28)-H(28B)	109.5
C(26)-C(28)-H(28C)	109.5
H(28A)-C(28)-H(28C)	109.5
H(28B)-C(28)-H(28C)	109.5
C(26)-C(29)-H(29A)	109.5
C(26)-C(29)-H(29B)	109.5
H(29A)-C(29)-H(29B)	109.5
C(26)-C(29)-H(29C)	109.5
H(29A)-C(29)-H(29C)	109.5
H(29B)-C(29)-H(29C)	109.5
N(22)-C(30)-H(30A)	109.5
N(22)-C(30)-H(30B)	109.5
H(30A)-C(30)-H(30B)	109.5
N(22)-C(30)-H(30C)	109.5
H(30A)-C(30)-H(30C)	109.5
H(30B)-C(30)-H(30C)	109.5
N(22)-C(31)-H(31A)	109.5
N(22)-C(31)-H(31B)	109.5
H(31A)-C(31)-H(31B)	109.5
N(22)-C(31)-H(31C)	109.5
H(31A)-C(31)-H(31C)	109.5
H(31B)-C(31)-H(31C)	109.5
N(22)-B(21)-S(21)	109.76(10)
N(22)-B(21)-H(21A)	107.7(8)
S(21)-B(21)-H(21A)	107.7(8)
N(22)-B(21)-H(21B)	106.1(8)
S(21)-B(21)-H(21B)	111.8(8)
H(21A)-B(21)-H(21B)	113.7(12)

Table SI 8: Anisotropic displacement parameters (Å^{2}x 10^3) for **5$_{Me2}$**. The anisotropic displacement factor exponent takes the form: -2p^2[h^2 a*^{2}U^{11} + ... + 2 h k a* b* U^{12}].

	U^{11}	U^{22}	U^{33}	U^{23}	U^{13}	U^{12}
S(1)	19(1)	25(1)	20(1)	-2(1)	-3(1)	-3(1)
N(1)	15(1)	18(1)	17(1)	-1(1)	2(1)	0(1)
N(2)	18(1)	25(1)	19(1)	3(1)	0(1)	1(1)
C(1)	15(1)	20(1)	18(1)	-4(1)	3(1)	1(1)
C(2)	18(1)	19(1)	27(1)	-3(1)	5(1)	-3(1)
C(3)	25(1)	19(1)	28(1)	4(1)	7(1)	0(1)
C(4)	19(1)	25(1)	20(1)	3(1)	2(1)	1(1)
C(5)	14(1)	21(1)	17(1)	-2(1)	3(1)	2(1)
C(6)	18(1)	22(1)	18(1)	-1(1)	1(1)	-3(1)
C(7)	26(1)	34(1)	23(1)	0(1)	3(1)	-11(1)
C(8)	26(1)	26(1)	37(1)	-10(1)	4(1)	-4(1)
C(9)	23(1)	35(1)	24(1)	2(1)	-5(1)	-7(1)
C(10)	26(1)	27(1)	33(1)	6(1)	0(1)	-3(1)
C(11)	22(1)	46(1)	27(1)	0(1)	7(1)	0(1)
B(1)	15(1)	24(1)	22(1)	2(1)	1(1)	3(1)
S(21)	20(1)	28(1)	23(1)	-3(1)	-2(1)	-3(1)
N(21)	16(1)	19(1)	17(1)	-1(1)	3(1)	1(1)
N(22)	18(1)	26(1)	18(1)	4(1)	1(1)	2(1)
C(21)	15(1)	21(1)	21(1)	-2(1)	5(1)	1(1)
C(22)	24(1)	20(1)	29(1)	-2(1)	9(1)	-2(1)
C(23)	31(1)	21(1)	32(1)	7(1)	13(1)	4(1)
C(24)	24(1)	29(1)	20(1)	4(1)	5(1)	5(1)
C(25)	16(1)	24(1)	17(1)	-1(1)	5(1)	3(1)
C(26)	16(1)	27(1)	19(1)	-3(1)	1(1)	-1(1)
C(27)	29(1)	28(1)	29(1)	-3(1)	0(1)	-10(1)
C(28)	26(1)	42(1)	25(1)	-11(1)	4(1)	-3(1)
C(29)	18(1)	41(1)	33(1)	-6(1)	0(1)	1(1)
C(30)	30(1)	26(1)	32(1)	8(1)	5(1)	1(1)
C(31)	21(1)	40(1)	24(1)	4(1)	6(1)	6(1)
B(21)	15(1)	29(1)	24(1)	4(1)	2(1)	3(1)

Table SI 9: Hydrogen coordinates (x 10^4) and isotropic displacement parameters ($Å^2$x 10^3) for $\mathbf{5}_{\mathbf{Me2}}$.

	x	y	z	U(eq)
H(2)	4430(14)	6486(11)	1718(9)	26
H(2A)	2807	3540	2494	26
H(3)	4089	3197	3751	29
H(4)	5536	4369	4349	26
H(7A)	7014	5984	2984	43
H(7B)	7200	7051	3409	43
H(7C)	6041	6831	2702	43
H(8A)	4735	7300	3562	46
H(8B)	5875	7578	4268	46
H(8C)	4928	6812	4433	46
H(9A)	7311	5216	4375	44
H(9B)	6560	5644	4948	44
H(9C)	7521	6330	4707	44
H(10A)	5055	8038	1427	45
H(10B)	4165	8073	1984	45
H(10C)	3696	8283	1040	45
H(11A)	3833	6866	104	47
H(11B)	4127	5781	483	47
H(11C)	5138	6605	619	47
H(1A)	2082(13)	6785(12)	728(8)	26
H(1B)	2523(14)	6924(11)	1860(8)	26
H(22)	5784(14)	1071(12)	3462(8)	26
H(22A)	7151	4171	2645	29
H(23)	5813	4416	1387	32
H(24)	4480	3149	834	29
H(27A)	3718	-34	1776	45
H(27B)	4058	751	2496	45
H(27C)	5069	223	2184	45
H(28A)	4102	359	461	47
H(28B)	5421	705	874	47
H(28C)	4527	1445	289	47
H(29A)	2874	2228	743	48
H(29B)	2682	1938	1599	48

H(29C)	2424	1140	887	48
H(30A)	5307	-444	3883	45
H(30B)	6601	-476	4482	45
H(30C)	6429	-521	3531	45
H(31A)	5705	2034	4512	42
H(31B)	6119	1093	5082	42
H(31C)	4853	1096	4449	42
H(21A)	8046(13)	1088(12)	4598(8)	28
H(21B)	7774(14)	814(11)	3502(9)	28

Table SI 10: Hydrogen bonds for $\mathbf{5}_{\mathbf{Me2}}$ [Å and °].

D-H...A	d(D-H)	d(H...A)	d(D...A)	<(DHA)
N(2)-H(2)...N(1)	0.879(13)	2.114(15)	2.8777(16)	144.8(15)
N(22)-H(22)...N(21)	0.874(13)	2.108(14)	2.8671(16)	144.9(15)

6.4 Crystallisation of 6-*tert*-butyl-2-thiopyridone dimer (4_2) for X-Ray analysis

6-*tert*-butyl-2-pyridone was purified beforehand by column chromatography, dried and taken into the glove box. 350 mg were dissolved in 1 mL DCM and filtered through a Whatman filter into a 10 mL glass vial. The solution was carefully layered with 10 mL *n*-hexane and stored at -35 °C overnight.

6.5 Crystallographic refinement of 4_2

The unit cell for $\mathbf{4_2}$ was determined using 9973 reflections and the structure was solved in the trigonal space group $R\overline{3}$. Two molecules of **4** were found in the asymmetric unit, connected via hydrogen bonds and forming the dimer $\mathbf{4_2}$. Additionally, heavily disordered solvent molecules were found in the unit cell. It was not possible to refine these, so SQUEEZE[9] as implemented in Platon[10] was used to include a solvent model into the refinement. SQUEEZE identified three solvent containing voids in the unit cell, located at 0, 0, 0; 1/3, 2/3, 0.41 and 2/3, 1/3, 0.08. Each void contains the equivalent of about 144 electrons in 580Å^3. For the unit cell this results in an overall solvent accessible volume of 1740Å^3 with 420 electrons.

Table SI 11:. Crystal data and structure refinement for **4_2**.

CCDC No	1944362	
Empirical formula	$C_9 H_{13} N S$	
Formula weight	167.26	
Temperature	100(2) K	
Wavelength	0.71073 Å	
Crystal system	Trigonal	
Space group	$R\bar{3}$	
Unit cell dimensions	a = 34.7255(9) Å	α= 90°.
	b = 34.7255(9) Å	β= 90°.
	c = 9.2195(3) Å	γ = 120°.
Volume	9628.0(6) $Å^3$	
Z	36	
Density (calculated)	1.039 Mg/m^3	
Absorption coefficient	0.248 mm^{-1}	
F(000)	3240	
Crystal size	0.469 x 0.054 x 0.035 mm^3	
Theta range for data collection	2.311 to 28.700°.	
Index ranges	-46<=h<=46, -46<=k<=46, -12<=l<=12	
Reflections collected	84937	
Independent reflections	5536 [R(int) = 0.1060]	
Completeness to theta = 25.242°	99.9 %	
Absorption correction	Semi-empirical from equivalents	
Refinement method	Full-matrix least-squares on F^2	
Data / restraints / parameters	5536 / 2 / 211	
Goodness-of-fit on F^2	1.036	
Final R indices [I>2sigma(I)]	R_1 = 0.0377, wR_2 = 0.0854	
R indices (all data)	R_1 = 0.0577, wR_2 = 0.0933	
Largest diff. peak and hole	0.317 and -0.264 e.$Å^{-3}$	

Table SI 12: Atomic coordinates (x 10^4) and equivalent isotropic displacement parameters (Å^{2}x 10^3) for **4_2**. U(eq) is defined as one third of the trace of the orthogonalized U^{ij} tensor.

	x	y	z	U(eq)
S(1)	4467(1)	5498(1)	4896(1)	20(1)
N(1)	4597(1)	5802(1)	7618(1)	17(1)
C(1)	4673(1)	5902(1)	6175(2)	19(1)
C(2)	4935(1)	6358(1)	5836(2)	31(1)
C(3)	5078(1)	6670(1)	6906(2)	37(1)
C(4)	4970(1)	6547(1)	8363(2)	28(1)
C(5)	4732(1)	6107(1)	8725(2)	19(1)
C(6)	4616(1)	5928(1)	10266(2)	20(1)
C(7)	4870(1)	5686(1)	10637(2)	26(1)
C(8)	4113(1)	5610(1)	10413(2)	28(1)
C(9)	4751(1)	6309(1)	11351(2)	32(1)
S(11)	4132(1)	4686(1)	8508(1)	19(1)
N(11)	4174(1)	4422(1)	5814(1)	15(1)
C(11)	4112(1)	4317(1)	7258(2)	15(1)
C(12)	4026(1)	3886(1)	7621(2)	19(1)
C(13)	4014(1)	3603(1)	6576(2)	22(1)
C(14)	4076(1)	3730(1)	5113(2)	20(1)
C(15)	4154(1)	4144(1)	4730(2)	15(1)
C(16)	4209(1)	4313(1)	3179(2)	17(1)
C(17)	4684(1)	4705(1)	2969(2)	20(1)
C(18)	3868(1)	4462(1)	2867(2)	20(1)
C(19)	4133(1)	3946(1)	2093(2)	24(1)

Table SI 13: Bond lengths [Å] and angles [°] for **4_2**.

S(1)-C(1)	1.6930(15)	C(16)-C(17)	1.5375(19)
N(1)-C(1)	1.3672(18)	C(16)-C(18)	1.5392(19)
N(1)-C(5)	1.3735(18)	C(17)-H(17A)	0.9800
N(1)-H(1)	0.841(13)	C(17)-H(17B)	0.9800
C(1)-C(2)	1.412(2)	C(17)-H(17C)	0.9800
C(2)-C(3)	1.361(2)	C(18)-H(18A)	0.9800
C(2)-H(2)	0.9500	C(18)-H(18B)	0.9800
C(3)-C(4)	1.402(2)	C(18)-H(18C)	0.9800
C(3)-H(3)	0.9500	C(19)-H(19A)	0.9800
C(4)-C(5)	1.367(2)	C(19)-H(19B)	0.9800
C(4)-H(4)	0.9500	C(19)-H(19C)	0.9800
C(5)-C(6)	1.522(2)		
C(6)-C(7)	1.532(2)	C(1)-N(1)-C(5)	125.40(12)
C(6)-C(9)	1.532(2)	C(1)-N(1)-H(1)	115.7(11)
C(6)-C(8)	1.536(2)	C(5)-N(1)-H(1)	118.9(11)
C(7)-H(7A)	0.9800	N(1)-C(1)-C(2)	115.75(13)
C(7)-H(7B)	0.9800	N(1)-C(1)-S(1)	121.29(11)
C(7)-H(7C)	0.9800	C(2)-C(1)-S(1)	122.96(12)
C(8)-H(8A)	0.9800	C(3)-C(2)-C(1)	120.40(15)
C(8)-H(8B)	0.9800	C(3)-C(2)-H(2)	119.8
C(8)-H(8C)	0.9800	C(1)-C(2)-H(2)	119.8
C(9)-H(9A)	0.9800	C(2)-C(3)-C(4)	121.13(15)
C(9)-H(9B)	0.9800	C(2)-C(3)-H(3)	119.4
C(9)-H(9C)	0.9800	C(4)-C(3)-H(3)	119.4
S(11)-C(11)	1.6973(14)	C(5)-C(4)-C(3)	119.60(14)
N(11)-C(15)	1.3662(17)	C(5)-C(4)-H(4)	120.2
N(11)-C(11)	1.3683(18)	C(3)-C(4)-H(4)	120.2
N(11)-H(11)	0.854(13)	C(4)-C(5)-N(1)	117.58(13)
C(11)-C(12)	1.4118(19)	C(4)-C(5)-C(6)	124.95(13)
C(12)-C(13)	1.363(2)	N(1)-C(5)-C(6)	117.44(12)
C(12)-H(12)	0.9500	C(5)-C(6)-C(7)	108.94(12)
C(13)-C(14)	1.401(2)	C(5)-C(6)-C(9)	110.67(12)
C(13)-H(13)	0.9500	C(7)-C(6)-C(9)	108.42(13)
C(14)-C(15)	1.3688(19)	C(5)-C(6)-C(8)	110.45(12)
C(14)-H(14)	0.9500	C(7)-C(6)-C(8)	110.27(12)
C(15)-C(16)	1.5207(19)	C(9)-C(6)-C(8)	108.06(13)
C(16)-C(19)	1.5358(19)	C(6)-C(7)-H(7A)	109.5

C(6)-C(7)-H(7B)	109.5
H(7A)-C(7)-H(7B)	109.5
C(6)-C(7)-H(7C)	109.5
H(7A)-C(7)-H(7C)	109.5
H(7B)-C(7)-H(7C)	109.5
C(6)-C(8)-H(8A)	109.5
C(6)-C(8)-H(8B)	109.5
H(8A)-C(8)-H(8B)	109.5
C(6)-C(8)-H(8C)	109.5
H(8A)-C(8)-H(8C)	109.5
H(8B)-C(8)-H(8C)	109.5
C(6)-C(9)-H(9A)	109.5
C(6)-C(9)-H(9B)	109.5
H(9A)-C(9)-H(9B)	109.5
C(6)-C(9)-H(9C)	109.5
H(9A)-C(9)-H(9C)	109.5
H(9B)-C(9)-H(9C)	109.5
C(15)-N(11)-C(11)	125.43(12)
C(15)-N(11)-H(11)	121.3(11)
C(11)-N(11)-H(11)	113.2(11)
N(11)-C(11)-C(12)	115.65(12)
N(11)-C(11)-S(11)	121.27(10)
C(12)-C(11)-S(11)	123.07(11)
C(13)-C(12)-C(11)	120.75(13)
C(13)-C(12)-H(12)	119.6
C(11)-C(12)-H(12)	119.6
C(12)-C(13)-C(14)	120.58(13)
C(12)-C(13)-H(13)	119.7
C(14)-C(13)-H(13)	119.7
C(15)-C(14)-C(13)	119.86(13)
C(15)-C(14)-H(14)	120.1
C(13)-C(14)-H(14)	120.1
N(11)-C(15)-C(14)	117.70(13)
N(11)-C(15)-C(16)	117.60(12)
C(14)-C(15)-C(16)	124.69(12)
C(15)-C(16)-C(19)	110.87(12)
C(15)-C(16)-C(17)	109.51(11)
C(19)-C(16)-C(17)	108.47(12)
C(15)-C(16)-C(18)	109.55(11)
C(19)-C(16)-C(18)	108.28(11)
C(17)-C(16)-C(18)	110.13(11)
C(16)-C(17)-H(17A)	109.5
C(16)-C(17)-H(17B)	109.5
H(17A)-C(17)-H(17B)	109.5
C(16)-C(17)-H(17C)	109.5
H(17A)-C(17)-H(17C)	109.5
H(17B)-C(17)-H(17C)	109.5
C(16)-C(18)-H(18A)	109.5
C(16)-C(18)-H(18B)	109.5
H(18A)-C(18)-H(18B)	109.5
C(16)-C(18)-H(18C)	109.5
H(18A)-C(18)-H(18C)	109.5
H(18B)-C(18)-H(18C)	109.5
C(16)-C(19)-H(19A)	109.5
C(16)-C(19)-H(19B)	109.5
H(19A)-C(19)-H(19B)	109.5
C(16)-C(19)-H(19C)	109.5
H(19A)-C(19)-H(19C)	109.5
H(19B)-C(19)-H(19C)	109.5

Table SI 14: Anisotropic displacement parameters ($Å^2 x 10^3$) for **4_2**. The anisotropic displacement factor exponent takes the form: $-2p^2[h^2 a^{*2}U^{11} + ... + 2 h k a^* b^* U^{12}]$

	U^{11}	U^{22}	U^{33}	U^{23}	U^{13}	U^{12}
S(1)	26(1)	17(1)	12(1)	-1(1)	-1(1)	7(1)
N(1)	18(1)	14(1)	14(1)	0(1)	0(1)	4(1)
C(1)	21(1)	19(1)	15(1)	1(1)	-1(1)	8(1)
C(2)	45(1)	18(1)	16(1)	3(1)	1(1)	6(1)
C(3)	55(1)	14(1)	24(1)	3(1)	0(1)	5(1)
C(4)	38(1)	16(1)	17(1)	-3(1)	-2(1)	6(1)
C(5)	20(1)	18(1)	15(1)	-2(1)	-1(1)	7(1)
C(6)	25(1)	17(1)	12(1)	-1(1)	2(1)	6(1)
C(7)	30(1)	27(1)	15(1)	1(1)	-3(1)	11(1)
C(8)	26(1)	26(1)	24(1)	-2(1)	9(1)	8(1)
C(9)	46(1)	21(1)	18(1)	-4(1)	4(1)	8(1)
S(11)	25(1)	15(1)	11(1)	-1(1)	2(1)	6(1)
N(11)	17(1)	12(1)	12(1)	1(1)	1(1)	5(1)
C(11)	12(1)	17(1)	13(1)	-1(1)	-1(1)	4(1)
C(12)	22(1)	18(1)	13(1)	3(1)	0(1)	8(1)
C(13)	28(1)	17(1)	20(1)	3(1)	1(1)	10(1)
C(14)	25(1)	17(1)	16(1)	-3(1)	1(1)	10(1)
C(15)	14(1)	17(1)	12(1)	-2(1)	-1(1)	6(1)
C(16)	20(1)	18(1)	11(1)	-1(1)	-1(1)	9(1)
C(17)	21(1)	22(1)	16(1)	2(1)	3(1)	10(1)
C(18)	22(1)	24(1)	14(1)	-2(1)	-3(1)	11(1)
C(19)	33(1)	25(1)	14(1)	-3(1)	1(1)	14(1)

Table SI 15: Hydrogen coordinates (x 10^4) and isotropic displacement parameters ($Å^2$x 10^3) for $\mathbf{4_2}$.

	x	y	z	U(eq)
H(1)	4451(5)	5529(4)	7820(18)	20
H(2)	5011	6448	4856	37
H(3)	5254	6975	6662	44
H(4)	5061	6769	9094	33
H(7A)	4780	5435	9971	39
H(7B)	4802	5576	11636	39
H(7C)	5190	5892	10541	39
H(8A)	3954	5768	10166	42
H(8B)	4043	5502	11414	42
H(8C)	4021	5358	9753	42
H(9A)	5074	6508	11313	48
H(9B)	4664	6187	12332	48
H(9C)	4601	6476	11102	48
H(11)	4241(5)	4689(4)	5638(18)	18
H(12)	3976	3793	8605	22
H(13)	3963	3317	6841	27
H(14)	4064	3530	4390	24
H(17A)	4724	4807	1960	30
H(17B)	4899	4610	3199	30
H(17C)	4732	4950	3614	30
H(18A)	3899	4564	1860	30
H(18B)	3920	4706	3523	30
H(18C)	3567	4211	3020	30
H(19A)	4166	4061	1102	36
H(19B)	3832	3692	2220	36
H(19C)	4351	3851	2263	36

7 Kinetic isotope effect

A kinetic isotope effect of the catalytic dehydrogenation of AB and the isotopologue H_3NBD_3 and D_3NBH_3 was determined. For this purpose, the reactions were done as described in chapter 3 (same concentration (0.15 M) of H_3NBD_3, and D_3NBH_3) but stopped after 10, 20, 30 and 40 min. The conversion of AB, H_3NBD_3 and D_3NBH_3 were determined by measuring an ^{11}B NMR spectrum and comparing the absolute integrals to the line of best fit of the dilution series (see chapter 4.2).

First order kinetics regarding AB, H_3NBD_3 and D_3NBH_3 were assumed. For the determination of rate constants, every data point was determined twice, and the averaged value was used (Table SI 16).

Table SI 16: Conversion of AB and H_3NBD_3 for the determination of the kinetic isotope effect.

time [min]	absolute integral of AB	concentration of AB in reaction solution [M]	absolute integral of H_3NBD_3	concentration of H_3NBD_3 in reaction solution [M]	absolute integral of H_3NBD_3	concentration of D_3NBH_3 in reaction solution [M]
10	247687	0.137	267673	0.149	288217	0.160
10	228742	0.127	264835	0.147	313003	0.174
20	173137	0.095	212905	0.118	248413	0.138
20	175918	0.097	197115	0.109	259204	0.144
30	131539	0.072	135995	0.074	221295	0.123
30	115403	0.063	159504	0.088	225827	0.125
40	105304	0.057	141777	0.078	199162	0.110
40	86243	0.046	109303	0.059	206485	0.114

The values were plotted according to the following formula (Figure SI 18, 19, and 20):

$$\ln([A]) = -\mathrm{k}\,\mathrm{t} + \ln([A_0])$$

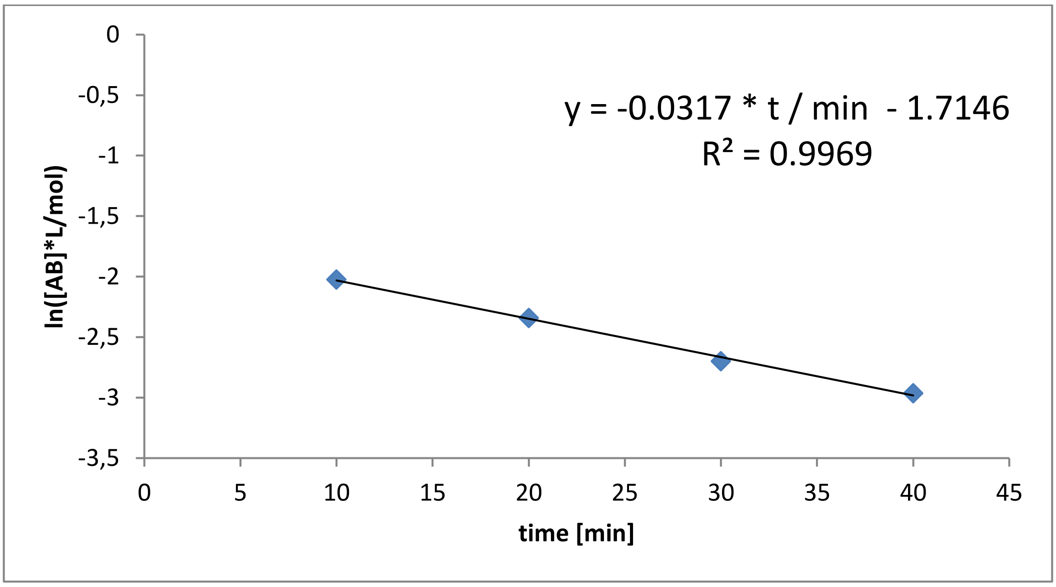

Figure SI 18: Kinetic plot of the catalytic dehydrogenation of AB.

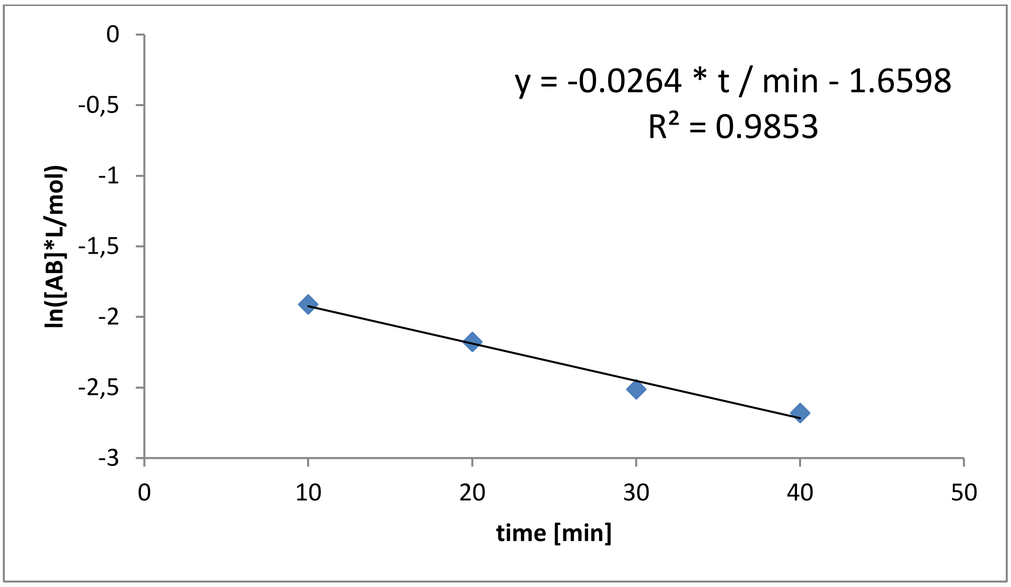

Figure SI 19: Kinetic plot of the catalytic dehydrogenation of H_3NBD_3.

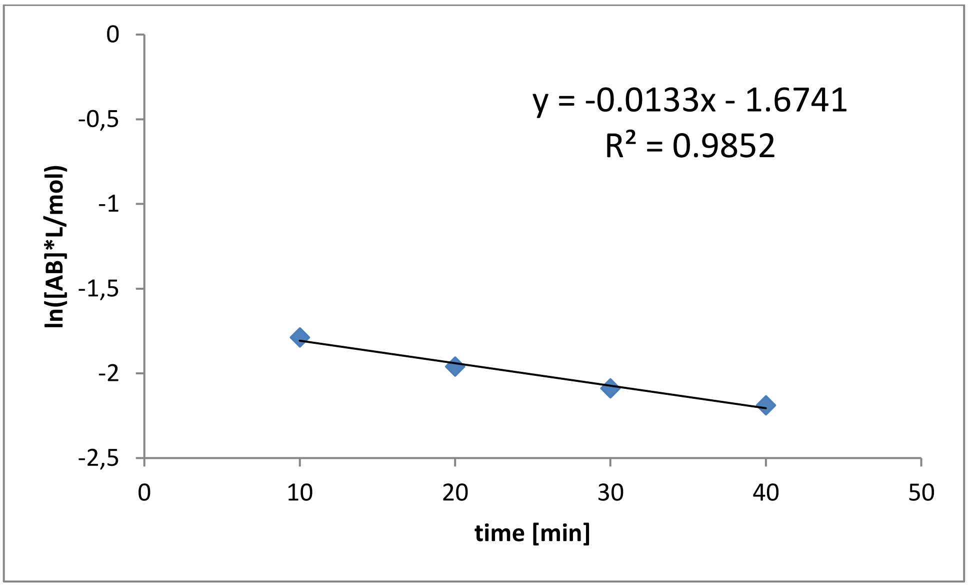

Figure SI 20: Kinetic plot of the catalytic dehydrogenation of D_3NBH_3.

The rate constants are:

AB: $k = 0.0317\ min^{-1} \pm 0.0013\ min^{-1}$

H_3NBD_3: $k = 0.0264\ min^{-1} \pm 0.0023\ min^{-1}$

D_3NBH_3: $k = 0.0133\ min^{-1} \pm 0.0012\ min^{-1}$

The kinetic isotope effects are:

KIE (H_3NBD_3) = 1.20 ± 0.15

KIE (D_3NBH_3) = 2.38 ± 0.30

8 Deuterium incooperation in 6-*tert*-butyl-2-thiopyridone

Inside the glove box 6-*tert*-butyl-2-thiopyridone (10.2 mg, 0.06 mmol) and D_3NBH_3 (2.0 mg, 0.06 mmol) were dissolved in 0.4 mL THF-d_8 in a NMR tube with J-Young valve. The tube was closed, taken outside the glovebox and after 2 h at room temperature ^1H, ^{11}B and ^2H NMR spectra were recorded. The ^{11}B NMR spectra just shows unreacted D_3N-BH_3 while the ^1H and ^2H spectra show a H/D exchange of about 10% (Figure SI 21 and 22).

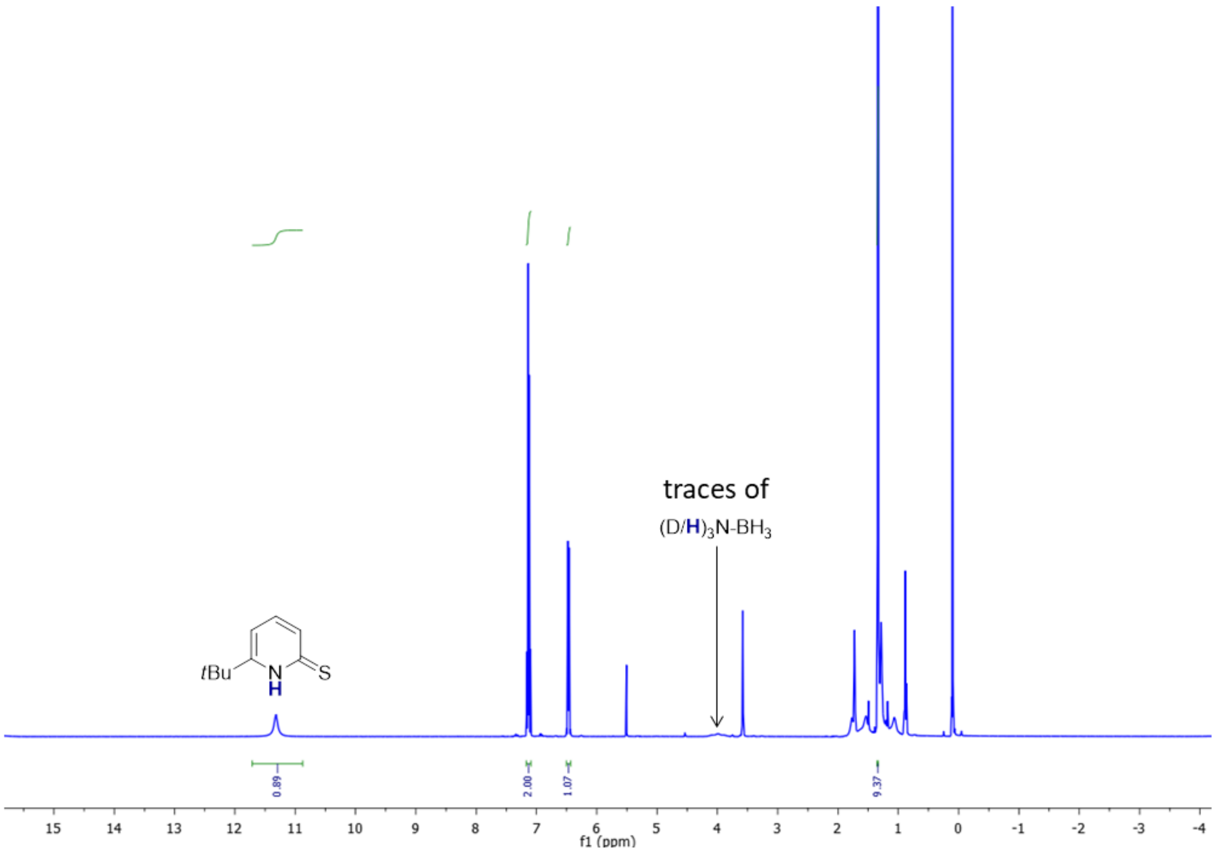

Figure SI 21: ^1H NMR spectrum of the stoichiometric reaction of 6-*tert*-butyl-2-thiopyridone with D_3N-BH_3 after 2 hours at room temperature (400 MHz, THF-d_8). Formal charges are omitted for clarity.

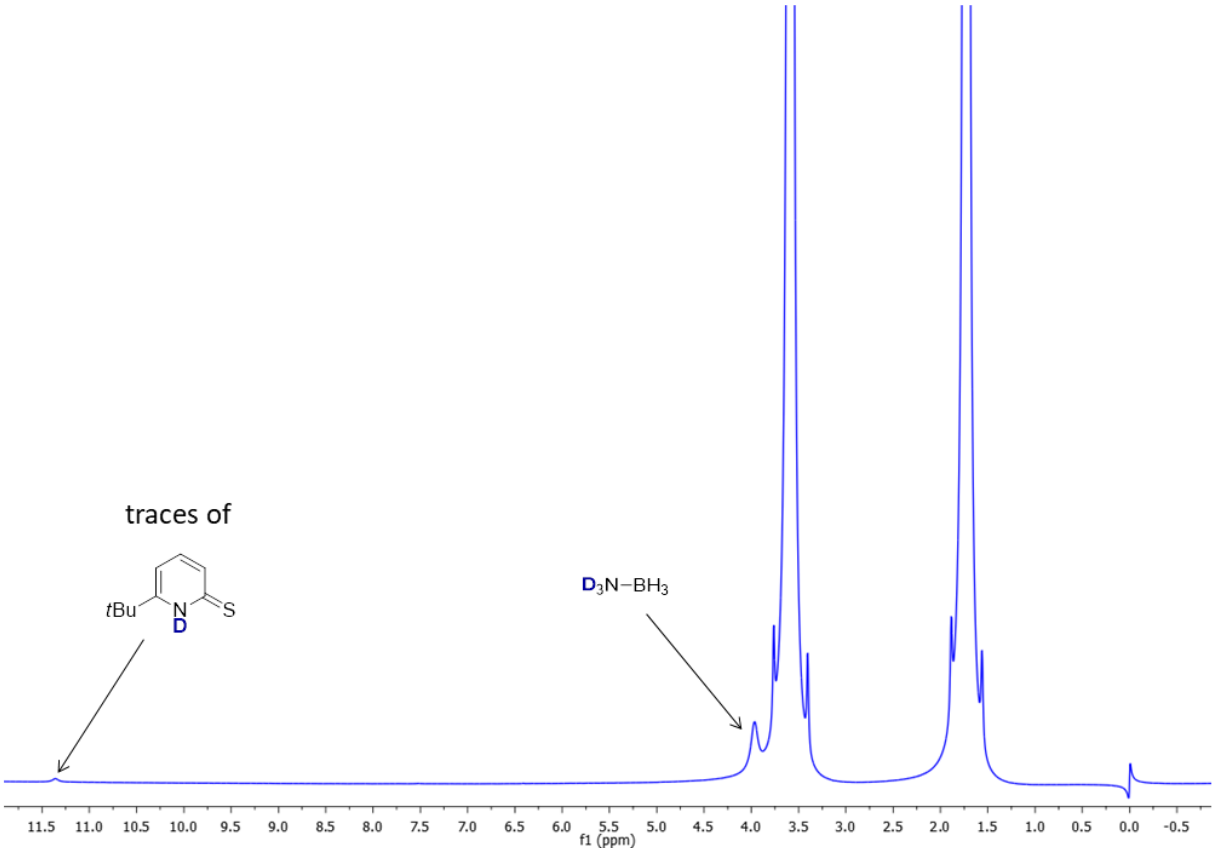

Figure SI 22: ^{2}H NMR spectrum of the stoichiometric reaction of 6-*tert*-butyl-2-thiopyridone with D_3N-BH_3 after 2 hours at room temperature (61 MHz, THF-d_8). Formal charges are omitted for clarity.

Afterwards, the reaction solution was heated to 80 °C for two hours. After the reaction time the corresponding ^{1}H, ^{2}H and ^{11}B NMR spectra show that nearly all of D_3NBH_3 was converted to borazine while about 90% of the hydrogen bond to the nitrogen in 6-*tert*-butyl-2-thiopyridone was replaced by deuterium (Figure SI 22, 23 and 24). Additionally, intermediate **5** is present in low concentrations. The ^{2}H NMR spectrum shows a signal at 5.40 ppm which we attentively assigned to the ND_3 moiety of intermediate **5**.

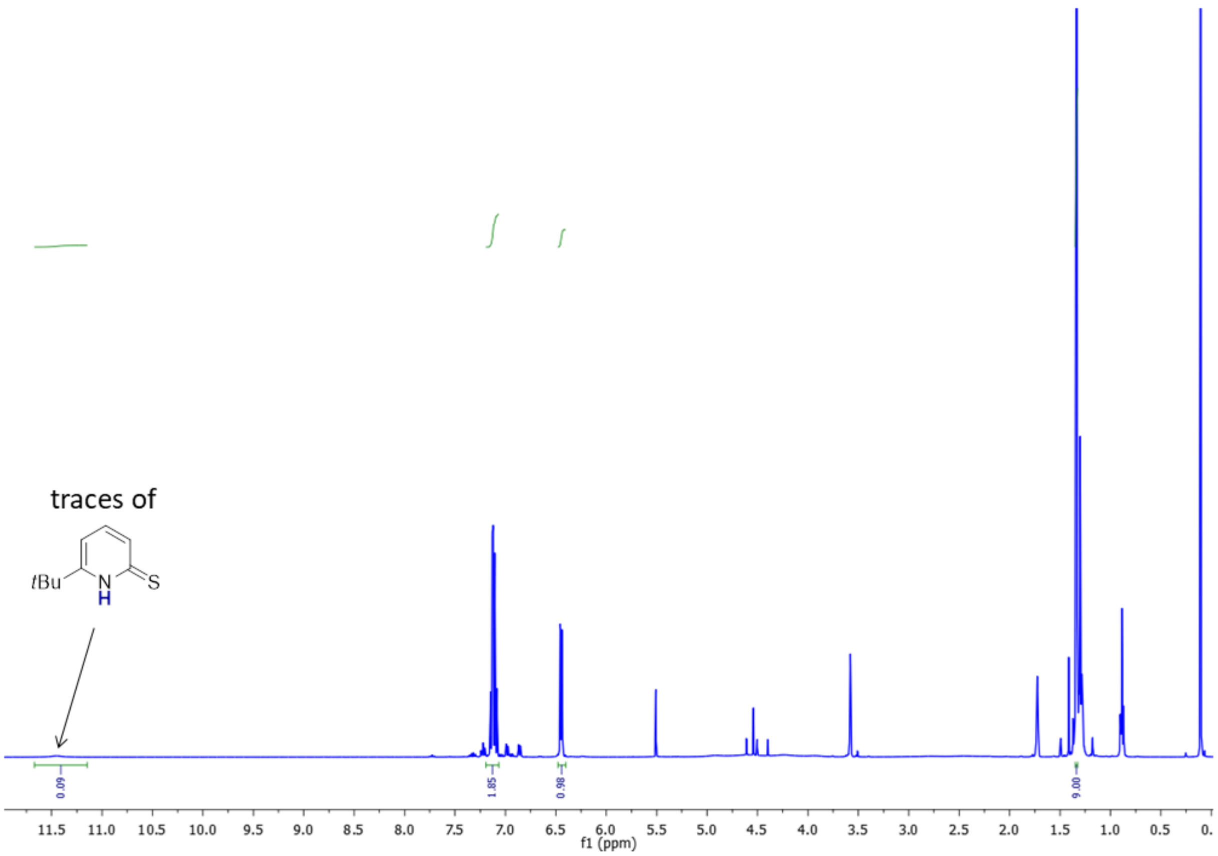

Figure SI 23: ^{1}H NMR spectrum of the stoichiometric reaction of 6-*tert*-butyl-2-thiopyridone with $D_3N\text{-}BH_3$ after heating for 2 hours at 80 °C (400 MHz, THF-d_8).

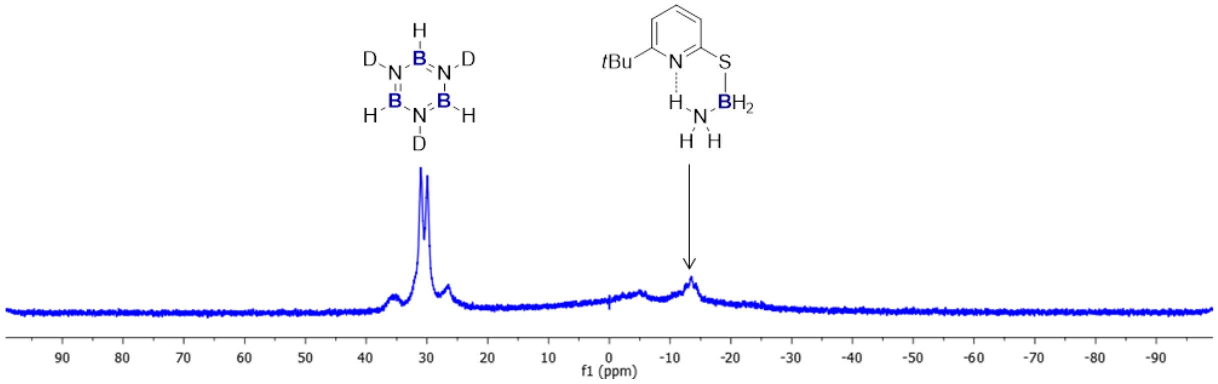

Figure SI 24: ^{11}B NMR spectrum of the stoichiometric reaction of 6-*tert*-butyl-2-thiopyridone with D_3NBH_3 after heating for 2 hours at 80 °C (128 MHz, THF-d_8). Formal charges are omitted for clarity.

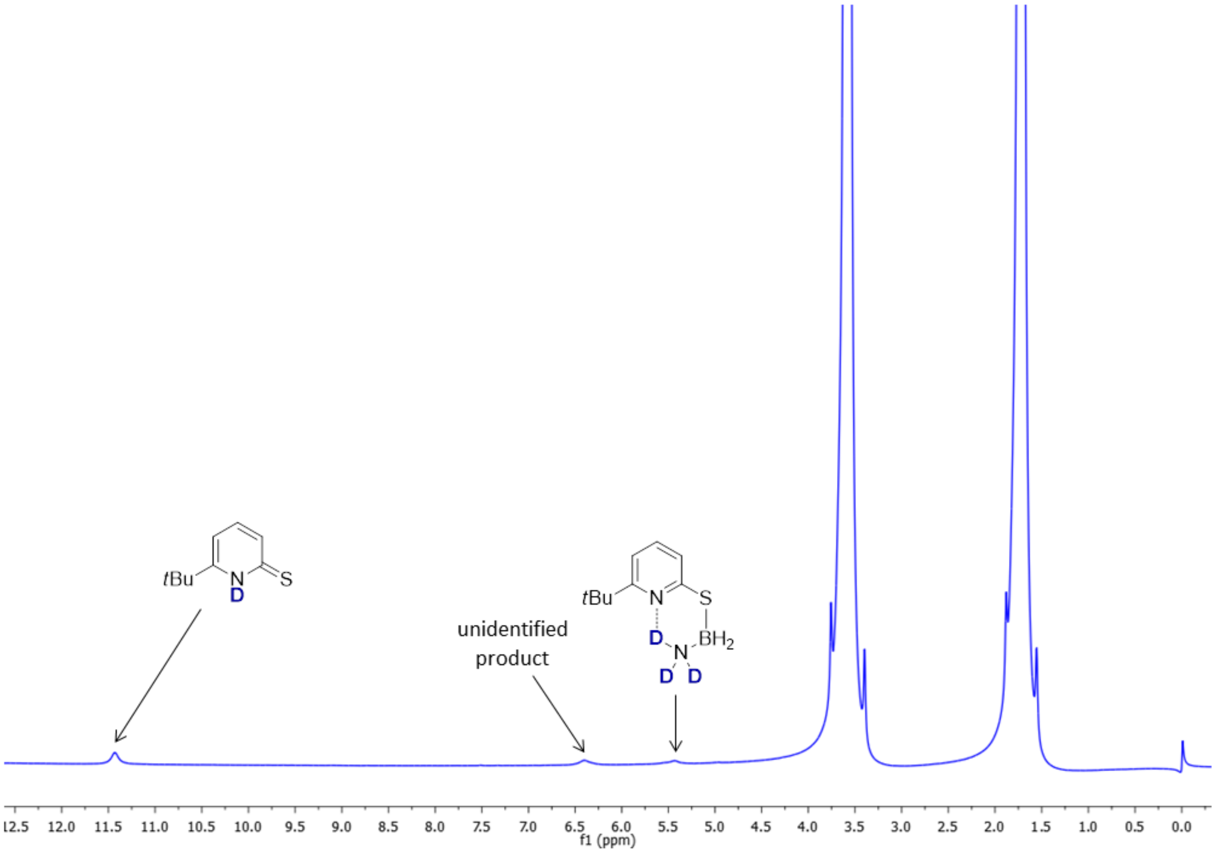

Figure SI 25: ^{2}H NMR spectrum of the stoichiometric reaction of 6-*tert*-butyl-2-thiopyridone with D_3N-BH_3 after heating for 2 at 80 °C (61 MHz, THF-d_8). Formal charges are omitted for clarity.

9 Catalytic dehydrogenation of DMAB

The experiments of the catalytic dehydrogenation of DMAB was done as described in chapter 3 only that 4.5 mL of toluene and 0.5 mL of the 0.15 mM stock solution of 6-*tert*-butyl-2-thiopyridone in THF was used and the reaction flask was heated to 120 °C for 4 hours. The ^{11}B NMR spectrum shows the nearly complete dehydrogenation product of DMAB with traces of unreacted DMAB

After the reaction time, 18.2 mL of gas was produced corresponding to 1 eq. of H_2. A blind experiment shows only traces of the dehydrogenation product of DMAB in the ^{11}B NMR spectrum.

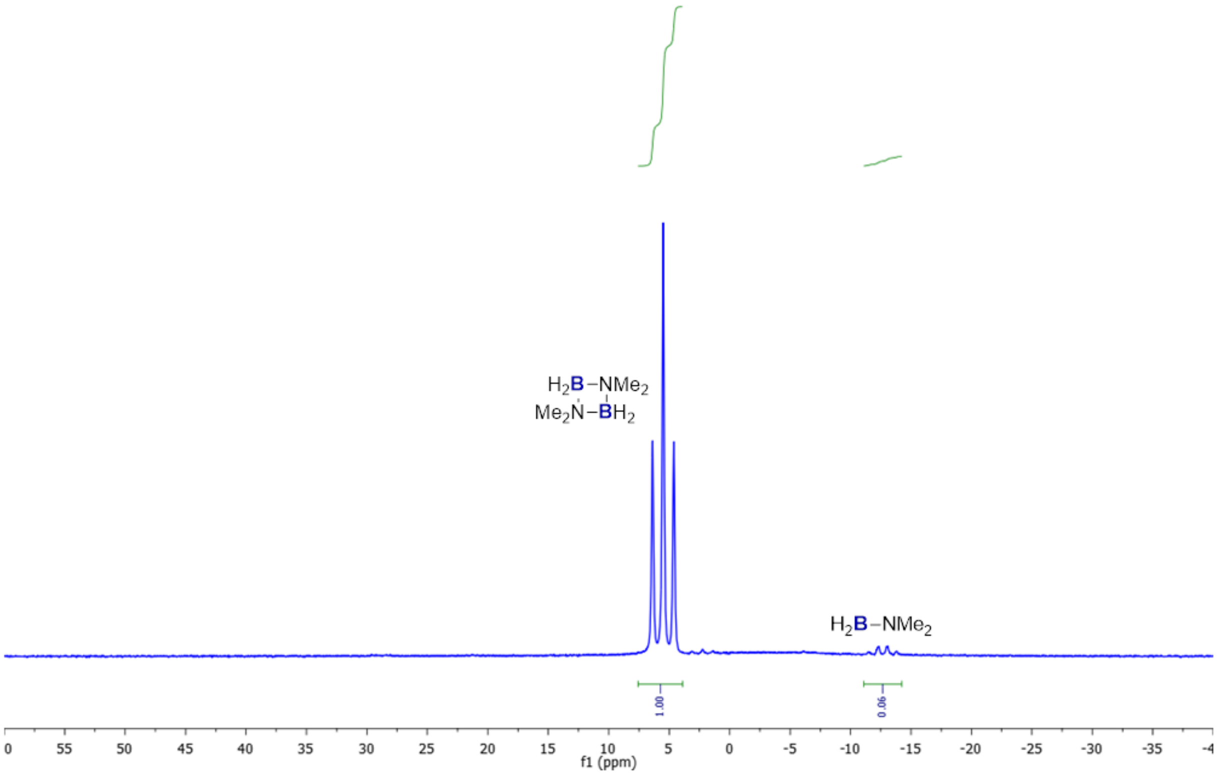

Figure SI 26: ^{11}B NMR spectrum of the catalytic dehydrogenation of DMAB using 6-*tert*-butyl-2-thiopyridone after 4 h at 80 °C. (128 MHz, toluene with benzene-d_6 glass capillary). Formal charges are omitted for clarity.

10 Characterization of intermediate 5 and a corresponding fragment ion by mass spectrometry

We could characterize **5** with high mass accuracy by Nano-ESI-MS (Figure SI 27). However, the corresponding signal has a low intensity, presumably due to the irreversible loss of NH_2BH_2.

Recalibrated to m/z 168.0841

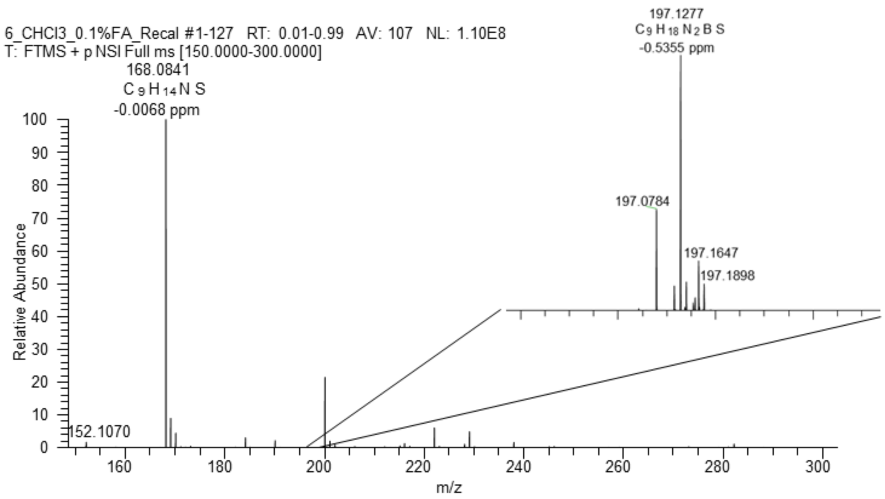

Signal	Intensity ration (theo.)	Intensity ratio (exp.)	Δppm
$C_9H_{18}N_2BS^+$	100	100	-0.539
$C_9H_{18}N_2{}^{10}BS^+$	25	14	-0.958
$C_8{}^{13}CH_{18}N_2BS^+$	10	3	-0.611

Figure SI 27: Nano-ESI-MS spectrum of a reaction solution with intermediate **5.**

Additionally, we were able to characterize a fragment ion of **5** by ESI-TOF mass spectrometry.

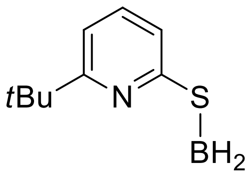

HR-ESI-MS found (M+H$^+$), 180.1024, $C_9H_{14}BNS$ predicted (M+H$^+$), 180.1013

11 IR spectrum of 6-*tert*-butyl-2-thiopyridone in THF

To show that in a THF solution 6-*tert*-butyl-2-thiopyridone exists in its thiolactam tautomeric form a 1 M solution of 6-*tert*-butyl-2-thiopyridone in THF was prepared. The IR spectrum of this solution was recorded as a film between two KBr windows. Additionally, THF as a pure solvent was measured as a reference. Figure SI 28 shows the corresponding IR spectra. The THF reference is shown in red.

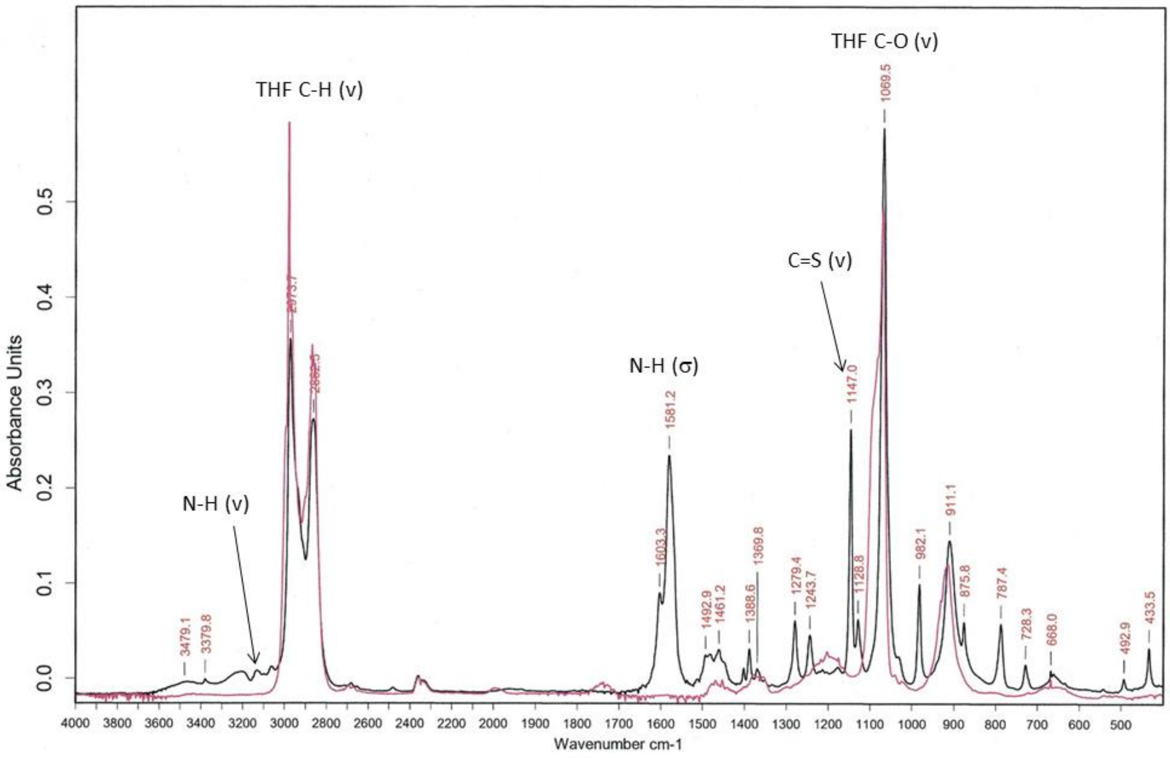

Figure SI 28: IR spectrum of 6-*tert*-butyl-2-thiopyridone in THF (1 M) (black) and the THF reference spectrum (red).

12 Pathway without a second molecule of AB

Without the second AB molecule, the transition state collapses to a transition state where transfer of the NH proton to the mercaptopyridine accompanies protonation of the BH bond. This transition state requires an activation energy of 31.7 kcal/mol.

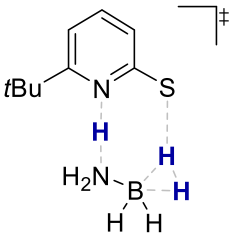

TS'$_{\mathbf{4SH.AB/4+AB+H2}}$
ΔG=31.7

Figure SI 29: Gibbs free energies of the dehydrogenation of AB catalyzed by **4SH** without stabilization by a second molecule AB computed at TightPNO-DLPNO-CCSD(T)/def2-QZVPP//PBE0-D3(BJ)/def2-TZVP. The Gibbs free energy is given with respect to $\mathbf{4_2}$ and AB. Solvent effects were implicitly considered using the SMD model for THF. Formal charges on nitrogen and boron in the transition structure are omitted for clarity.

13 Formation of Cyclotriborazane

The formation of cyclotriborazane is exergonic, but the trimerization has a high kinetic barrier of 31.8 kcal/mol. Note that due to the change in the molecularity from three to one an overestimation of the entropic penalty by the computations might be expected.

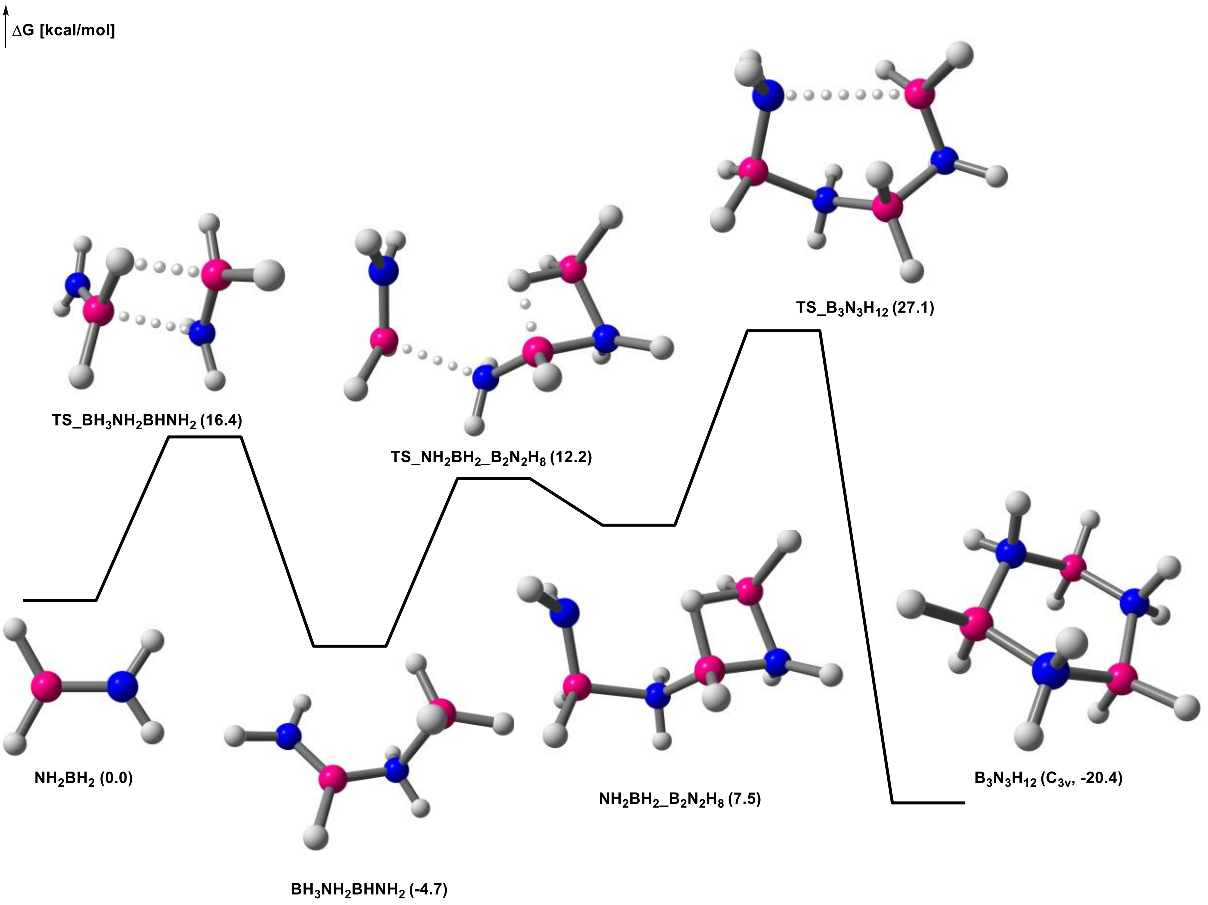

Figure SI 30: Gibbs free energies of trimerization of NH2BH2 computed at TightPNO-DLPNO-CCSD(T)/def2-QZVPP//PBE0-D3(BJ)/def2-TZVP. The Gibbs free energy is given with respect to three molecules of AB. Solvent effects were implicitly considered using the SMD model for THF. Formal charges on nitrogen and boron in the transition structure are omitted for clarity.

14 Comparison of the computed PES of the dehydrogenation of AB catalyzed by 3 or 4 as catalyst

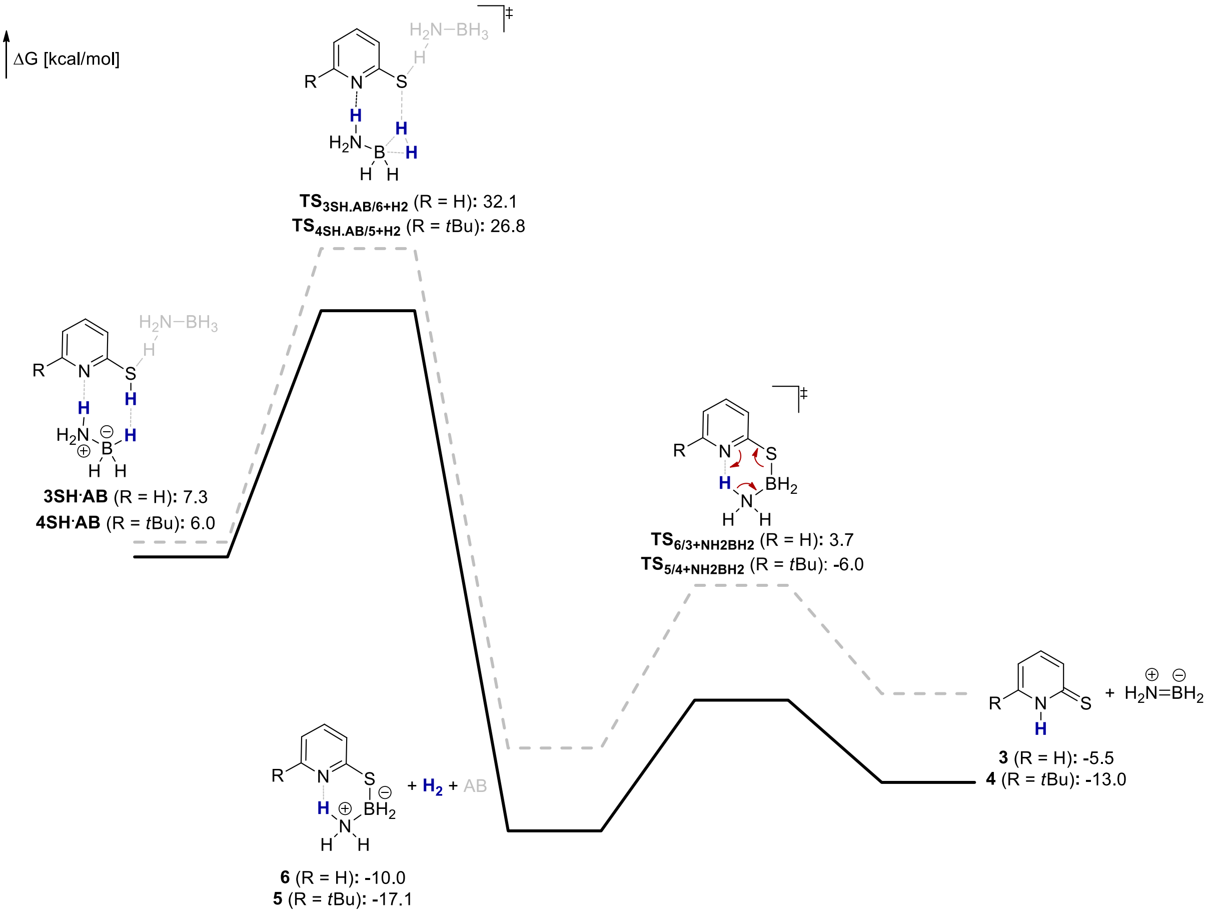

Figure SI 31: Gibbs free energies of the dehydrogenation of AB catalyzed by **3** or **4** computed at TightPNO-DLPNO-CCSD(T)/def2-QZVPP//PBE0-D3(BJ)/def2-TZVP. Solvent effects were implicitly considered using the SMD model for THF. All Gibbs free energies are given with respect to $\mathbf{4_2}$ and $\mathbf{AB_2}$. Formal charges on nitrogen and boron in the transition structure are omitted for clarity.

15 Dimerization of 4

The computed free energies predict the dimerization of **4** to be endergonic by 5.7 kcal/mol (Scheme SI 3).

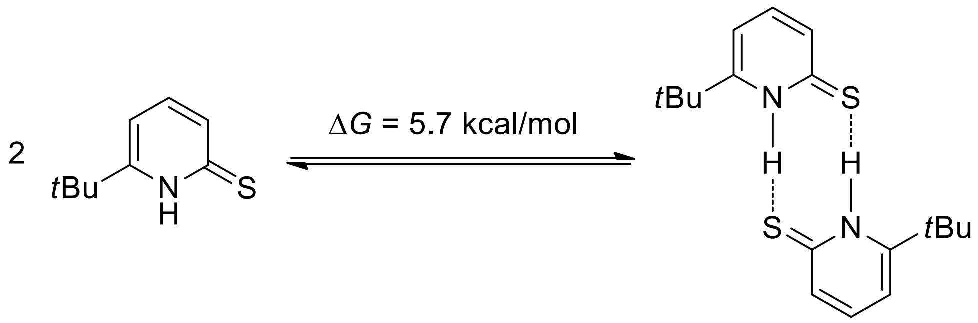

Scheme SI 3: Gibbs free energies of the dimerization of **4** computed at TightPNO-DLPNO-CCSD(T)/def2-QZVPP//PBE0-D3(BJ)/def2-TZVP. Solvent effects were implicitly considered using the SMD model for THF. Formal charges on nitrogen and boron in the transition structure are omitted for clarity.

However, it is evident from ^{1}H NMR spectra that **4** in THF is present as a dimer at room temperature. To elucidate the origin of this discrepancy, the thermochemical parameters of the dimerization of **4** were investigated by VT ^{1}H NMR.

The thermodynamic values of the dimerization of **4** to **4_2** were estimated by using a method established by Limbach *et al.*[11] Three different concentrations of **4** (0.05, 0.005, and 0.0025 M) in dry THF-d_8 in an NMR tube with J Young valve were prepared inside the glovebox. ^{1}H NMR spectra of all three samples were recorded at different temperatures ranging from 333 to 218 K and the chemical shift of the N-H signal was observed (Table SI 5).

Table SI 5: Temperature-dependent N-H shift of the 0.05, 0.005, and 0.0025 M solutions of 6-*tert*-butyl-2-thiopyridone in THF-d_8.

	**0.05 M**	**0.005 M**	**0.0025 M**
**T [K]**	**N-H shift [ppm]**		
**333**	-	11.0988	11.0968
**323**	-	11.1908	11.1891
**313**	-	11.2853	11.2791
**303**	-	11.3795	11.3732
**293**	11.4879	11.4723	11.4629
**283**	11.5808	11.5659	-
**273**	11.6690	11.6589	-
**263**	11.7592	11.7497	-
**253**	11.8460	11.8355	-
**243**	11.9247	11.9171	-
**233**	11.9986	11.9939	-
**223**	12.0675	12.0629	-
**218**	12.1013	12.0974	-

The observed chemical shift σ_{obs} can be calculated using the following formulas:

$$\delta_{obs} = \frac{c[4]}{c_{tot}}\delta_4 + \frac{2\,c[4_2]}{c_{tot}}\delta_{4_2}$$

$$K = \exp(-\frac{\Delta H - T\Delta S}{RT}) = \frac{c\,[4_2]}{c\,[4]^2}$$

$$c_{tot} = c[4] + 2\,c[4_2]$$

The plots of the temperature-dependent N-H shift were subject to an individual sigmoidal fit (Figure SI 32, 33 and 34).

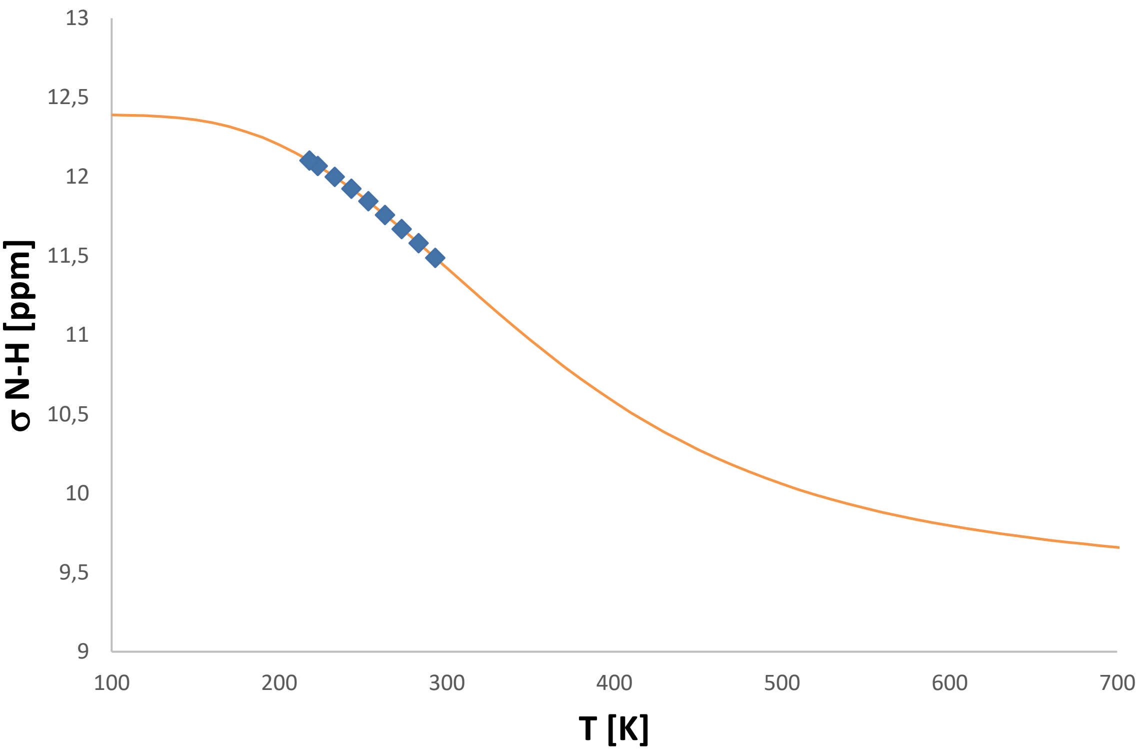

Figure SI 32: Sigmoidal fit of the temperature-dependent N-H shift of the 0.05 M solution of 6-*tert*-butyl-2-thiopyridone in THF-d_8.

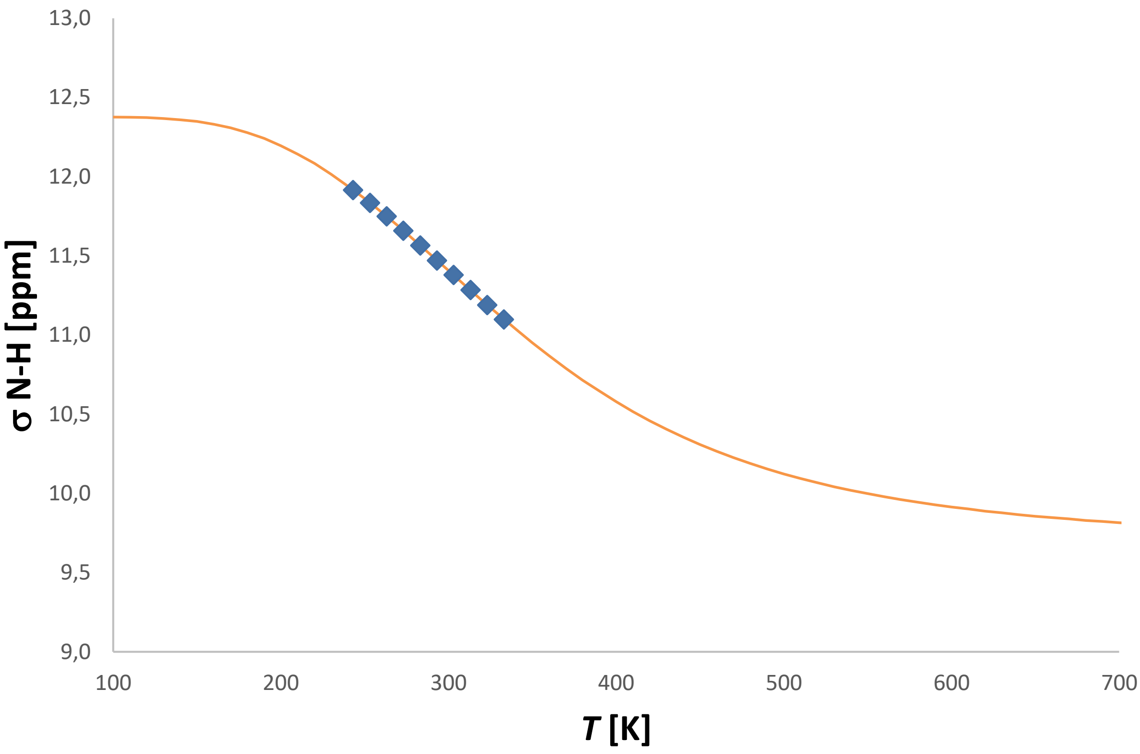

Figure SI 33: Sigmoidal fit of the temperature-dependent N-H shift of the 0.005 M solution of 6-*tert*-butyl-2-thiopyridone in THF-d_8.

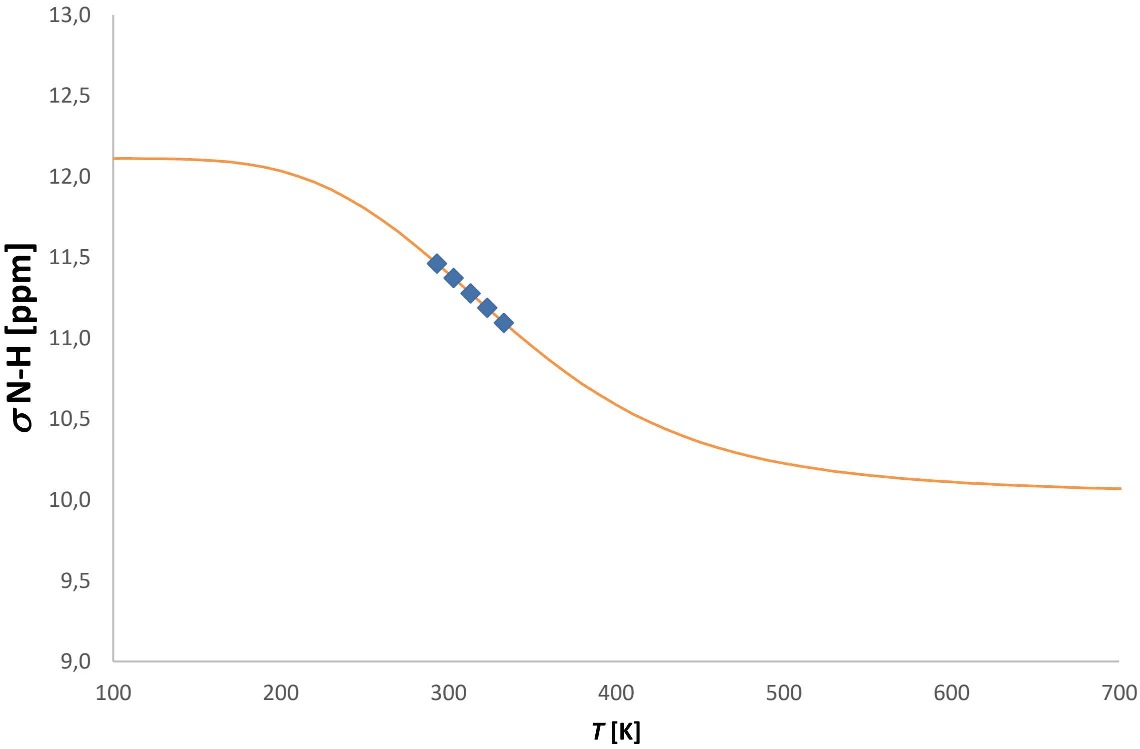

Figure SI 34: Sigmoidal fit of the temperature-dependent N-H shift of the 0.0025 M solution of 6-*tert*-butyl-2-thiopyridone in THF-d_8.

We were not able to reach the saturation region of the chemical shift. However, the thermodynamic values of the dimerization were estimated by using the average of all three individually fitted sigmoidal curves as shown in Scheme SI 4.

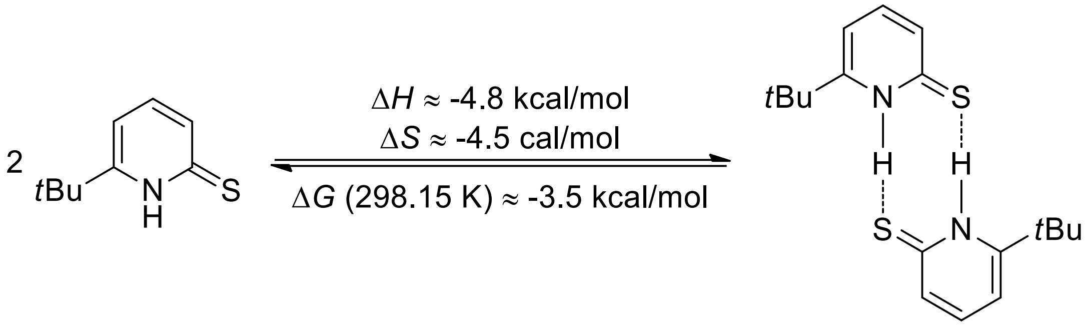

Scheme SI 4: The estimated thermodynamic values of the dimerization of 6-*tert*-butyl-2-thiopyridone in THF.

The estimated enthalpy is in excellent agreement with the computed value:

SMD(THF)-TightPNO-DLPNO-CCSD(T)/def2-QZVPP//PBE0-D3(BJ)/def2-TZVP: ΔH = -4.5 kcal/mol

Exp.: ΔH = -4.8 kcal/mol

This comparision shows that the discrepancy between experimental and computed ΔGs originates from a overestimation of entropic penalty.[12]

16 Additional NMR spectra

16.1 ^{11}B NMR spectra of catalysis experiments

The assignment of the dehydrogenation products of AB was done based on previous literature from *Aldridge et al.* and integral ratios.[5b]

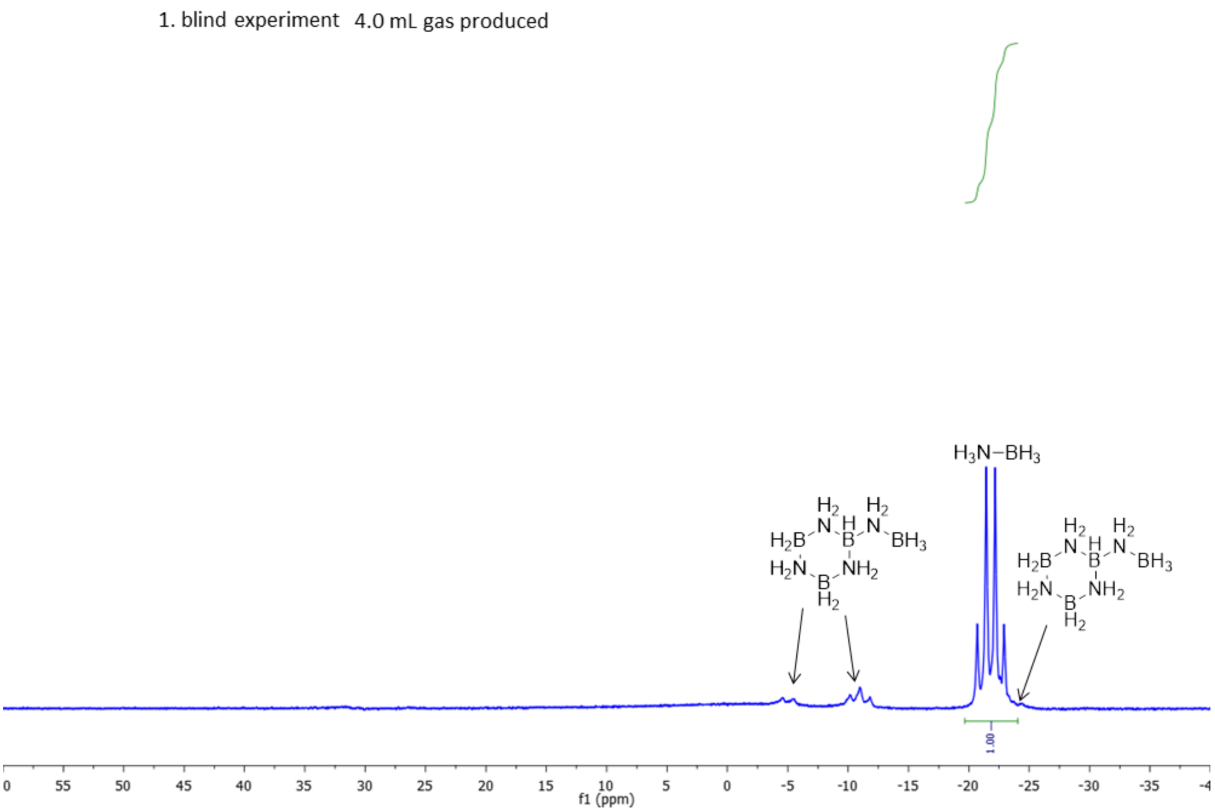

Figure SI 35: ^{11}B NMR spectrum of the first dehydrogenation blind experiment of AB (128 MHz, THF with benzene-d_6 glass capillary). Formal charges are omitted for clarity.

2. blind experiment 5.0 mL gas produced

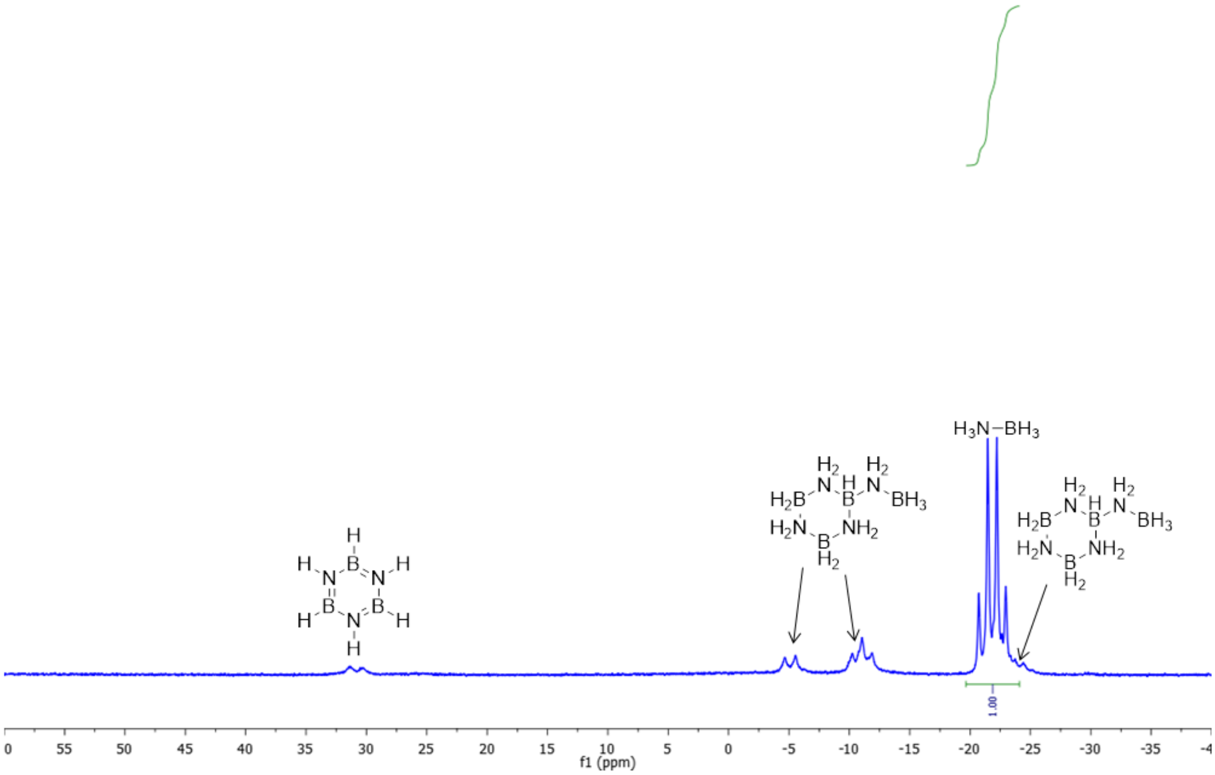

Figure SI 36: ^{11}B NMR spectrum of the second dehydrogenation blind experiment of AB (128 MHz, THF with benzene-d_6 glass capillary). Formal charges are omitted for clarity.

3. blind experiment 3.0 mL gas produced

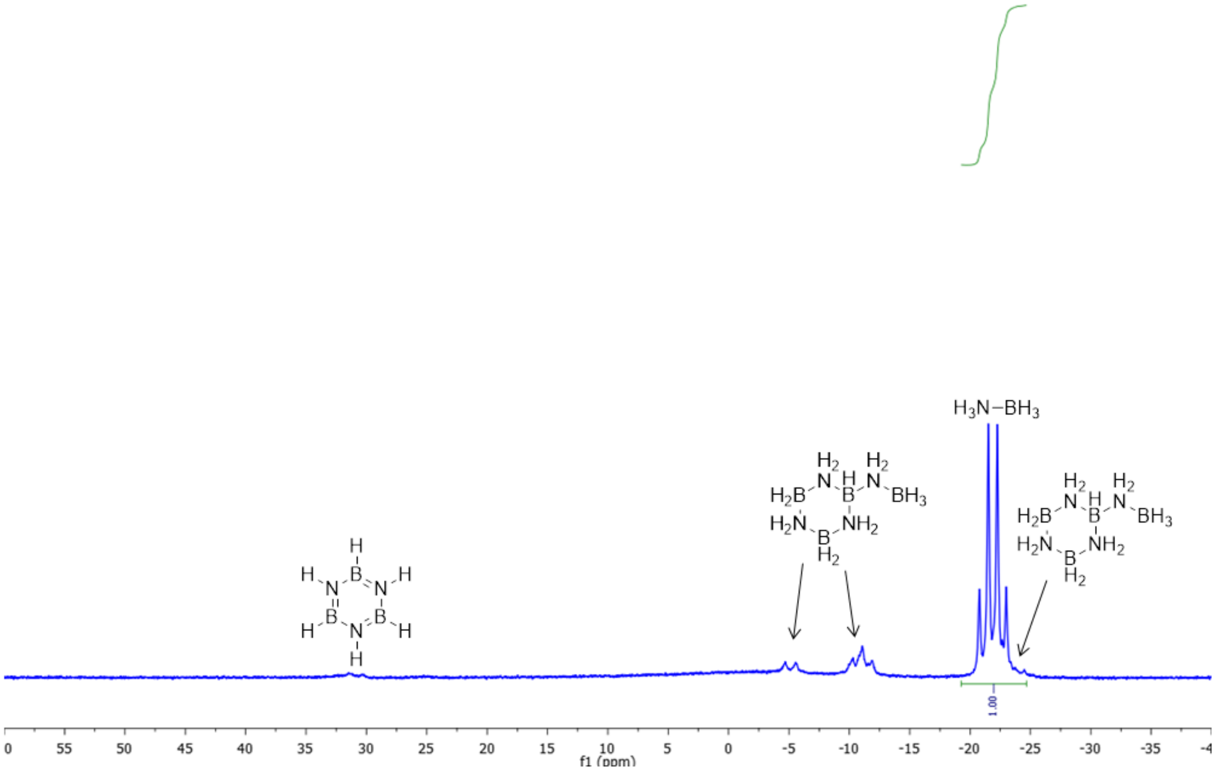

Figure SI 37: ^{11}B NMR spectrum of the third dehydrogenation blind experiment of AB (128 MHz, THF with benzene-d_6 glass capillary). Formal charges are omitted for clarity.

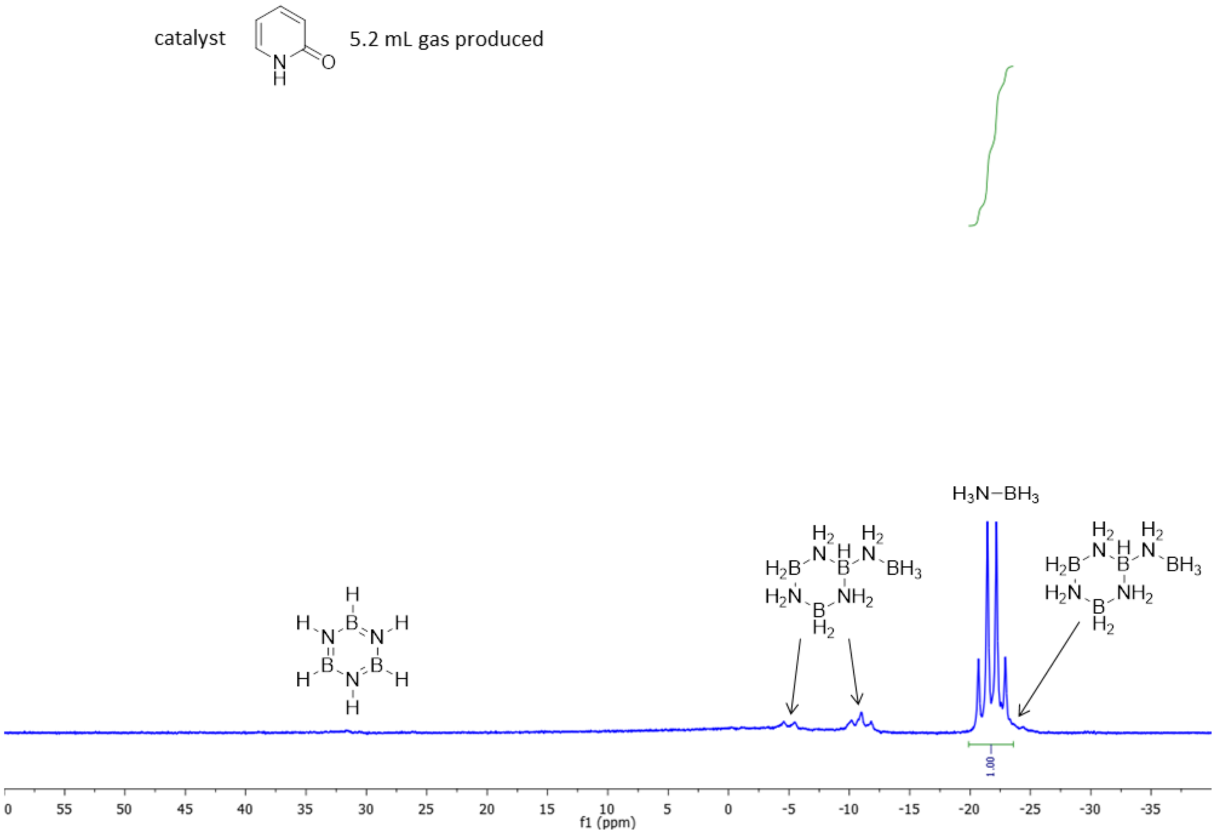

Figure SI 38: ^{11}B NMR spectrum of the first dehydrogenation experiment of AB with 2-pyridone as catalyst (128 MHz, THF with benzene-$d_6$glass capillary). Formal charges are omitted for clarity.

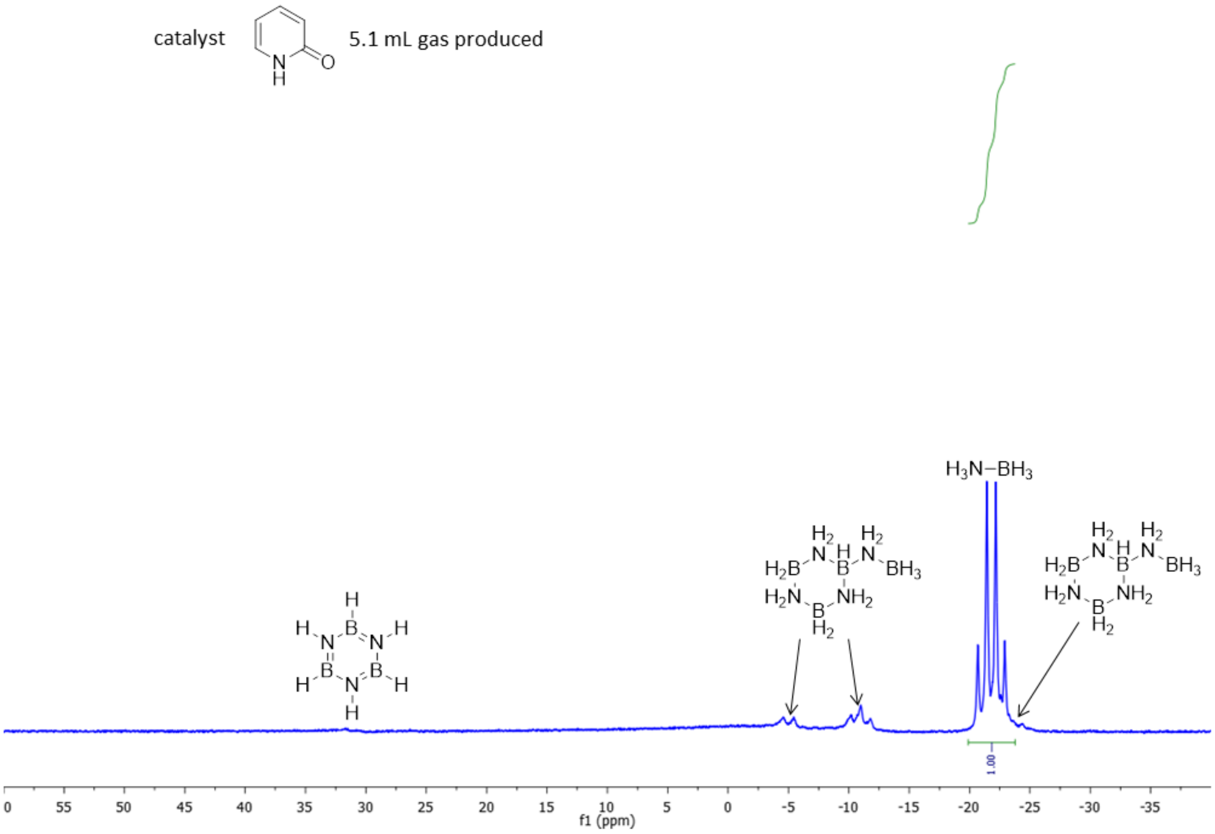

Figure SI 39: ^{11}B NMR spectrum of the second dehydrogenation experiment of AB with 2-pyridone as catalyst (128 MHz, THF with benzene-d_6 glass capillary). Formal charges are omitted for clarity.

catalyst 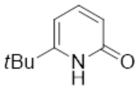 15.9 mL gas produced

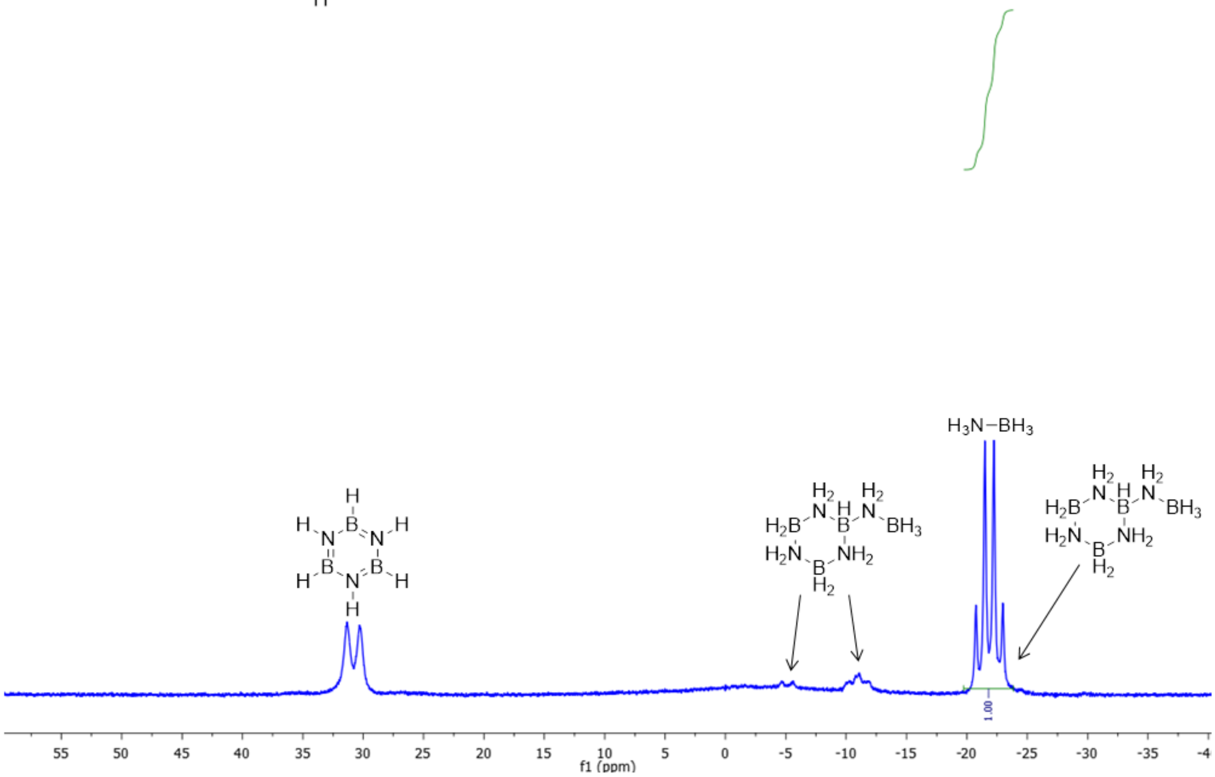

Figure SI 40: ^{11}B NMR spectrum of the first dehydrogenation experiment of AB with 6-*tert*-butyl-2-pyridone as catalyst (128 MHz, THF with benzene-d_6 glass capillary). Formal charges are omitted for clarity.

catalyst 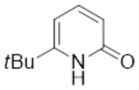 14.5 mL gas produced

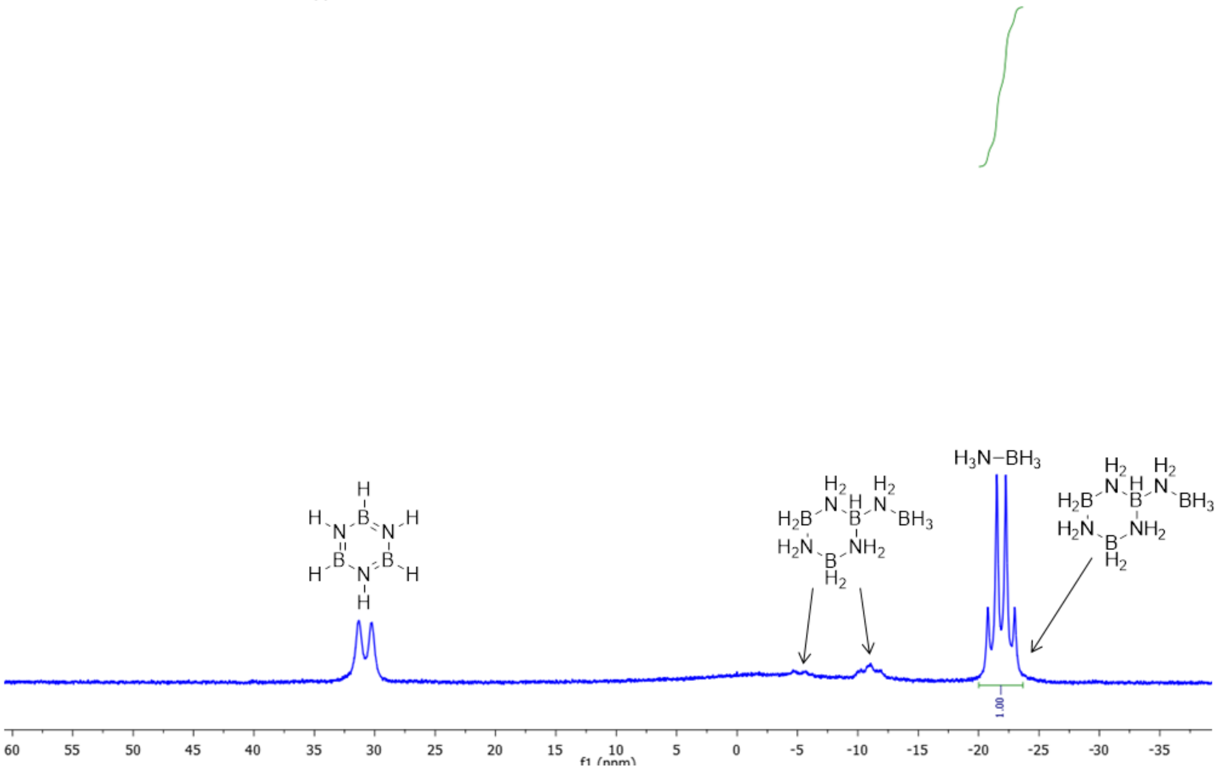

Figure SI 41: ^{11}B NMR spectrum of the second dehydrogenation experiment of AB with 6-*tert*-butyl-2-pyridone as catalyst (128 MHz, THF with benzene-d_6 glass capillary). Formal charges are omitted for clarity.

catalyst 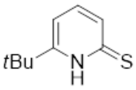 35.9 mL gas produced

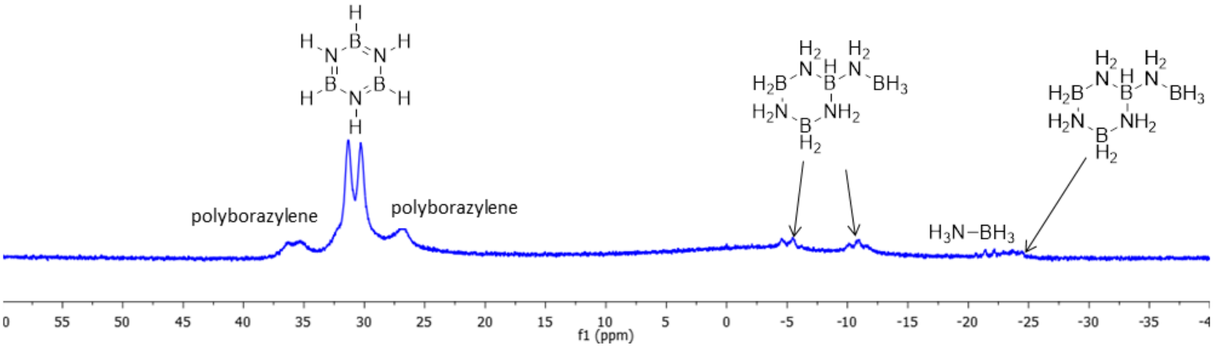

Figure SI 42: ^{11}B NMR spectrum of the first dehydrogenation experiment of AB with 6-*tert*-butyl-2-thiopyridone as catalyst (128 MHz, THF with benzene-d_6 glass capillary). Formal charges are omitted for clarity.

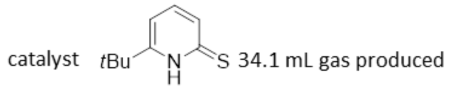

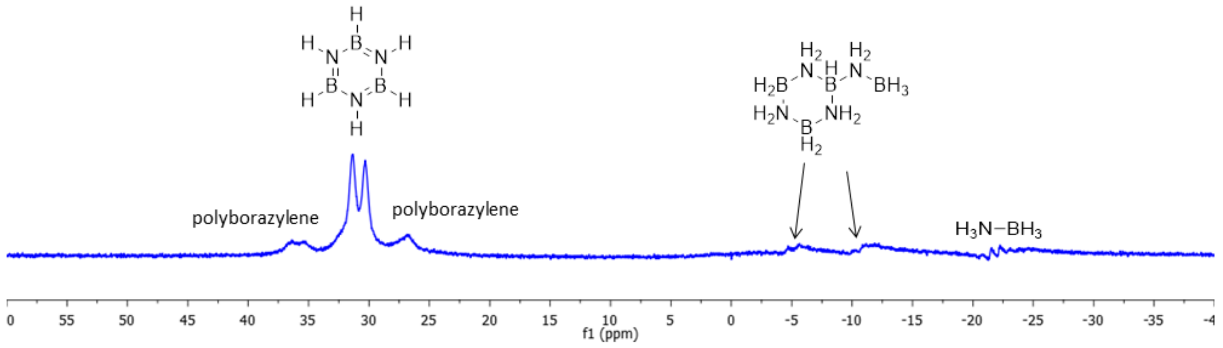

Figure SI 43: ^{11}B NMR spectrum of the second dehydrogenation experiment of AB with 6-*tert*-butyl-2-thiopyridone as catalyst (128 MHz, THF with benzene-d_6 glass capillary). Formal charges are omitted for clarity.

catalyst 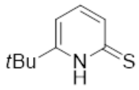 38.2 mL gas produced

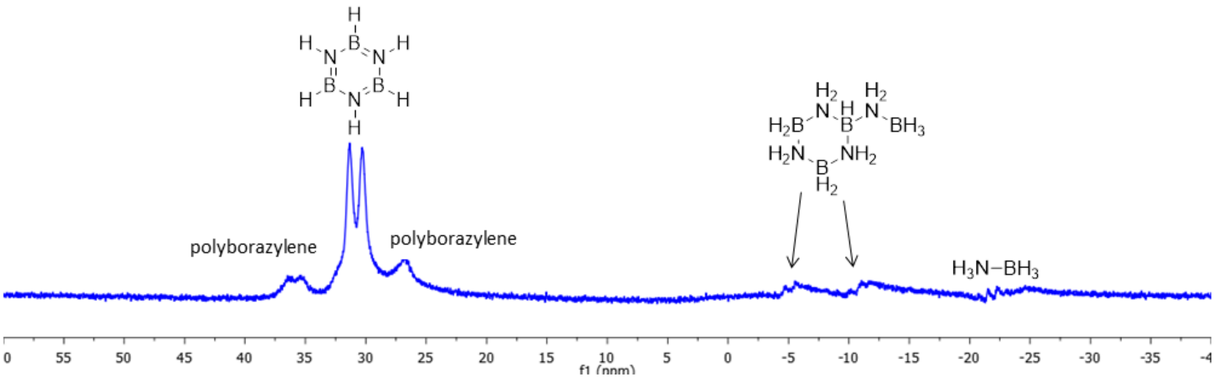

Figure SI 44: ^{11}B NMR spectrum of the third dehydrogenation experiment of AB with 6-*tert*-butyl-2-thiopyridone as catalyst (128 MHz, THF with benzene-d_6 glass capillary). Formal charges are omitted for clarity.

catalyst 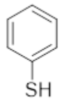 13.5 mL gas produced

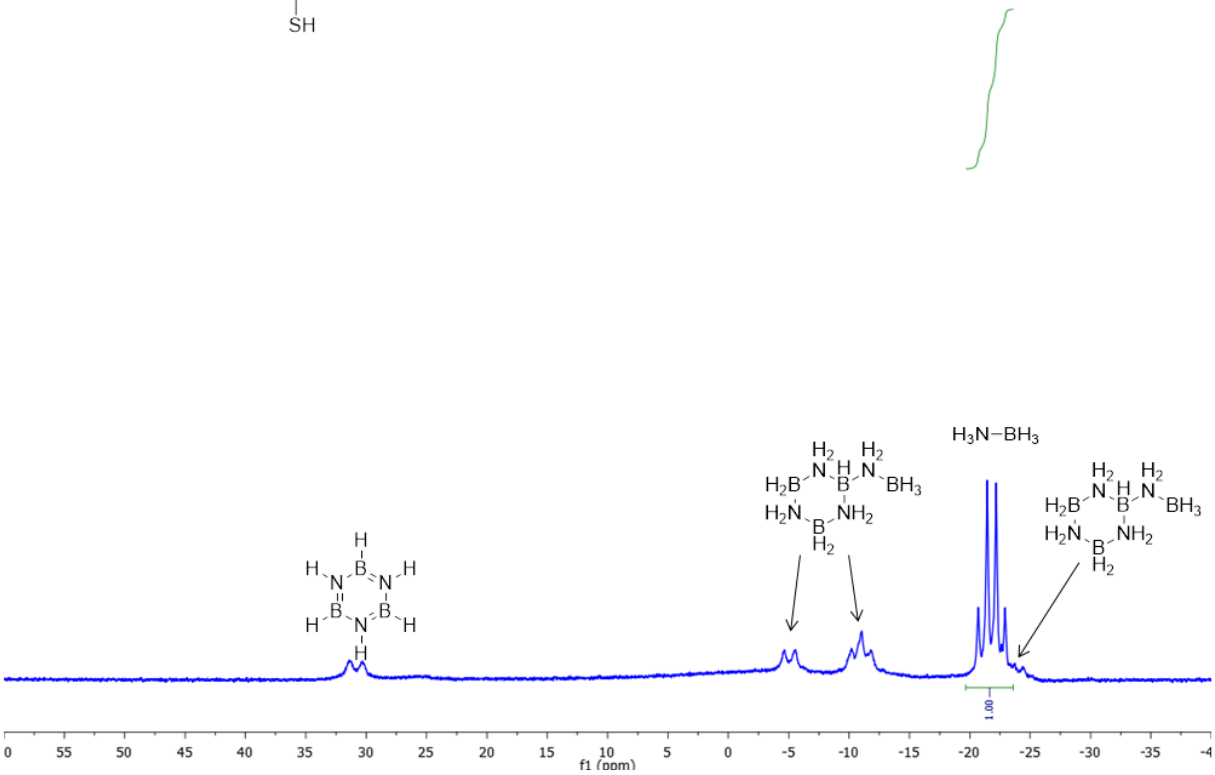

Figure SI 45: ^{11}B NMR spectrum of the first dehydrogenation experiment of AB with thiophenol as catalyst (128 MHz, THF with benzene-d_6 glass capillary). Formal charges are omitted for clarity.

catalyst 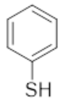 14.0 mL gas produced

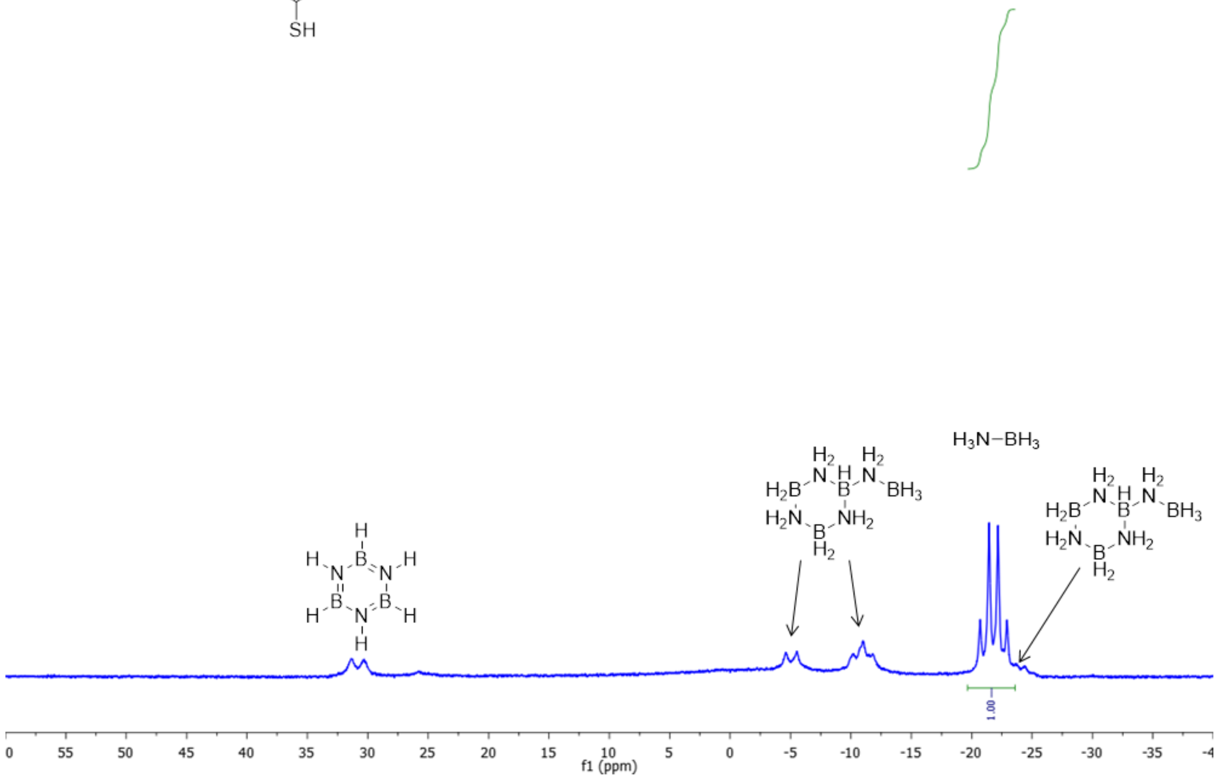

Figure SI 46 ^{11}B NMR spectrum of the second dehydrogenation experiment of AB with thiophenol as catalyst (128 MHz, THF with benzene-d_6 glass capillary). Formal charges are omitted for clarity.

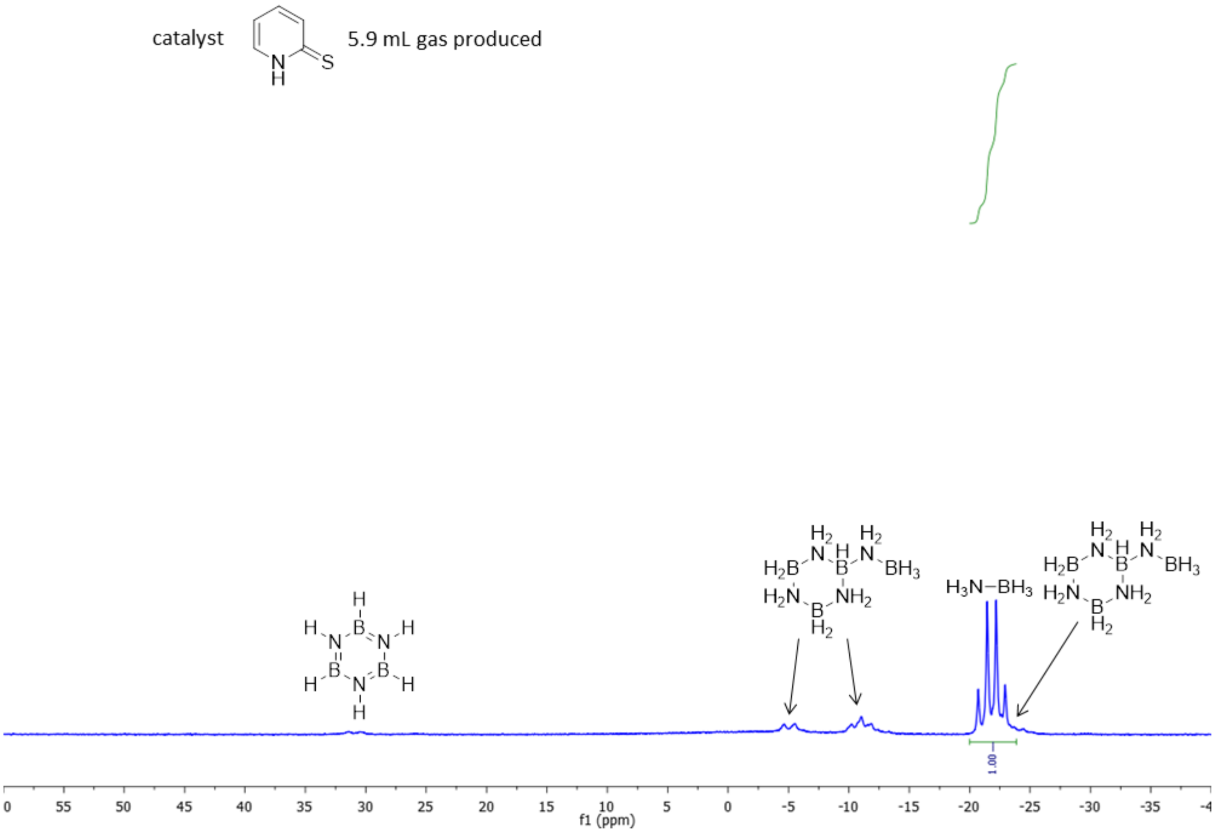

Figure SI 47: ^{11}B NMR spectrum of the first dehydrogenation experiment of AB with 2-pyridithion as catalyst (128 MHz, THF with benzene-d_6 glass capillary). Formal charges are omitted for clarity.

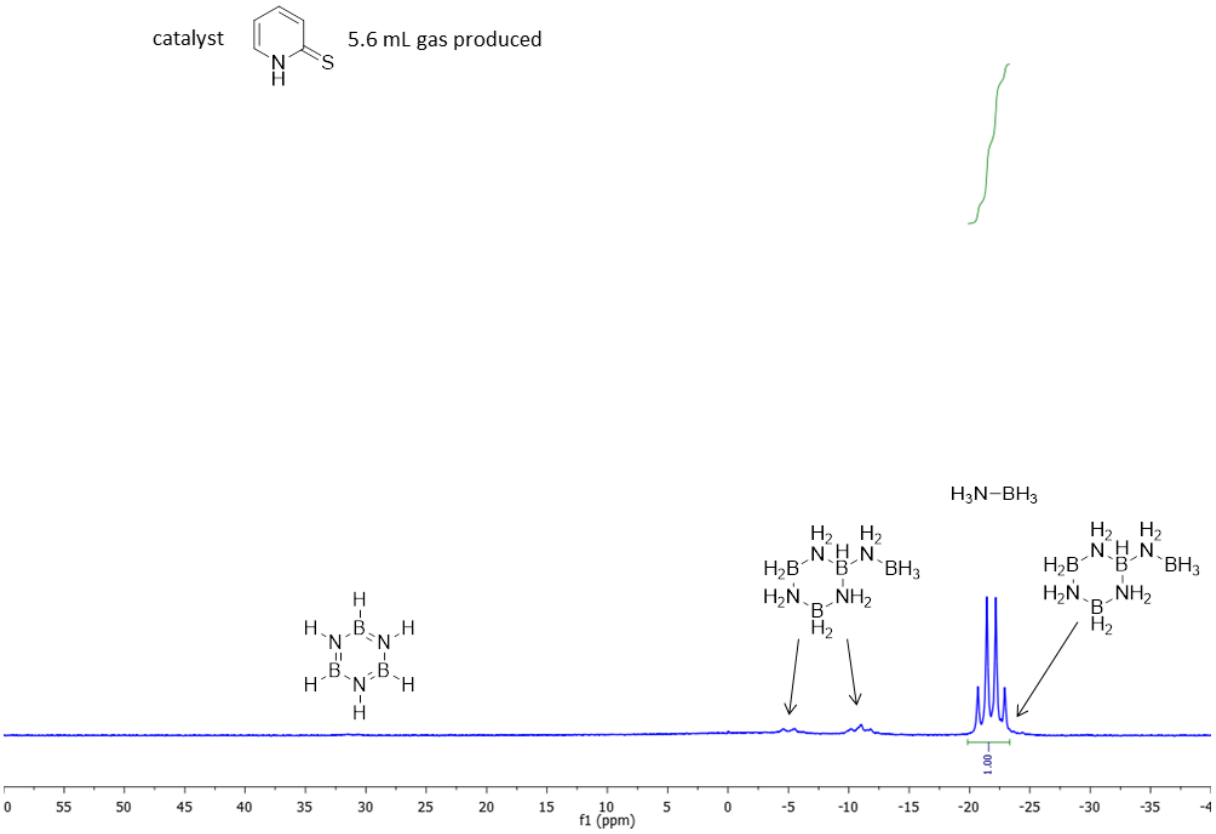

Figure SI 48: ^{11}B NMR spectrum of the first dehydrogenation experiment of AB with 2-pyridithion as catalyst (128 MHz, THF with benzene-d_6 glass capillary). Formal charges are omitted for clarity.

16.2 NMR spectra of the reaction mixture with intermediate 5

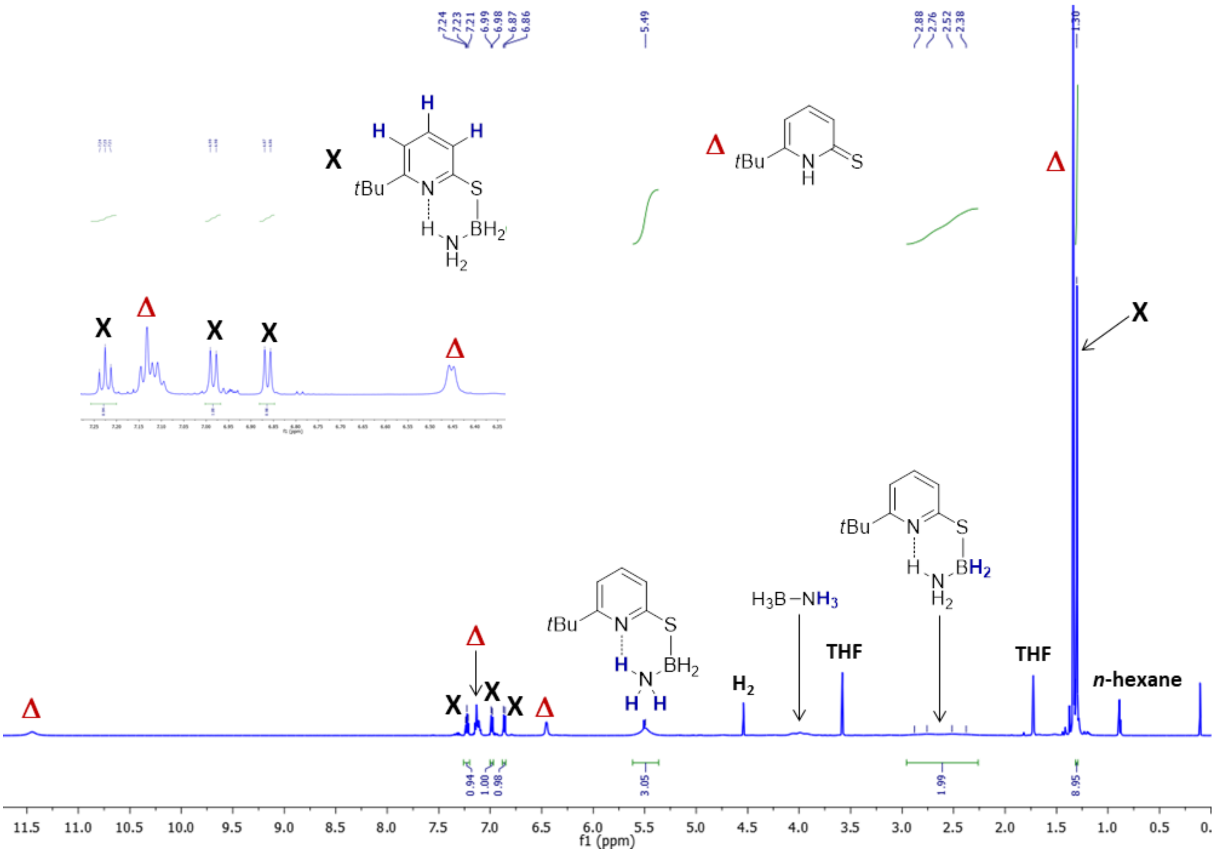

Figure SI 49: ^{1}H NMR spectrum of the reaction mixture with the intermediate **5** (600 MHz, THF-d_8). Formal charges are omitted for clarity.

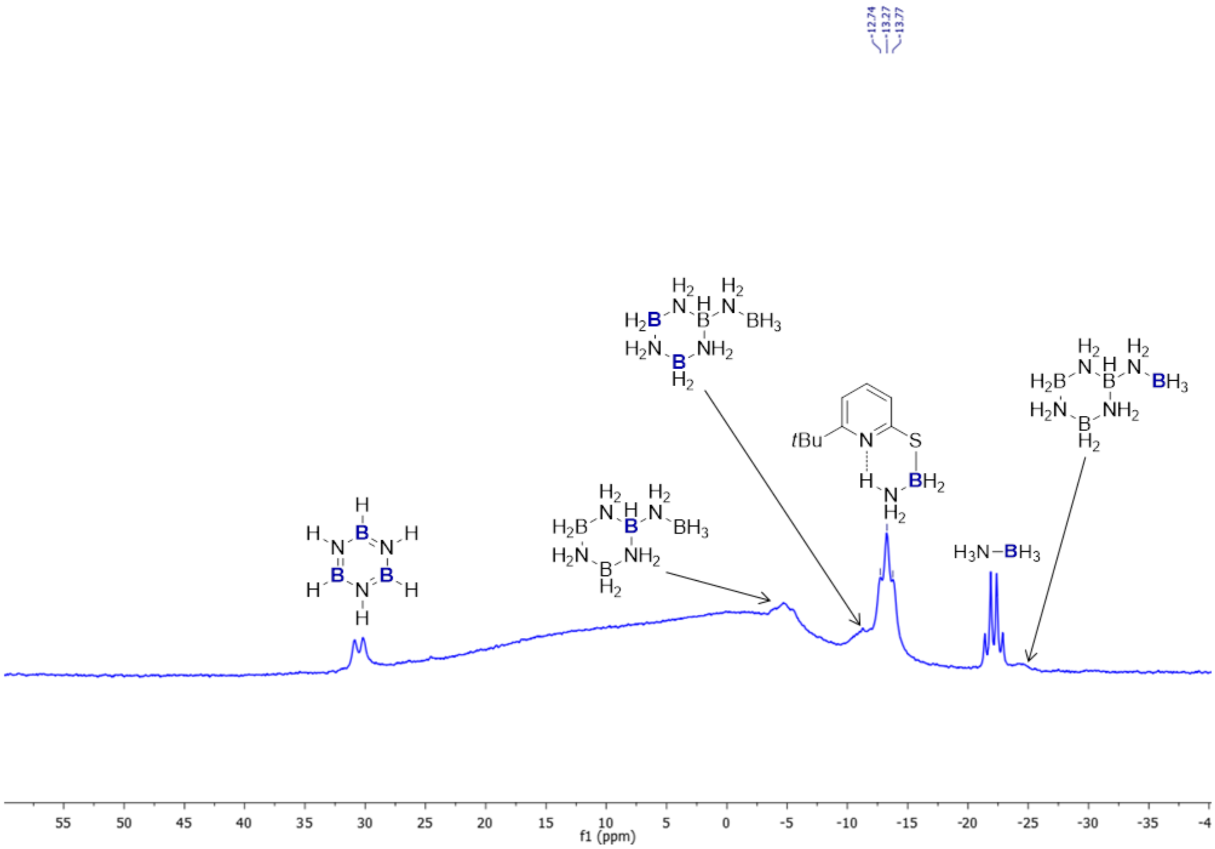

Figure SI 50: ^{11}B NMR spectrum of the reaction mixture with intermediate **5** (193 MHz, THF-d_8). Formal charges are omitted for clarity.

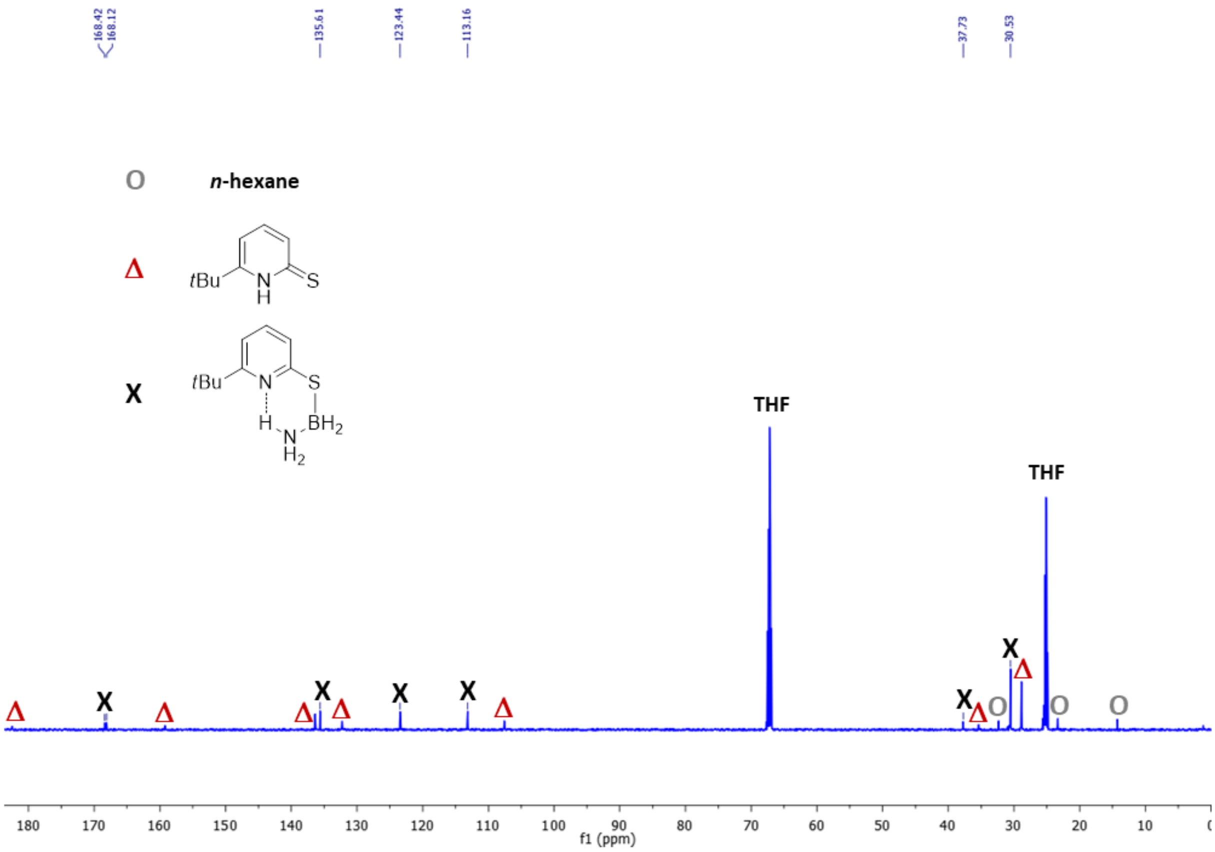

Figure SI 51: ^{13}C NMR spectrum of the reaction mixture with intermediate **5** (151 MHz, THF-d_8). Formal charges are omitted for clarity.

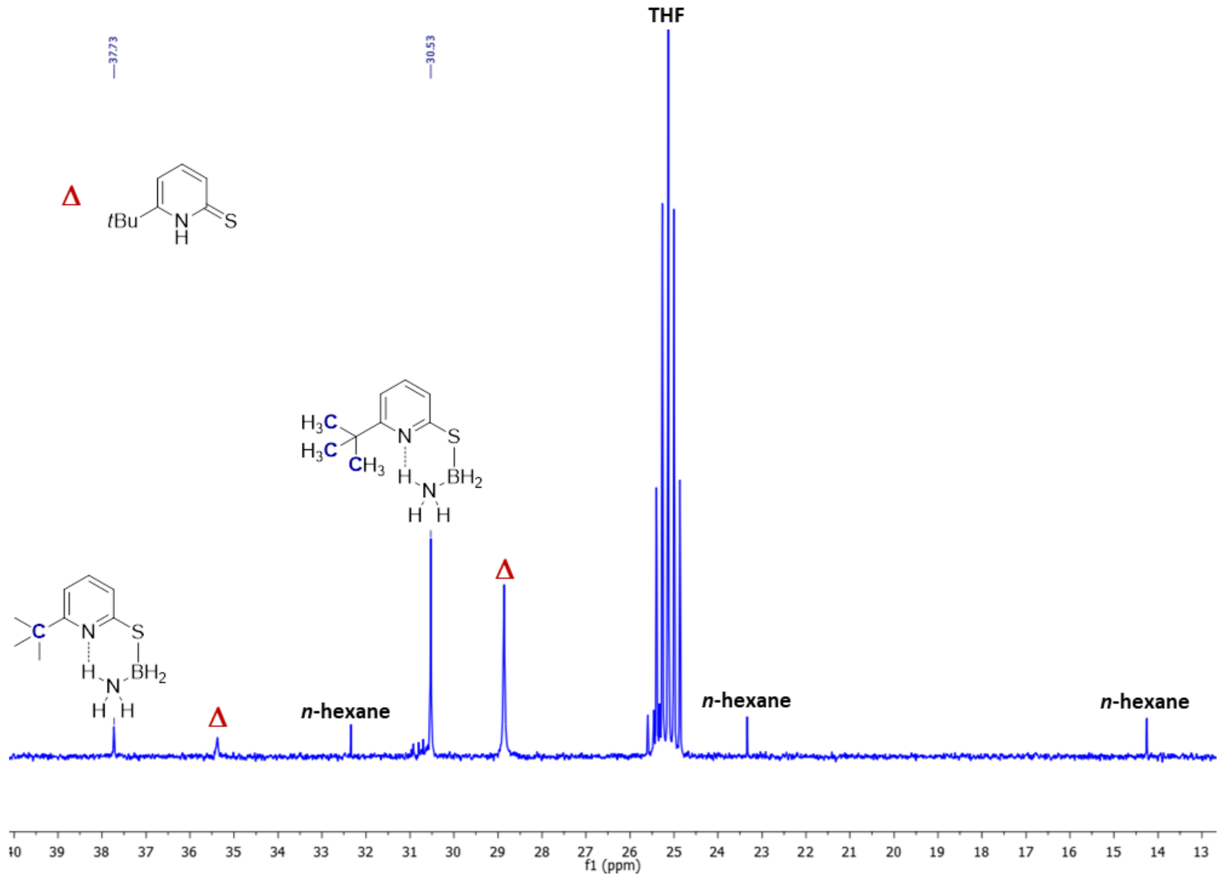

Figure SI 52: Excerpt of the aliphatic region of the ^{13}C NMR spectrum of the reaction mixture with intermediate **5** (151 MHz, THF-d_8). Formal charges are omitted for clarity.

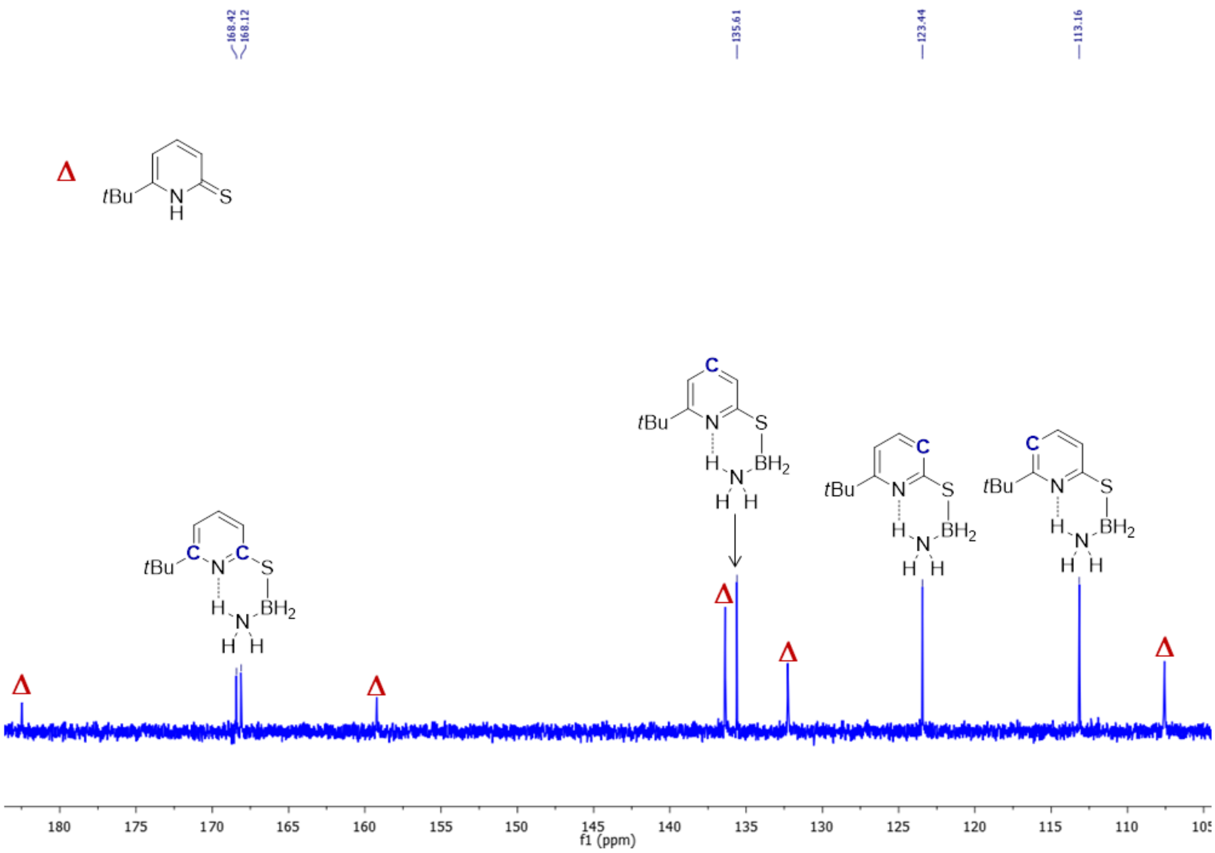

Figure SI 53: Excerpt of the aromatic region of the ^{13}C NMR spectrum of the reaction mixture with intermediate **5** (151 MHz, THF-d_8). Formal charges are omitted for clarity.

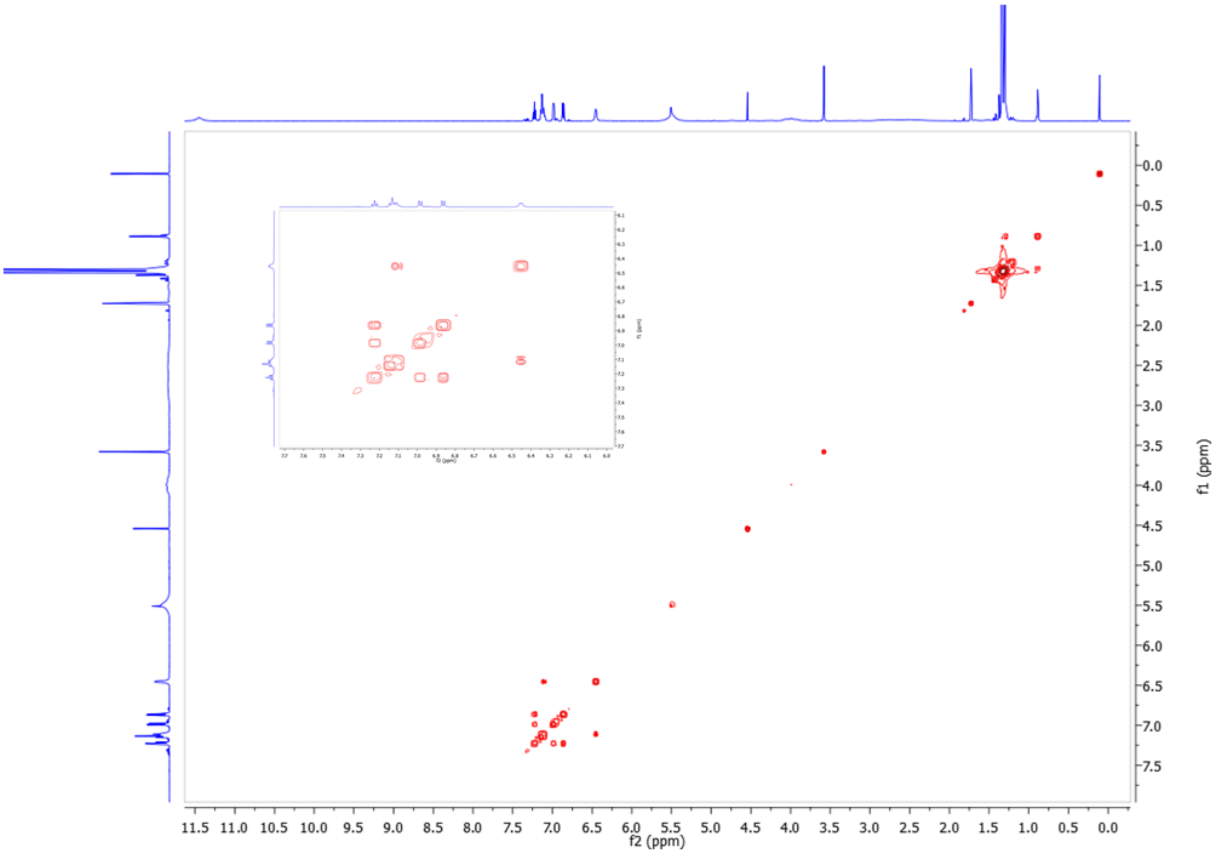

Figure SI 54: HH COSY NMR spectrum of the reaction mixture with intermediate **5** with inset of the aromatic region (600 MHz, THF-d_8). Formal charges are omitted for clarity.

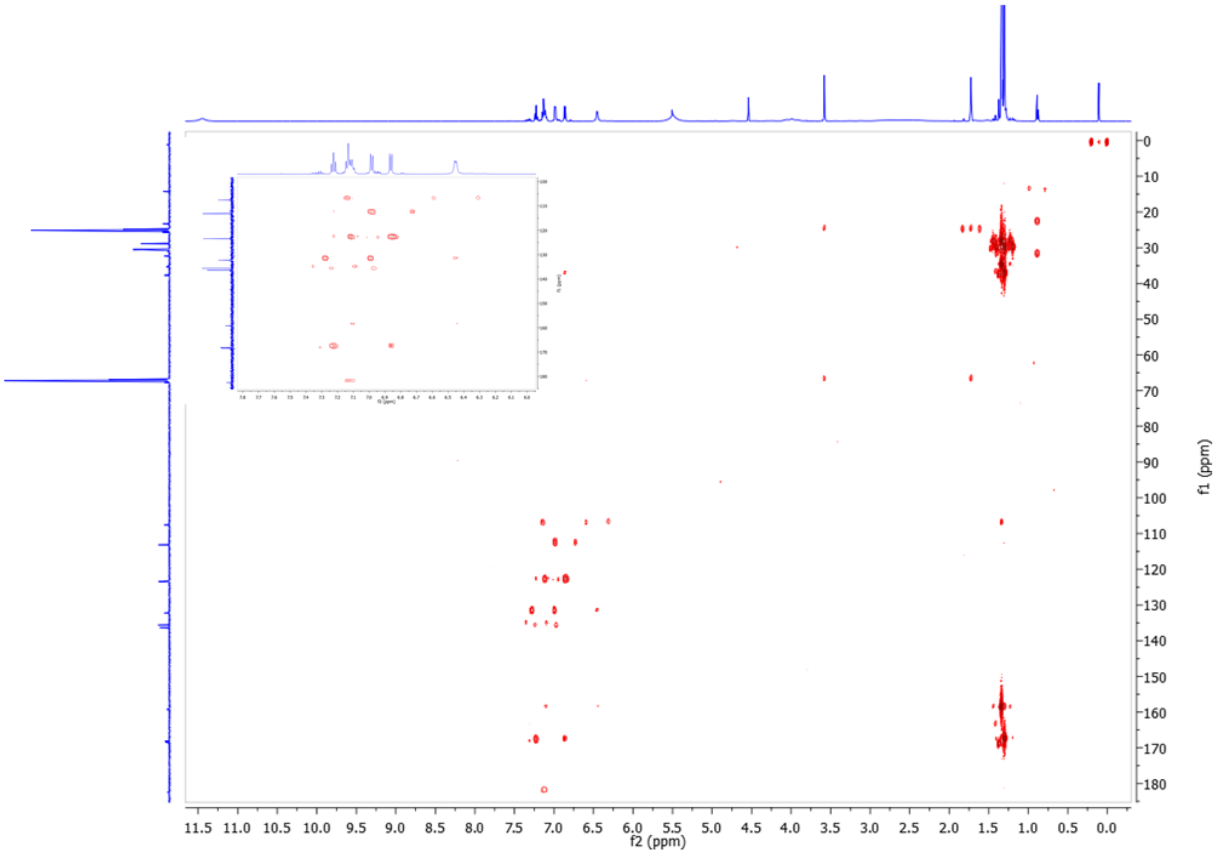

Figure SI 55: $^{1}H^{13}C$ HMBC NMR spectrum of the reaction mixture with intermediate **5** with inset of the aromatic region (151 MHz, THF-d_8). Formal charges are omitted for clarity.

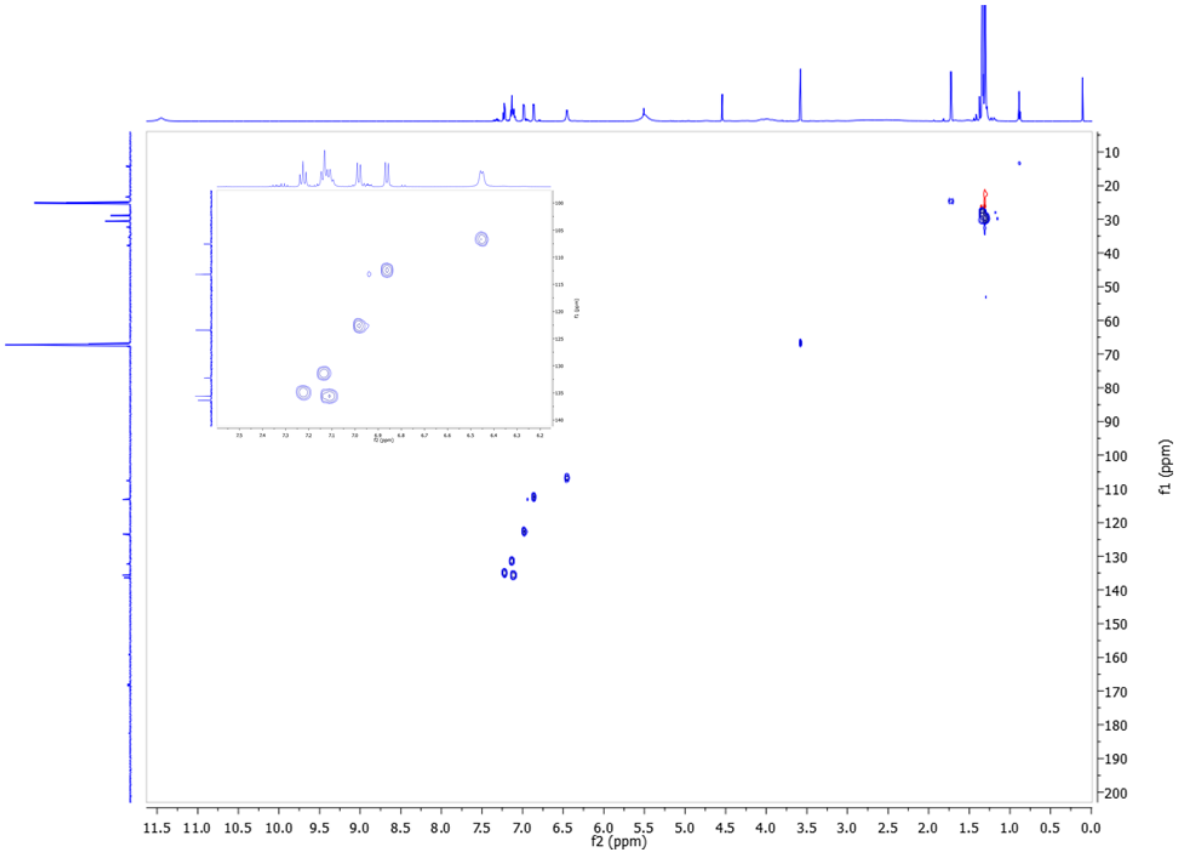

Figure SI 56: $^{1}H^{13}C$ HSQC NMR spectrum of the reaction mixture with intermediate **5** with inset of the aromatic region (151 MHz, THF-d_8). Formal charges are omitted for clarity.

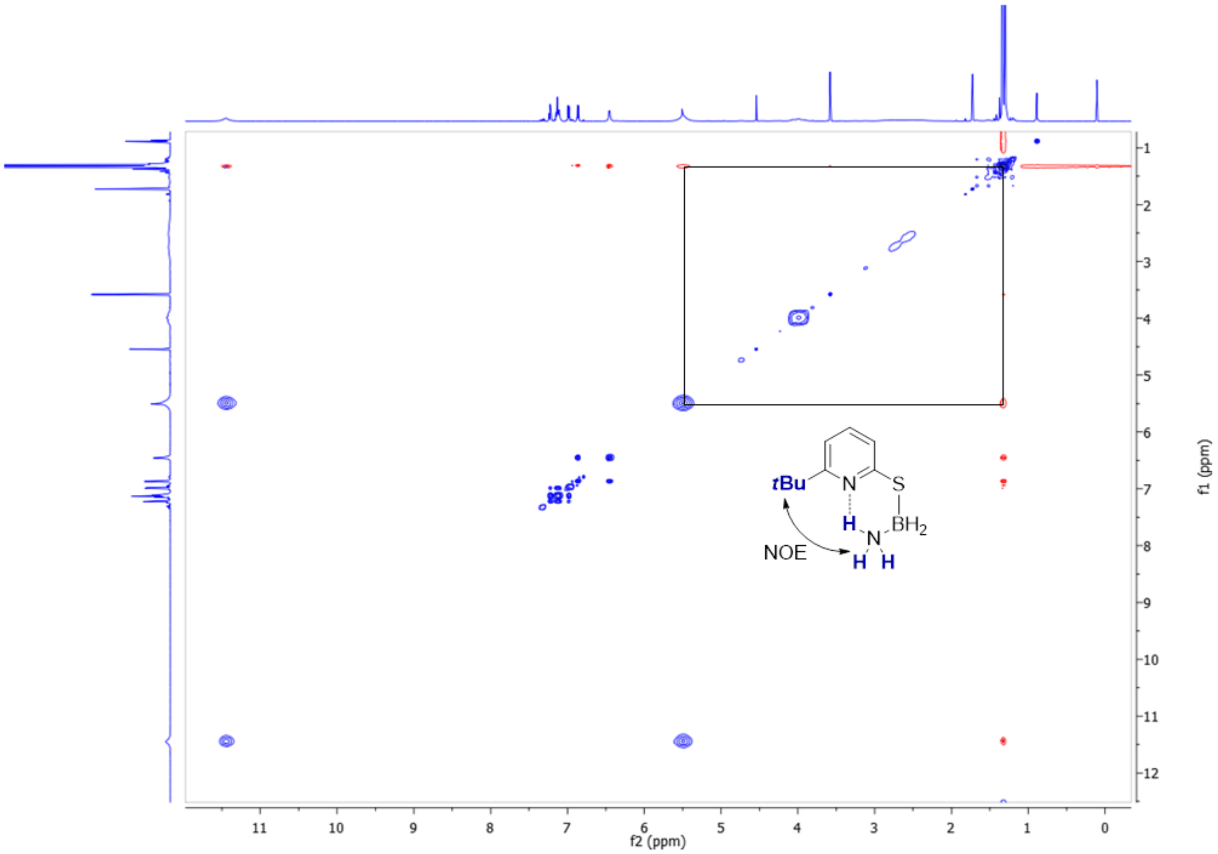

Figure SI 57: HH NOESY NMR spectrum of the reaction mixture with intermediate **5.** (600 MHz, THF-d_8). Formal charges are omitted for clarity.

16.3 NMR spectra of surrogate intermediate 5_{Me2}

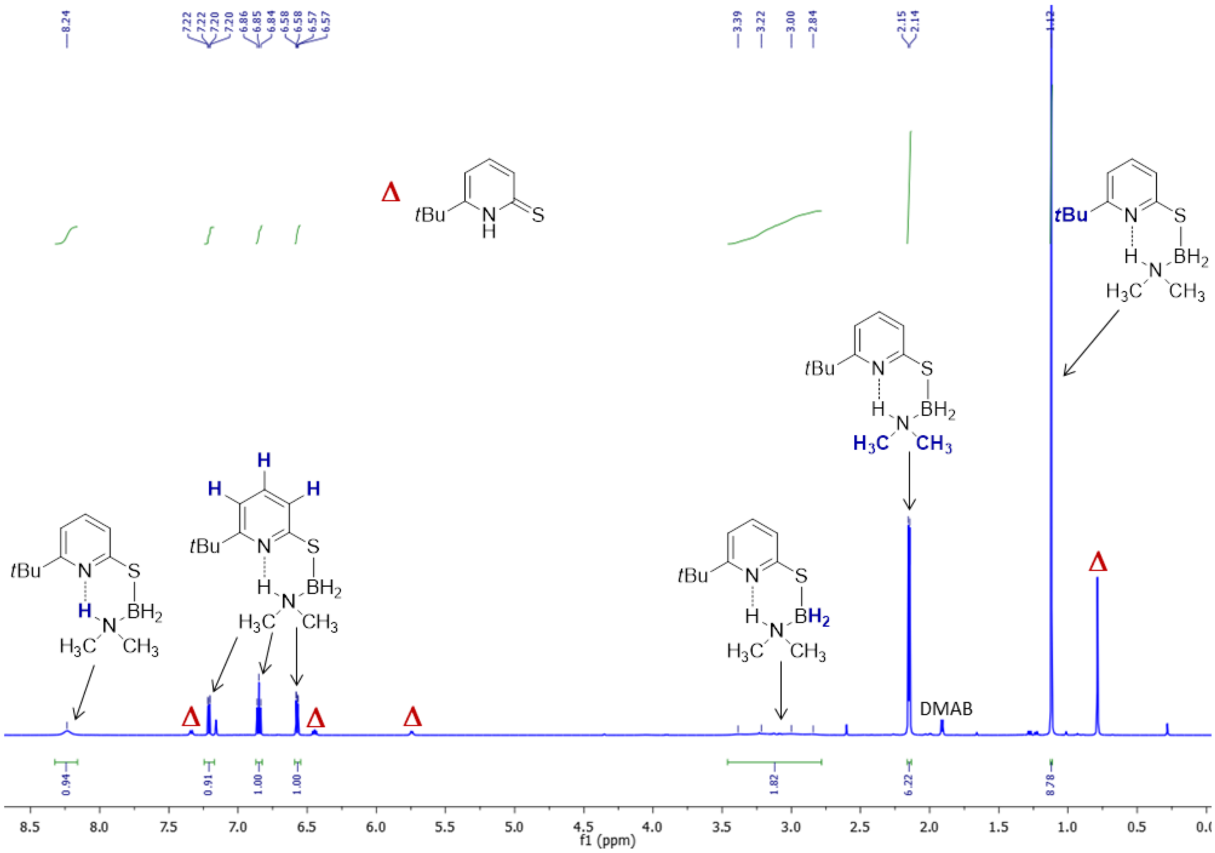

Figure SI 58: ^{1}H NMR spectrum of surrogate intermediate $\mathbf{5_{Me2}}$ (600 MHz, benzene-d_6). Formal charges are omitted for clarity.

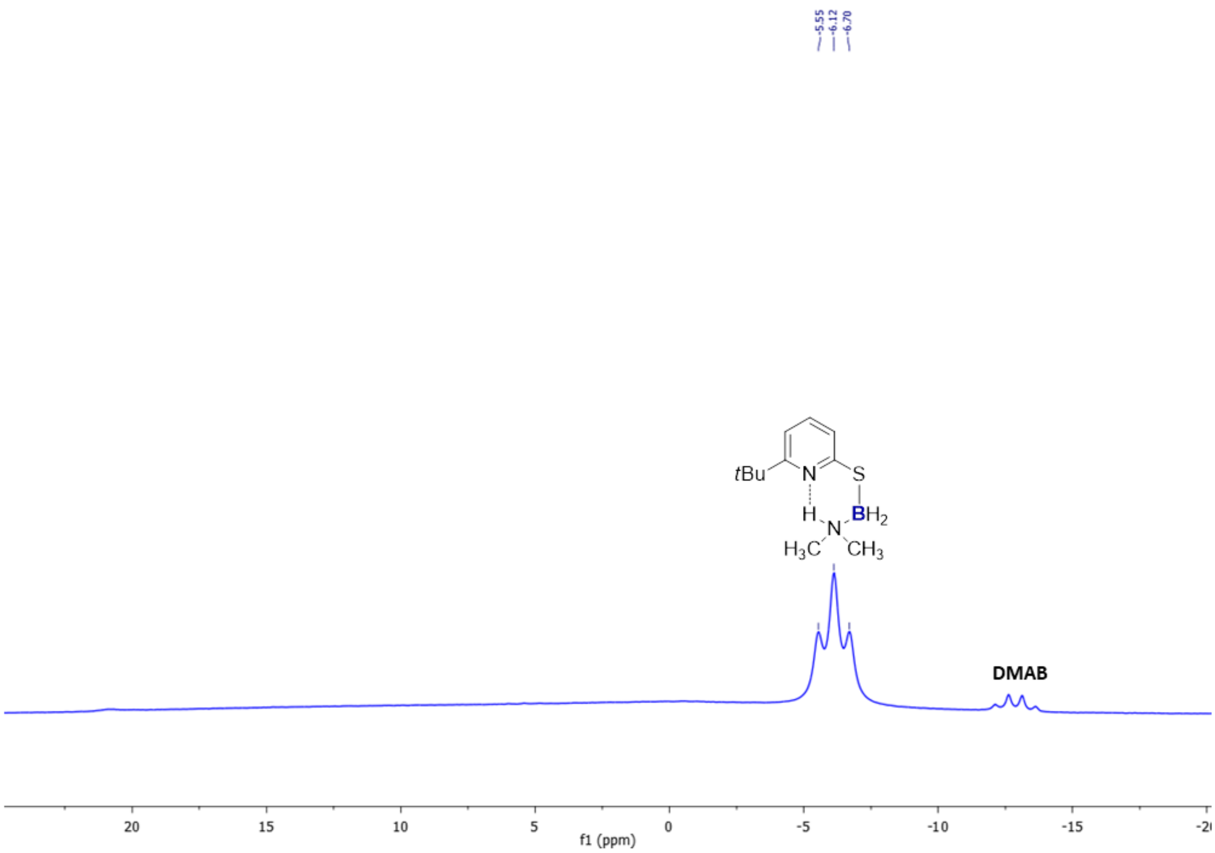

Figure SI 59: ^{11}B NMR spectrum of surrogate intermediate $\mathbf{5_{Me2}}$ (193 MHz, benzene-d_6). Formal charges are omitted for clarity.

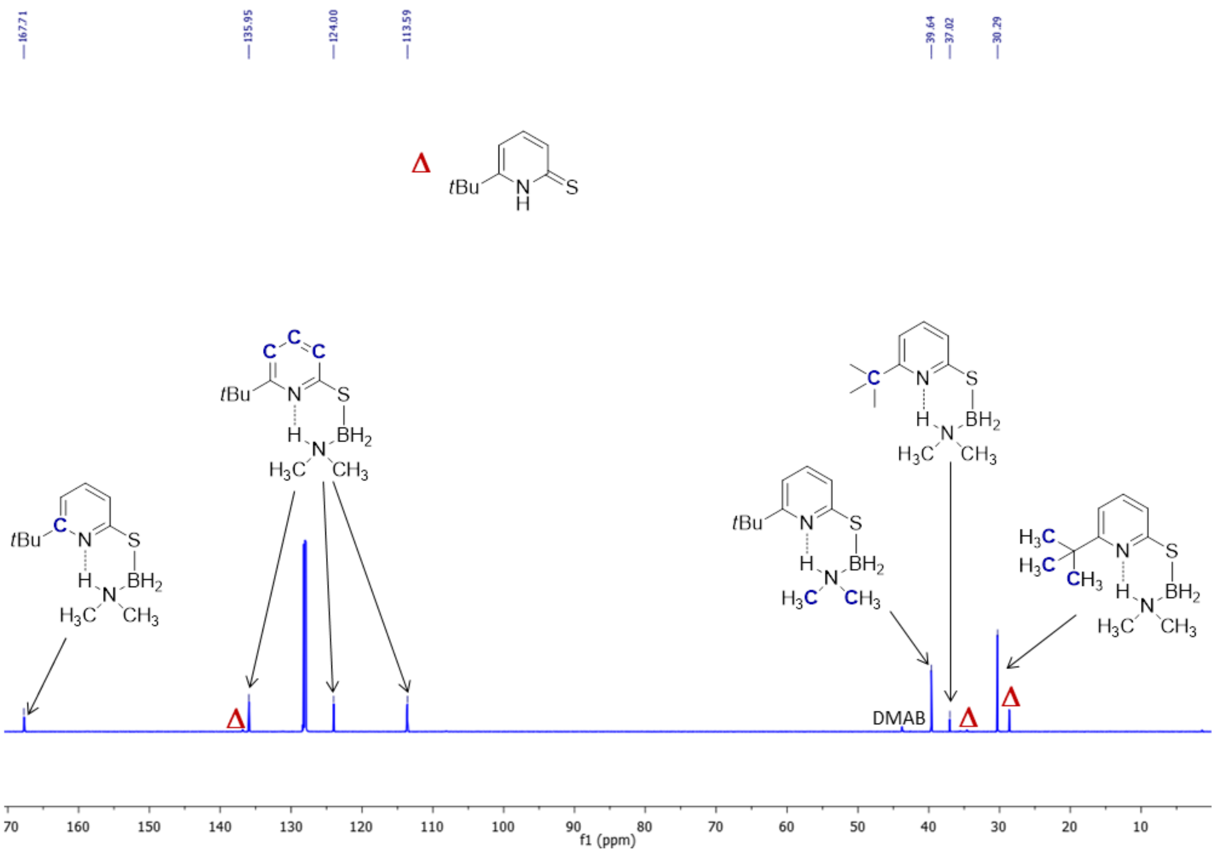

Figure SI 60: ^{13}C NMR spectrum of surrogate intermediate $\mathbf{5_{Me2}}$ (151 MHz, benzene-d_6). Formal charges are omitted for clarity.

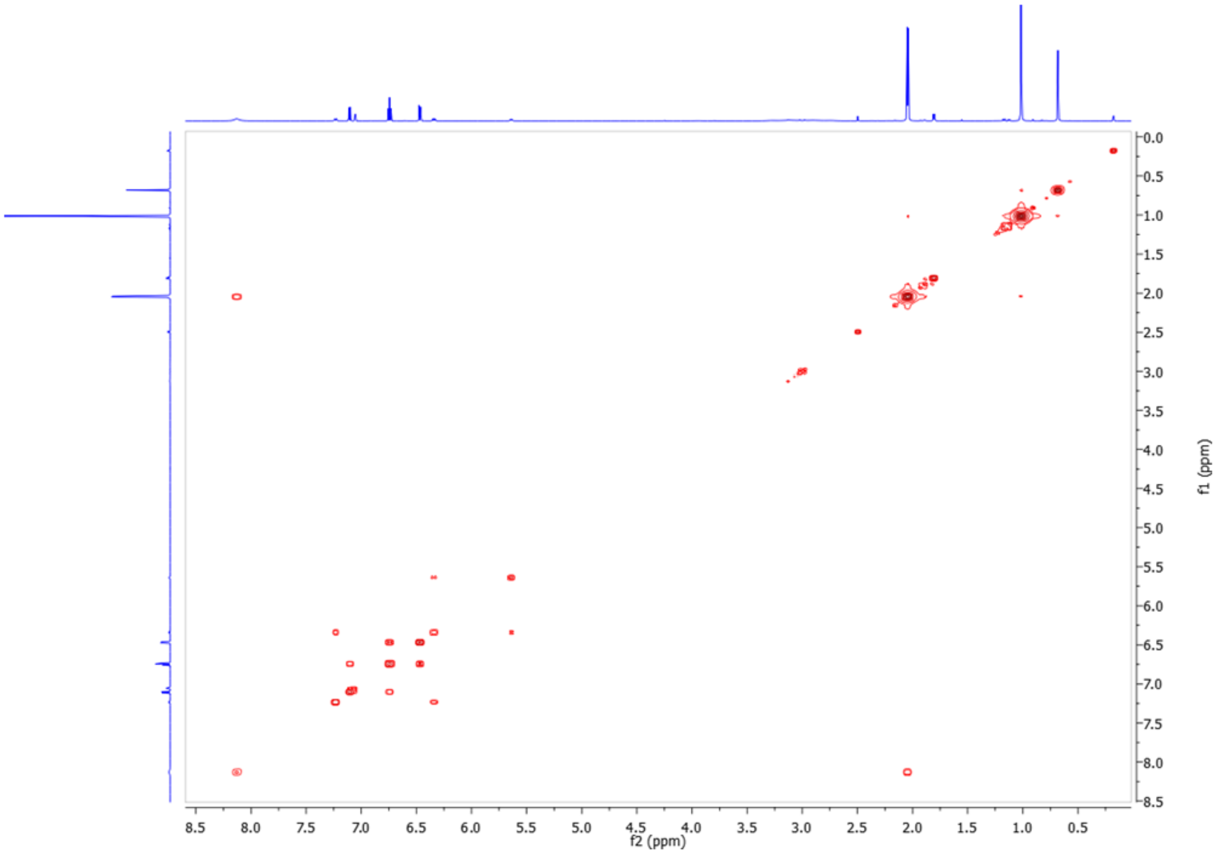

Figure SI 61: HH COSY spectrum of surrogate intermediate $\mathbf{5_{Me2}}$ (600 MHz, benzene-d_6). Formal charges are omitted for clarity.

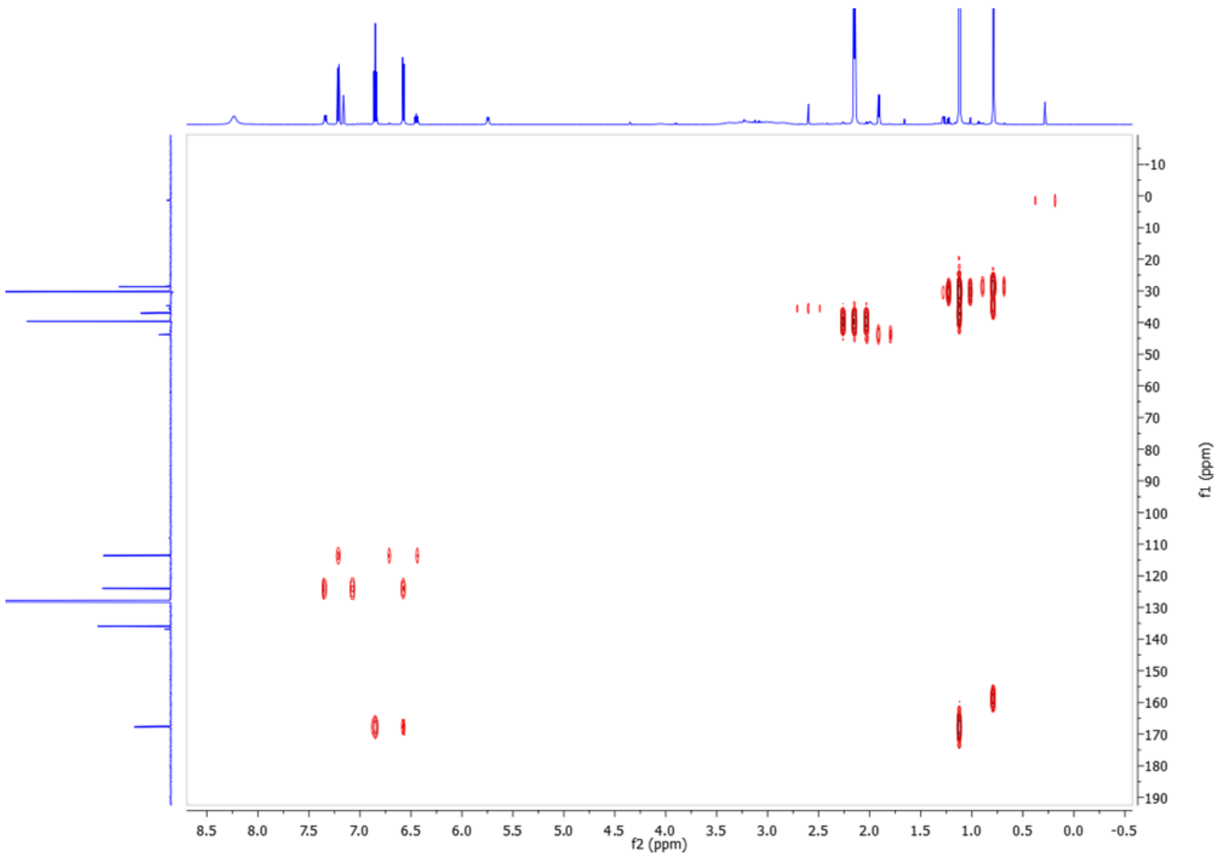

Figure SI 62: HMBC NMR spectrum of surrogate intermediate $\mathbf{5_{Me2}}$ (151 MHz, benzene-d_6). Formal charges are omitted for clarity.

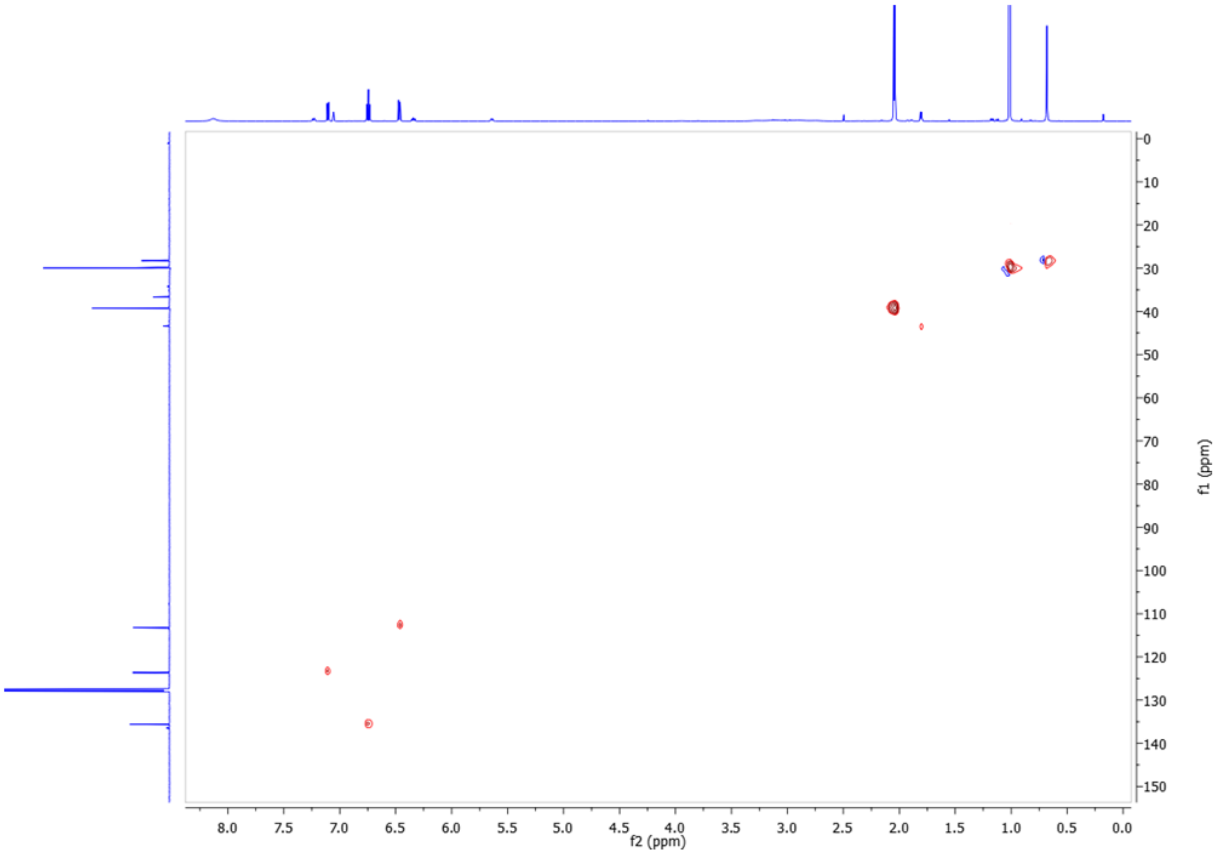

Figure SI 63: HMBC NMR spectrum of surrogate intermediate **5_{Me2}** (151 MHz, benzene-d_6). Formal charges are omitted for clarity.

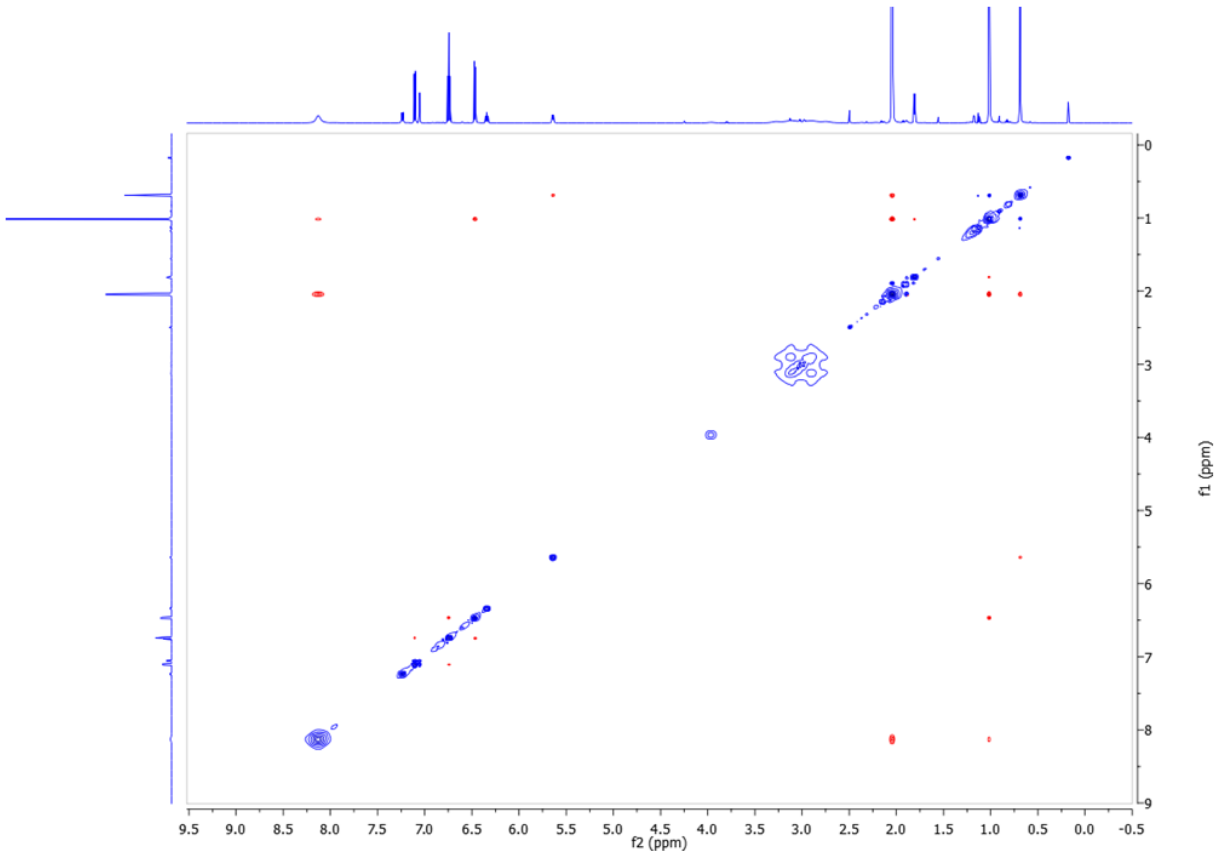

Figure SI 64: HH NOESY NMR spectrum of surrogate intermediate $\mathbf{5}_{\mathbf{Me2}}$ (600 MHz, benzene-d_6). Formal charges are omitted for clarity.

16.4 NMR spectra of the determination of the kinetic isotope effects

1. catalytic dehydrogenation of AB
   reaction time: 10 min

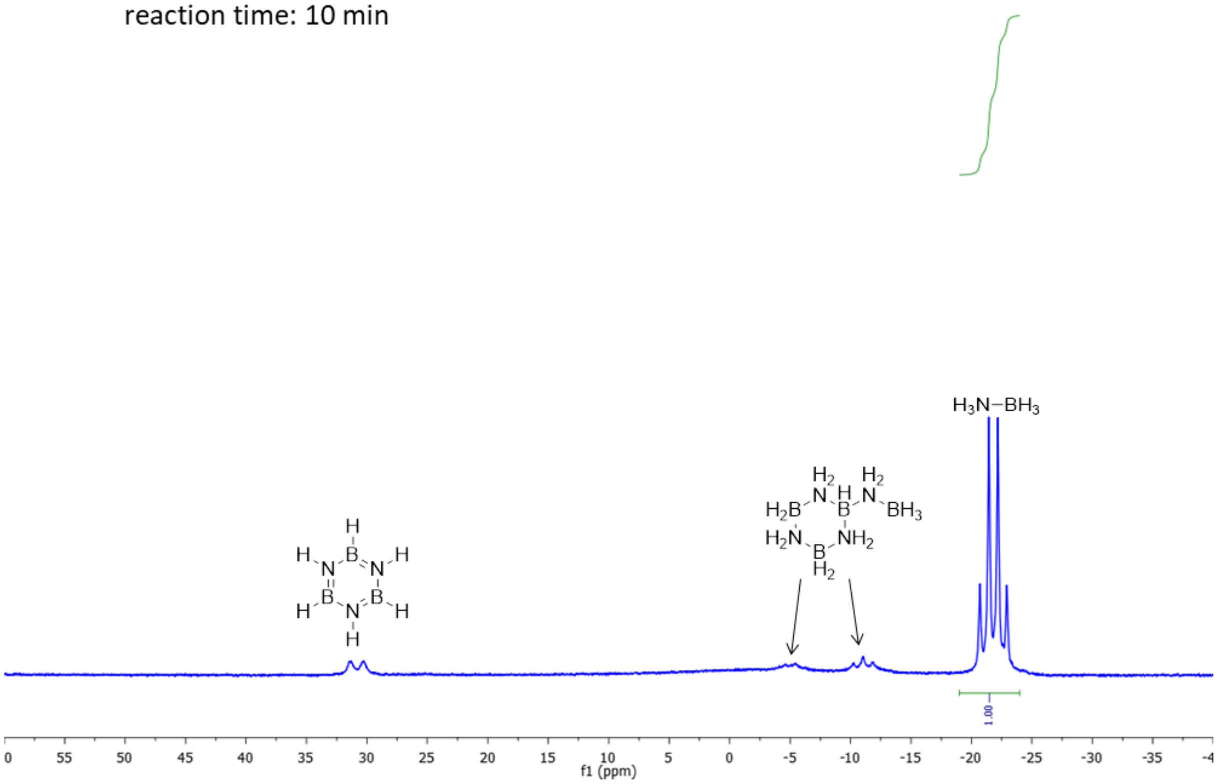

Figure SI 65: ^{11}B NMR of the first catalytic dehydrogenation of AB after 10 min (128 MHz, THF with benzene-d_6 glass capillary). Formal charges are omitted for clarity.

2. catalytic dehydrogenation of AB
reaction time: 10 min

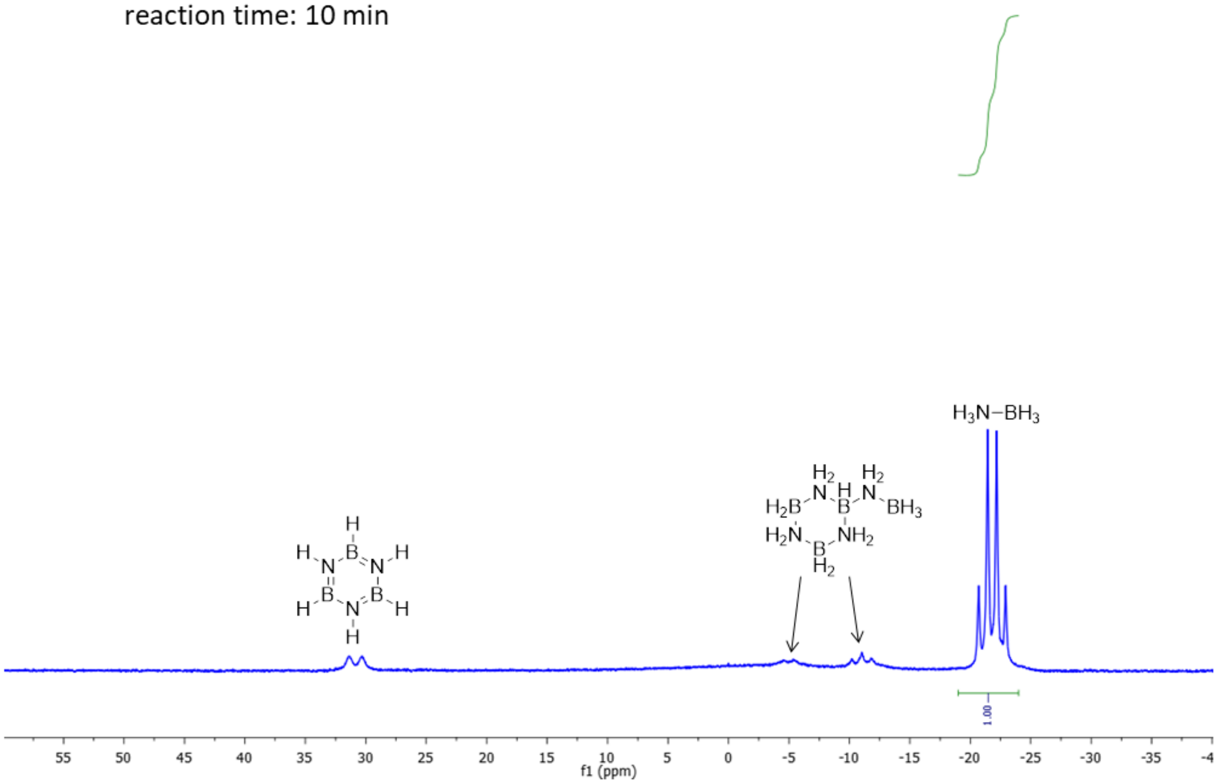

Figure SI 66: ^{11}B NMR of the second catalytic dehydrogenation of AB after 10 min (128 MHz, THF with benzene-d_6 glass capillary). Formal charges are omitted for clarity.

1. catalytic dehydrogenation of AB
reaction time: 20 min

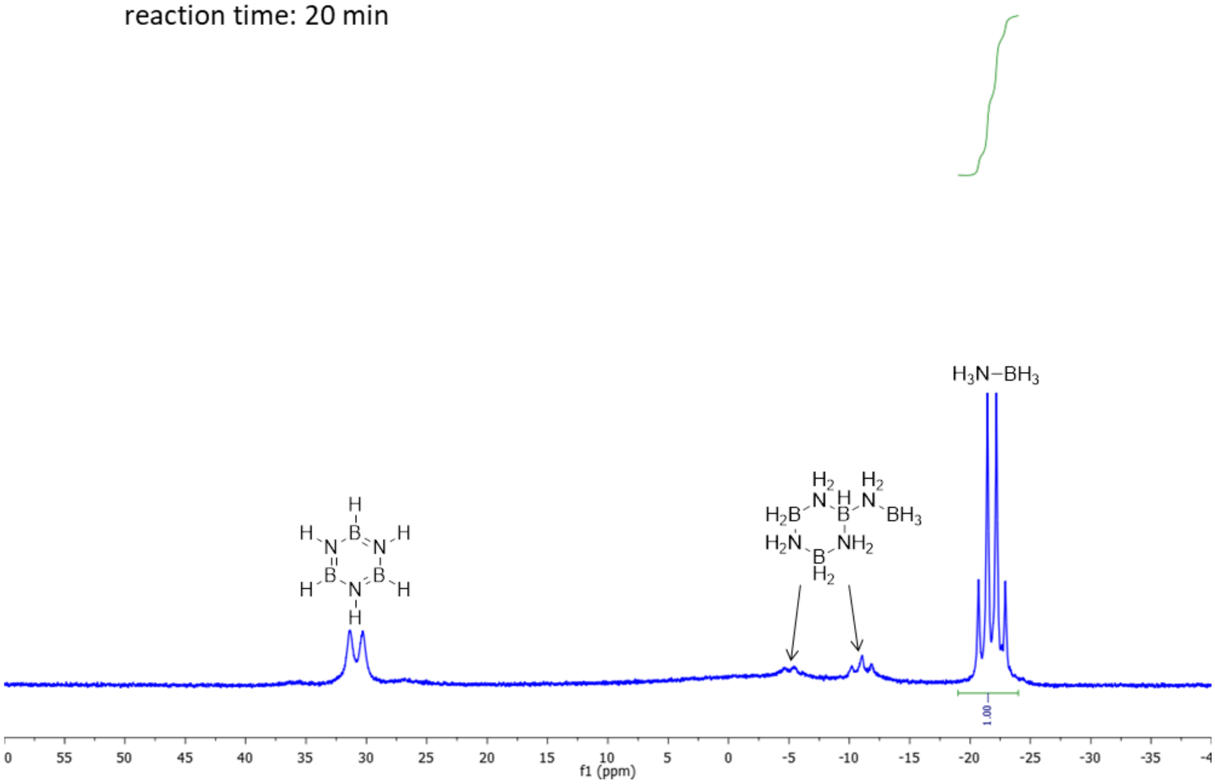

Figure SI 67: ^{11}B NMR of the first catalytic dehydrogenation of AB after 20 min (128 MHz, THF with benzene-d_6 glass capillary). Formal charges are omitted for clarity.

2. catalytic dehydrogenation of AB
reaction time: 20 min

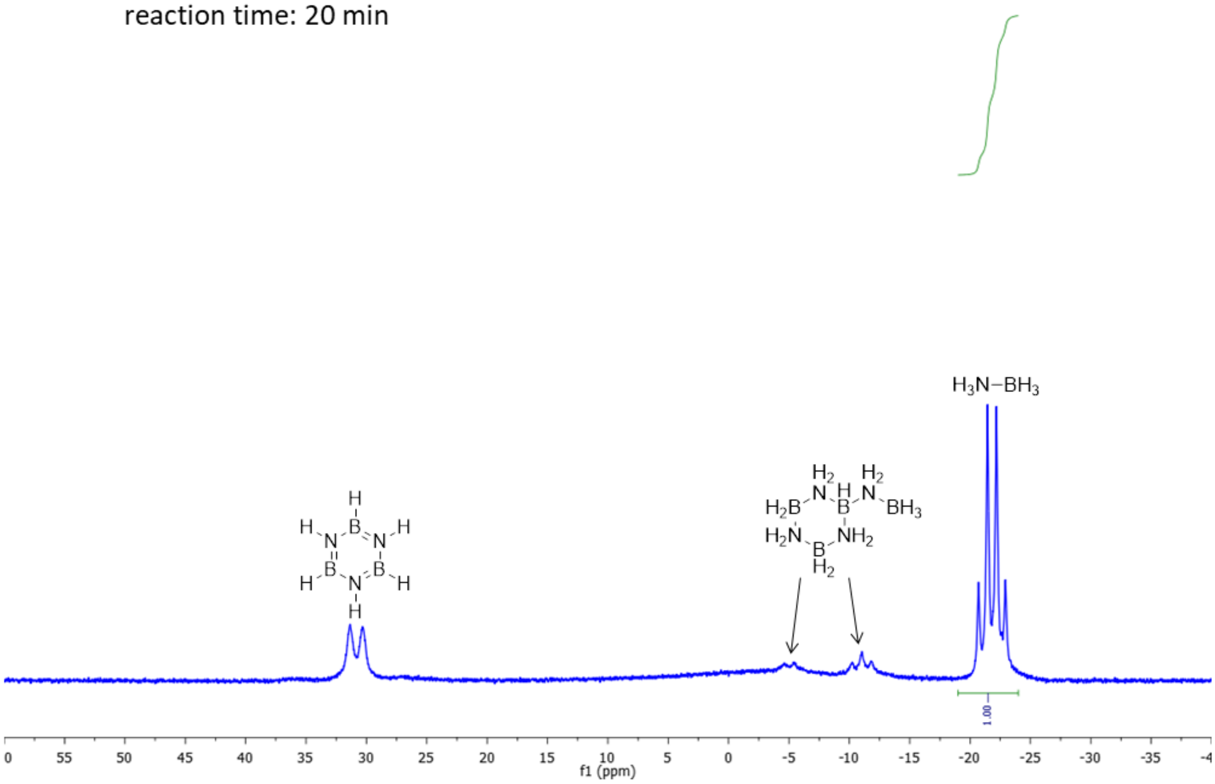

Figure SI 68: ^{11}B NMR of the second catalytic dehydrogenation of AB after 20 min (128 MHz, THF with benzene-d_6 glass capillary). Formal charges are omitted for clarity.

1. catalytic dehydrogenation of AB
   reaction time: 30 min

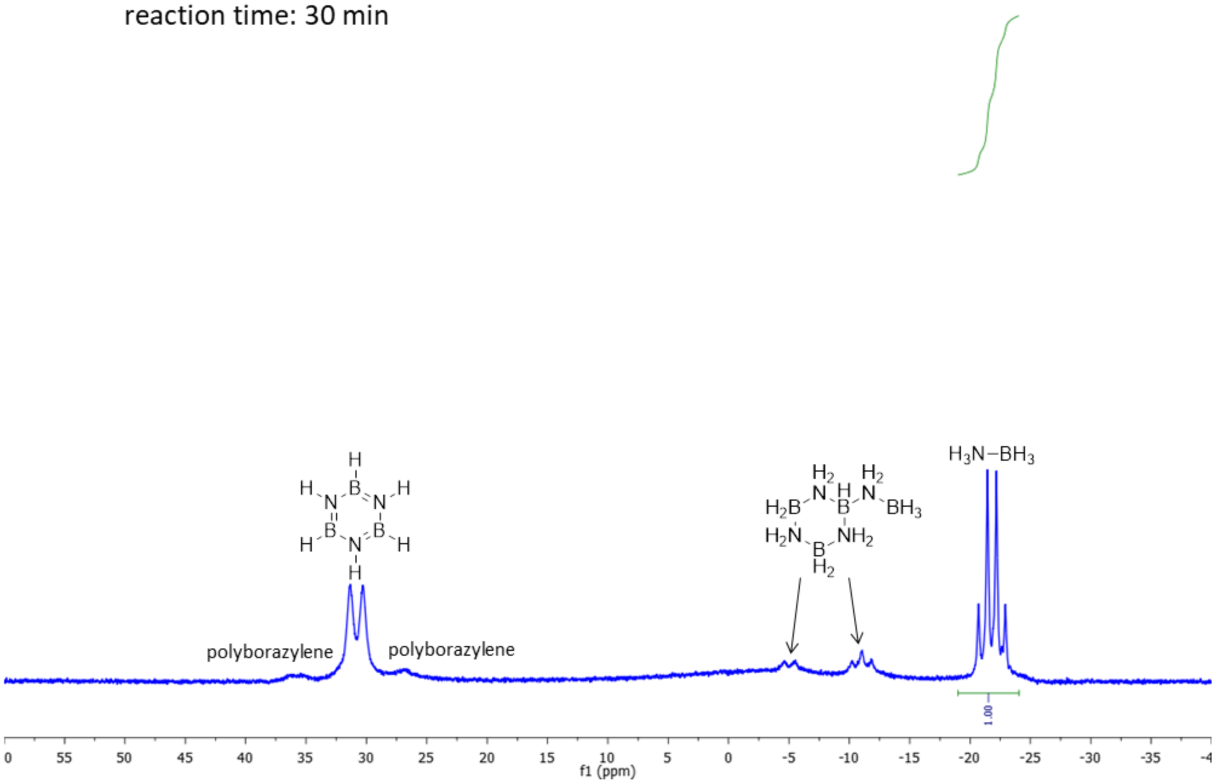

Figure SI 69: ^{11}B NMR of the first catalytic dehydrogenation of AB after 30 min (128 MHz, THF with benzene-d_6 glass capillary). Formal charges are omitted for clarity.

2. catalytic dehydrogenation of AB
reaction time: 30 min

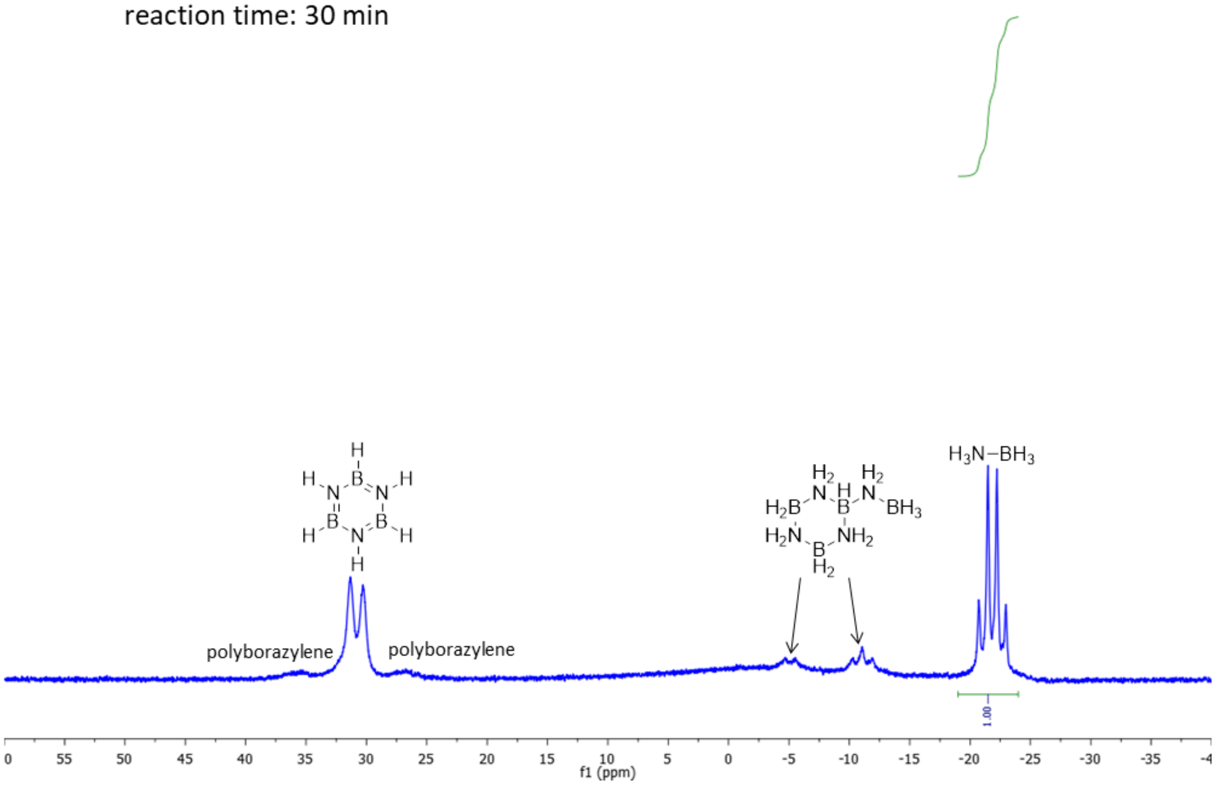

Figure SI 70: ^{11}B NMR of the second catalytic dehydrogenation of AB after 30 min (128 MHz, THF with benzene-d_6 glass capillary). Formal charges are omitted for clarity.

1. catalytic dehydrogenation of AB
reaction time: 40 min

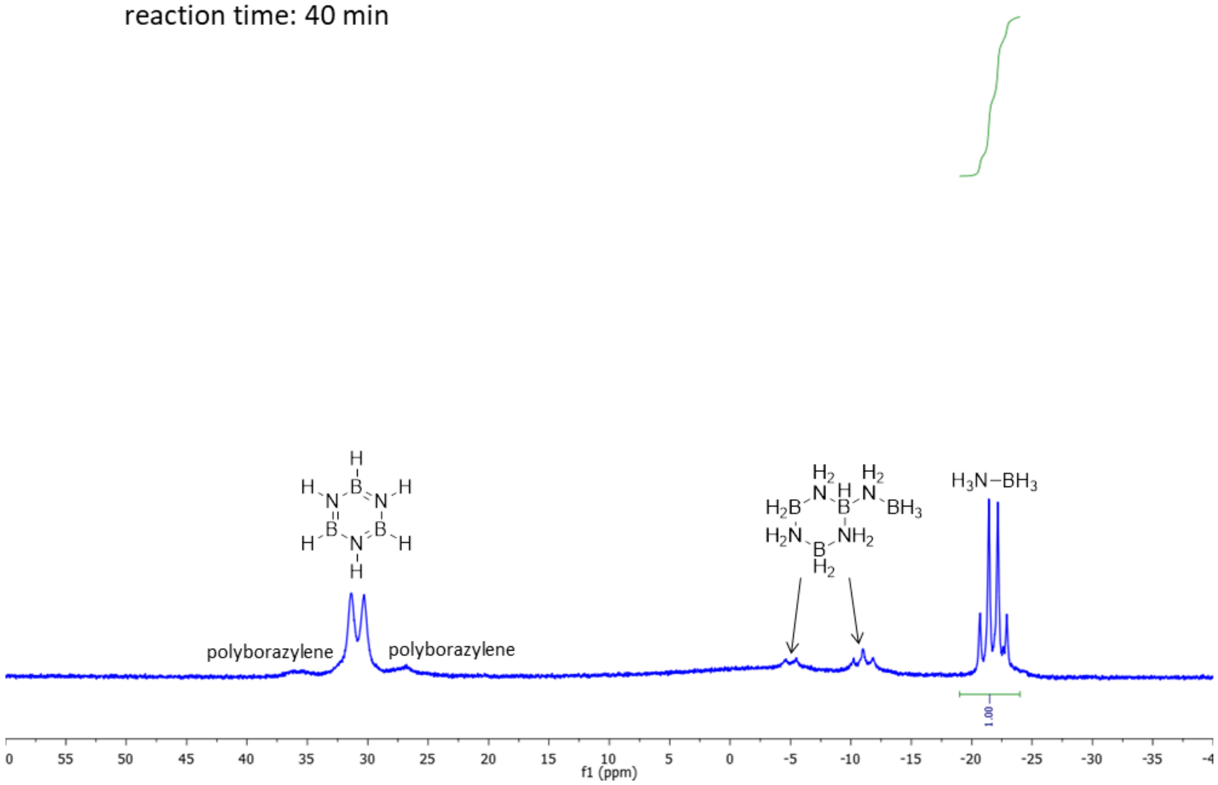

Figure SI 71: ^{11}B NMR of the first catalytic dehydrogenation of AB after 40 min (128 MHz, THF with benzene-d_6 glass capillary). Formal charges are omitted for clarity.

2. catalytic dehydrogenation of AB
reaction time: 40 min

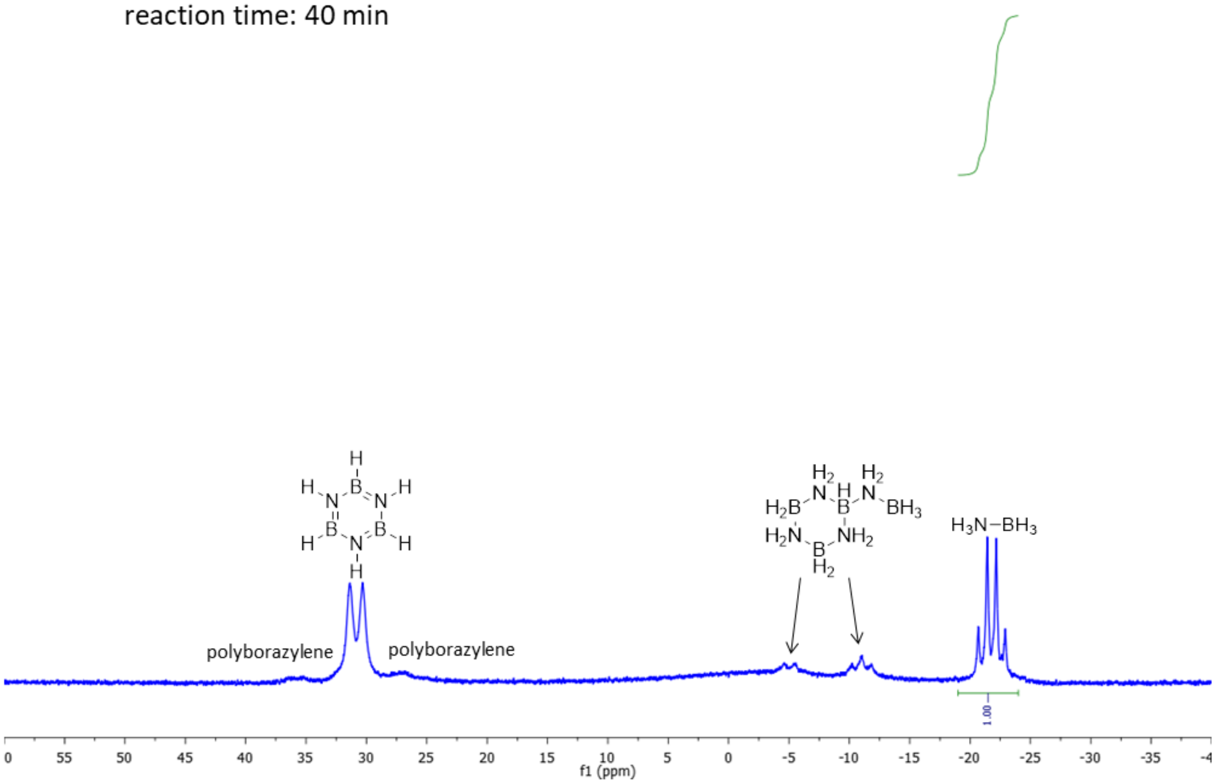

Figure SI 72: ^{11}B NMR of the second catalytic dehydrogenation of AB after 40 min (128 MHz, THF with benzene-d_6 glass capillary). Formal charges are omitted for clarity.

1. catalytic dehydrogenation of H_3NBD_3
reaction time: 10 min

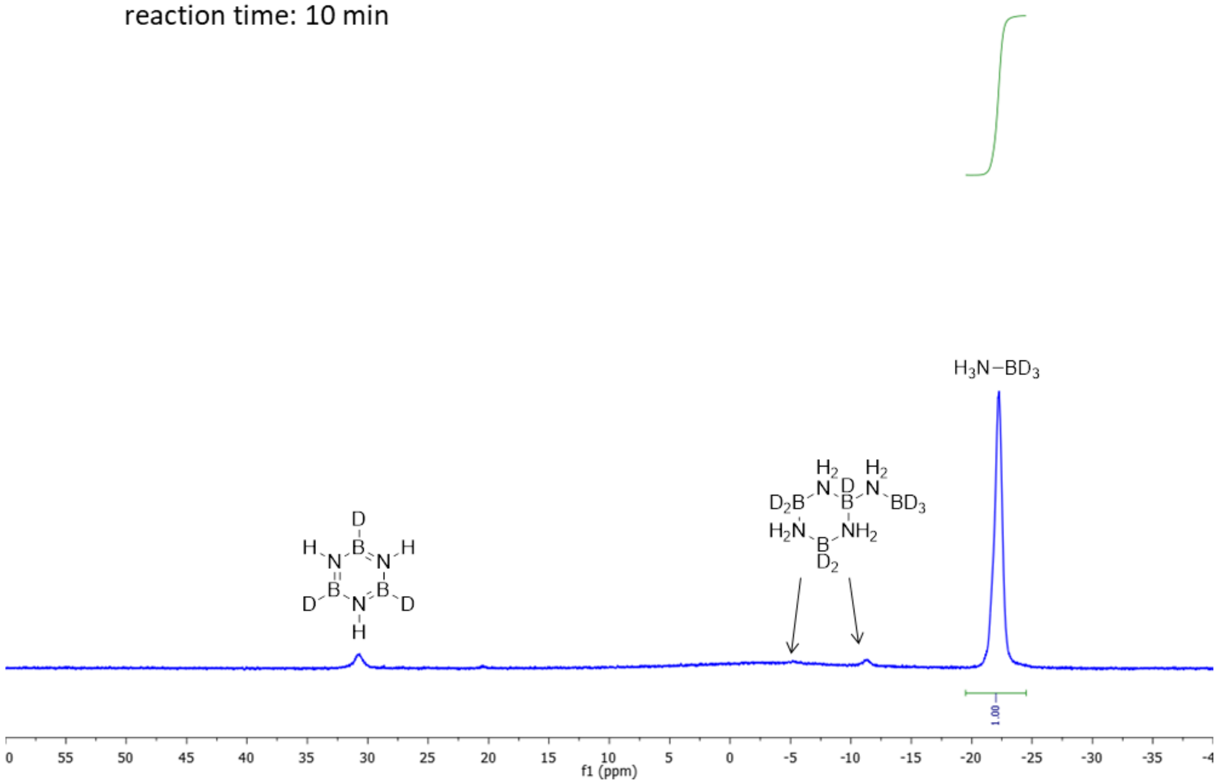

Figure SI 73: ^{11}B NMR of the first catalytic dehydrogenation of H_3NBD_3 after 10 min (128 MHz, THF with benzene-d_6 glass capillary). Formal charges are omitted for clarity.

2. catalytic dehydrogenation of H_3NBD_3
reaction time: 10 min

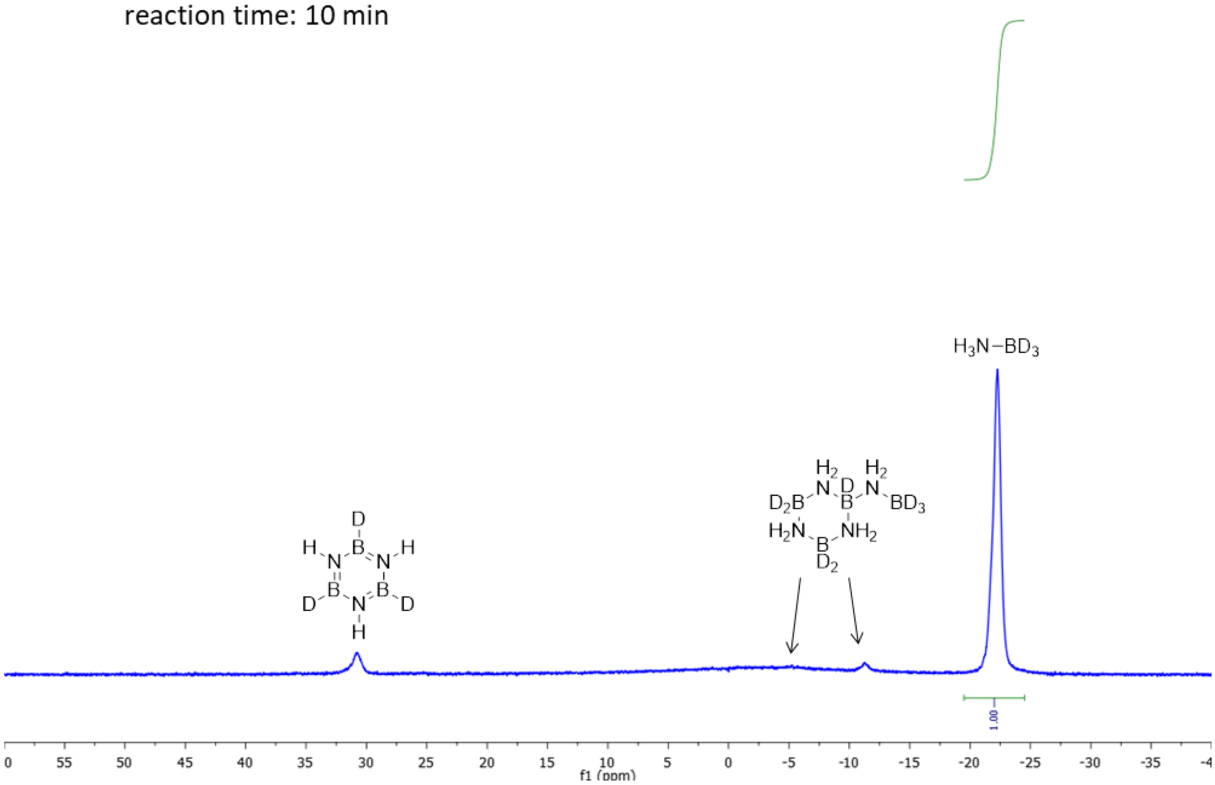

Figure SI 74: ^{11}B NMR of the second catalytic dehydrogenation of H_3NBD_3 after 10 min (128 MHz, THF with benzene-d_6 glass capillary). Formal charges are omitted for clarity.

1. catalytic dehydrogenation of H_3NBD_3
reaction time: 20 min

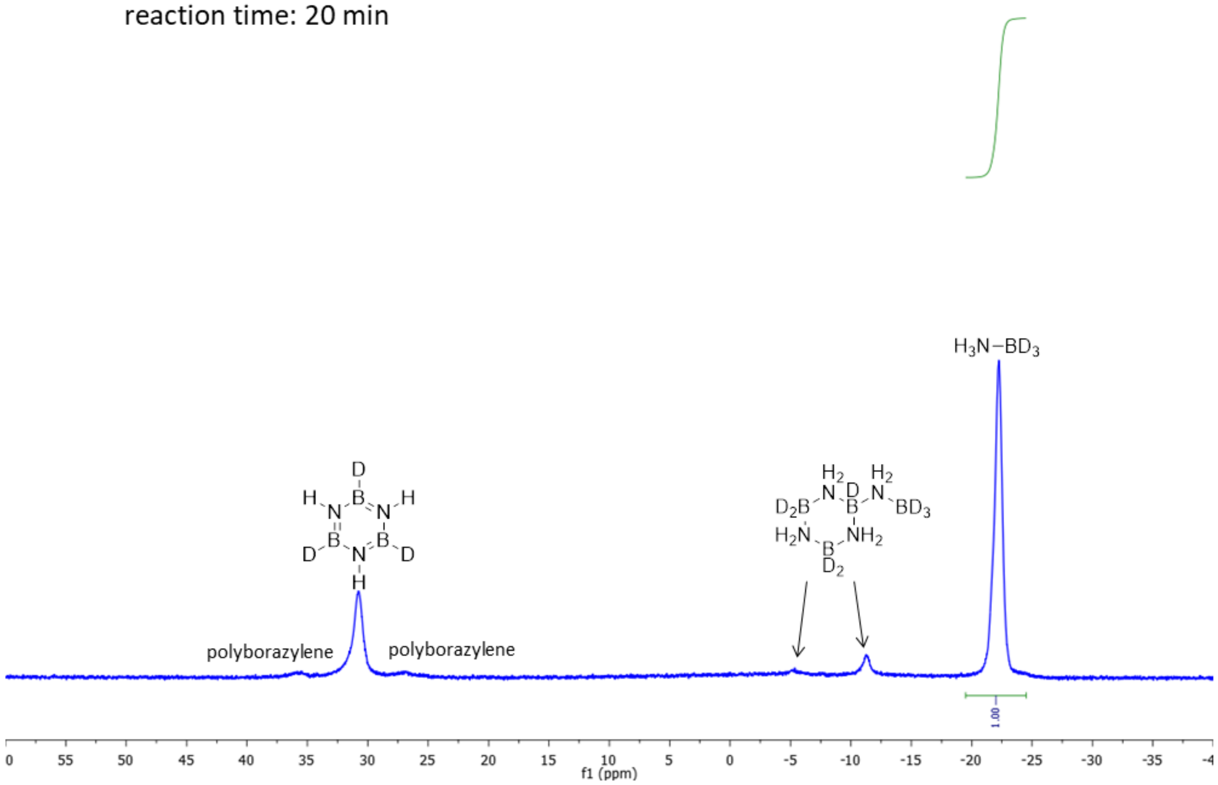

Figure SI 75: ^{11}B NMR of the first catalytic dehydrogenation of H_3NBD_3 after 20 min (128 MHz, THF with benzene-d_6 glass capillary). Formal charges are omitted for clarity.

2. catalytic dehydrogenation of H_3NBD_3
reaction time: 20 min

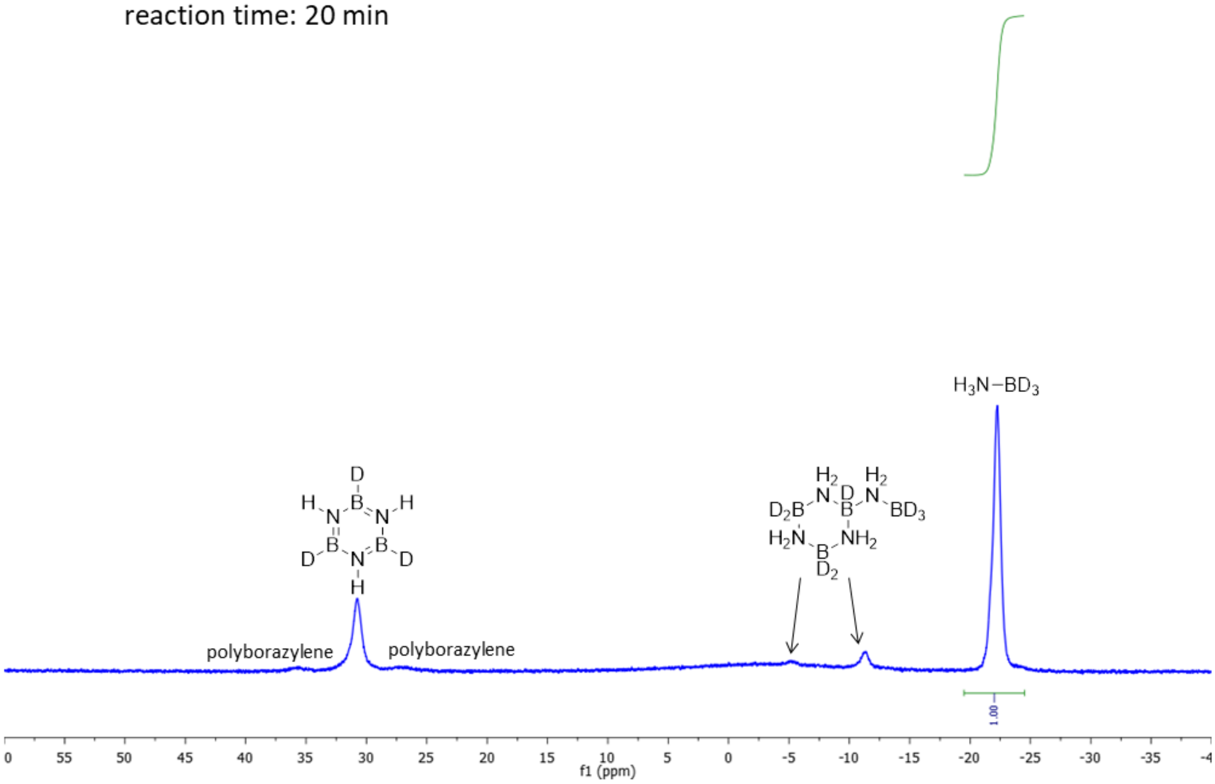

Figure SI 76: ^{11}B NMR of the second catalytic dehydrogenation of H_3NBD_3 after 20 min (128 MHz, THF with benzene-d_6 glass capillary). Formal charges are omitted for clarity.

1. catalytic dehydrogenation of H_3NBD_3
reaction time: 30 min

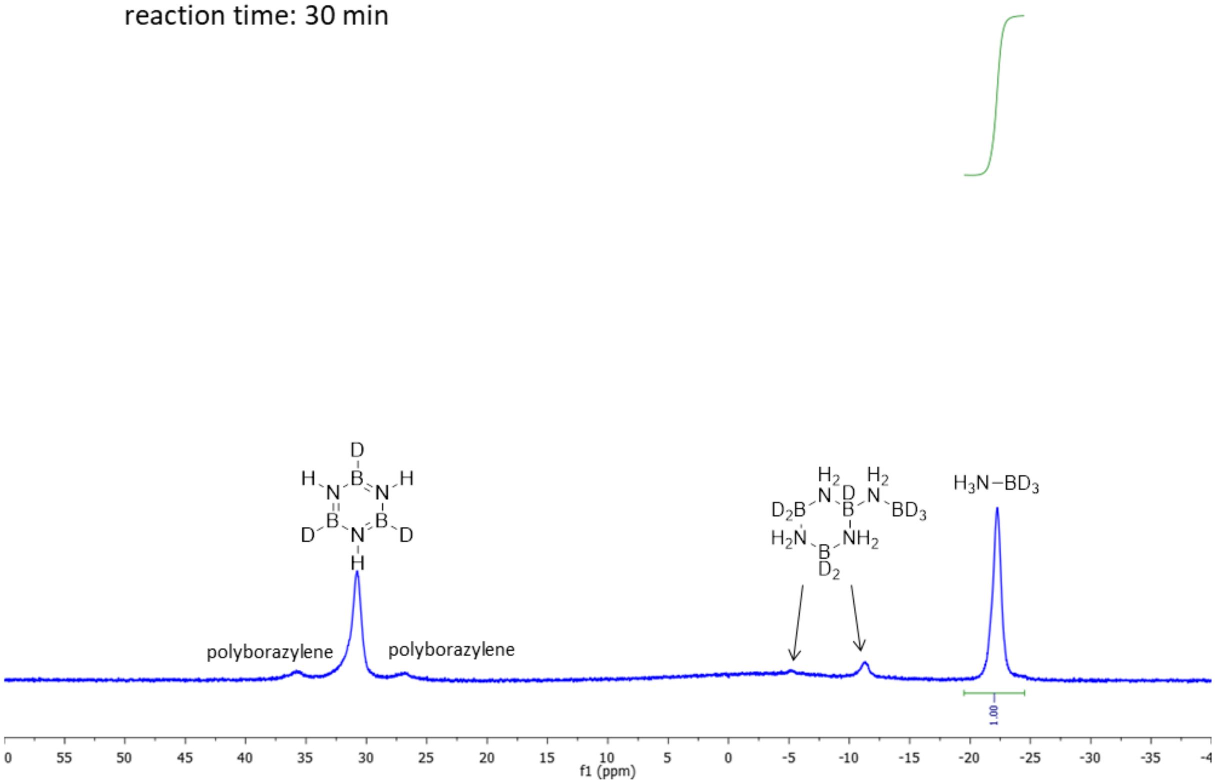

Figure SI 77: ^{11}B NMR of the first catalytic dehydrogenation of H_3NBD_3 after 30 min (128 MHz, THF with benzene-d_6 glass capillary). Formal charges are omitted for clarity.

2. catalytic dehydrogenation of H_3NBD_3
reaction time: 30 min

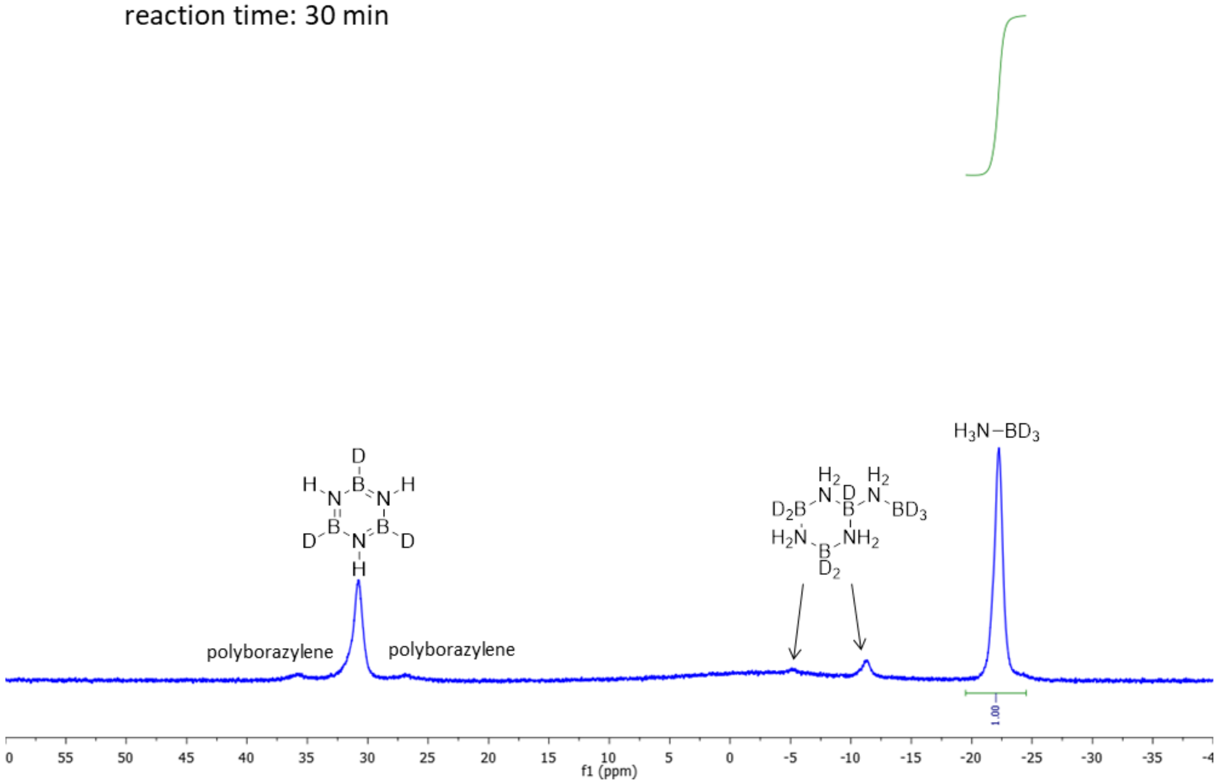

Figure SI 78: ^{11}B NMR of the second catalytic dehydrogenation of H_3NBD_3 after 30 min (128 MHz, THF with benzene-d_6 glass capillary). Formal charges are omitted for clarity.

1. catalytic dehydrogenation of H_3NBD_3
   reaction time: 40 min

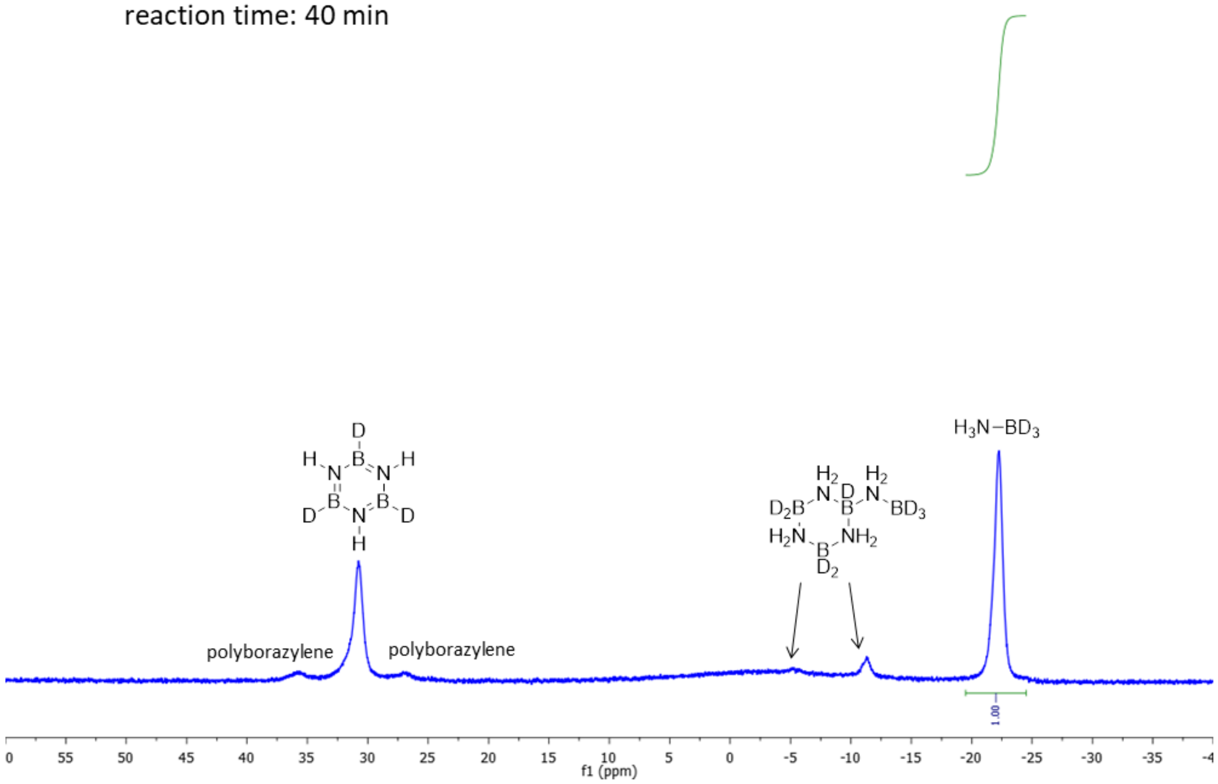

Figure SI 79: ^{11}B NMR of the first catalytic dehydrogenation of H_3NBD_3 after 40 min (128 MHz, THF with benzene-d_6 glass capillary). Formal charges are omitted for clarity.

2. catalytic dehydrogenation of H_3NBD_3
reaction time: 40 min

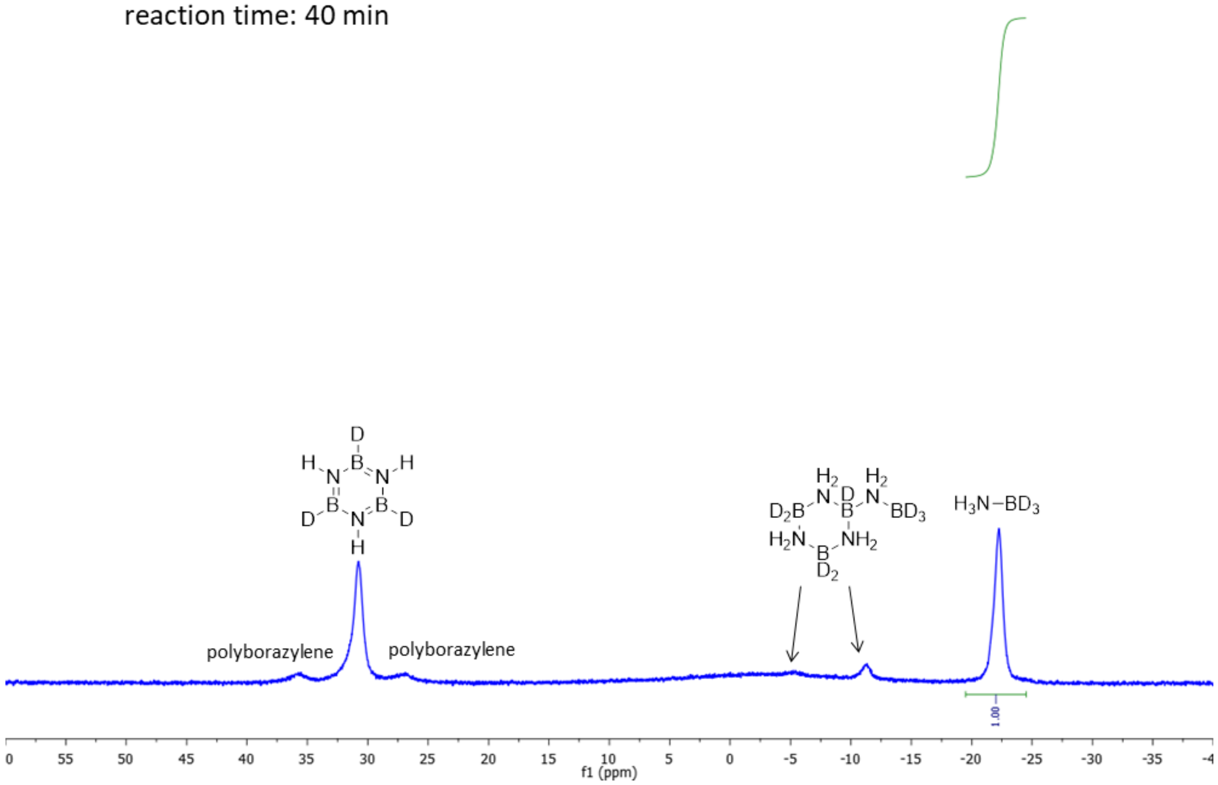

Figure SI 80: ^{11}B NMR of the second catalytic dehydrogenation of H_3NBD_3 after 40 min (128 MHz, THF with benzene-d_6 glass capillary). Formal charges are omitted for clarity.

1. catalytic dehydrogenation of D_3NBH_3
reaction time: 10 min

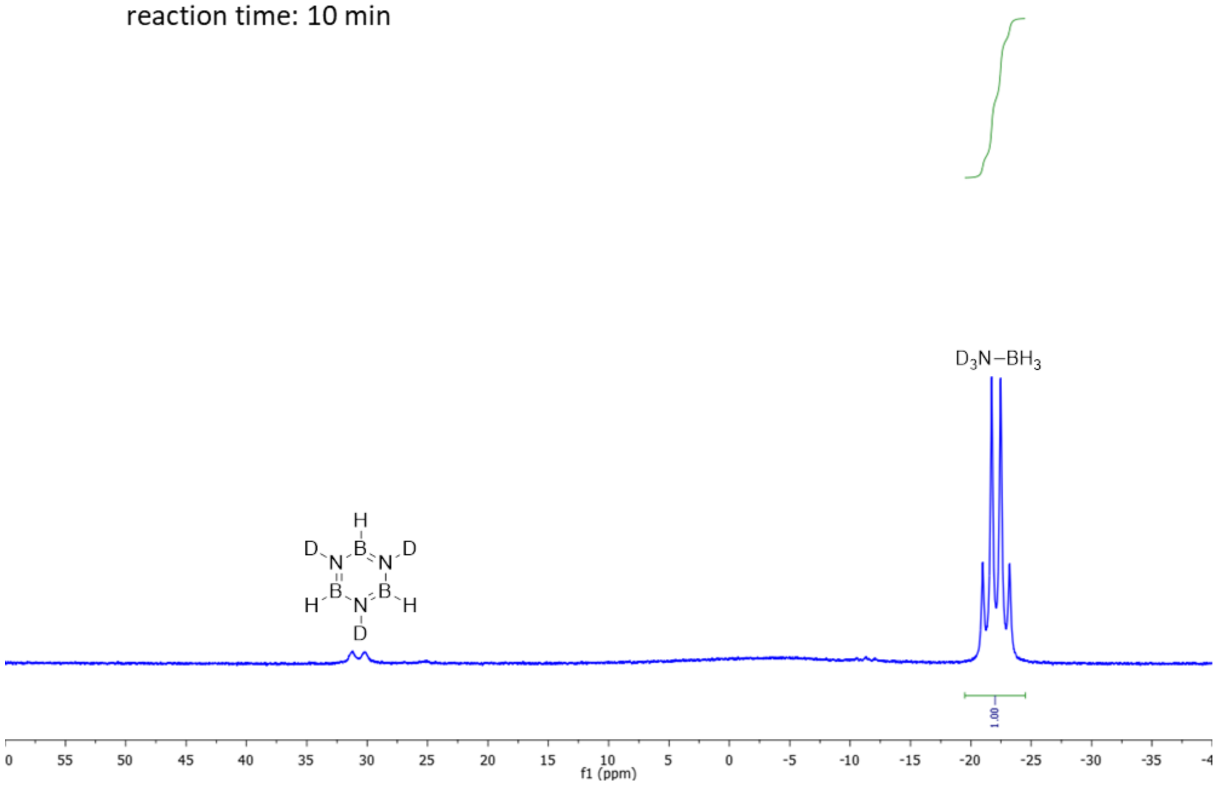

Figure SI 81: ^{11}B NMR of the first catalytic dehydrogenation of D_3NBH_3 after 10 min (128 MHz, THF with benzene-d_6 glass capillary). Formal charges are omitted for clarity.

2. catalytic dehydrogenation of D_3NBH_3
reaction time: 10 min

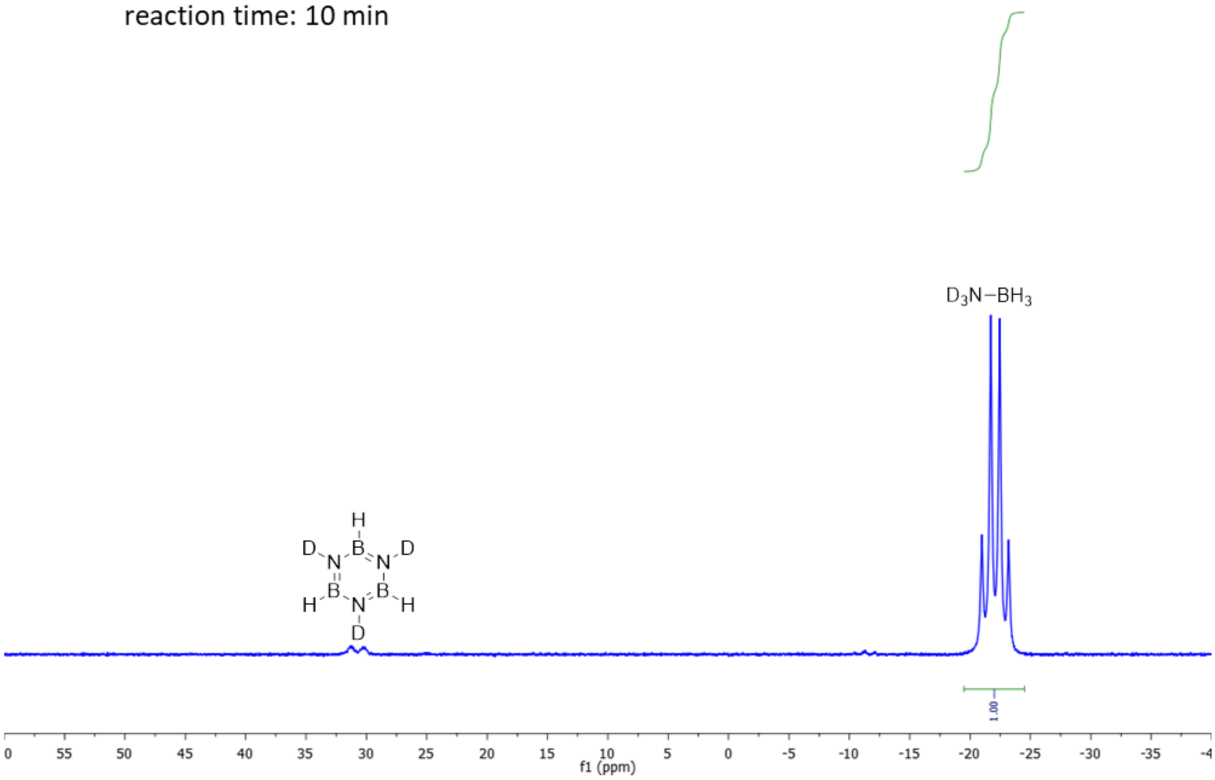

Figure SI 82: ^{11}B NMR of the second catalytic dehydrogenation of D_3NBH_3 after 10 min (128 MHz, THF with benzene-d_6 glass capillary). Formal charges are omitted for clarity.

1. catalytic dehydrogenation of D_3NBH_3
reaction time: 20 min

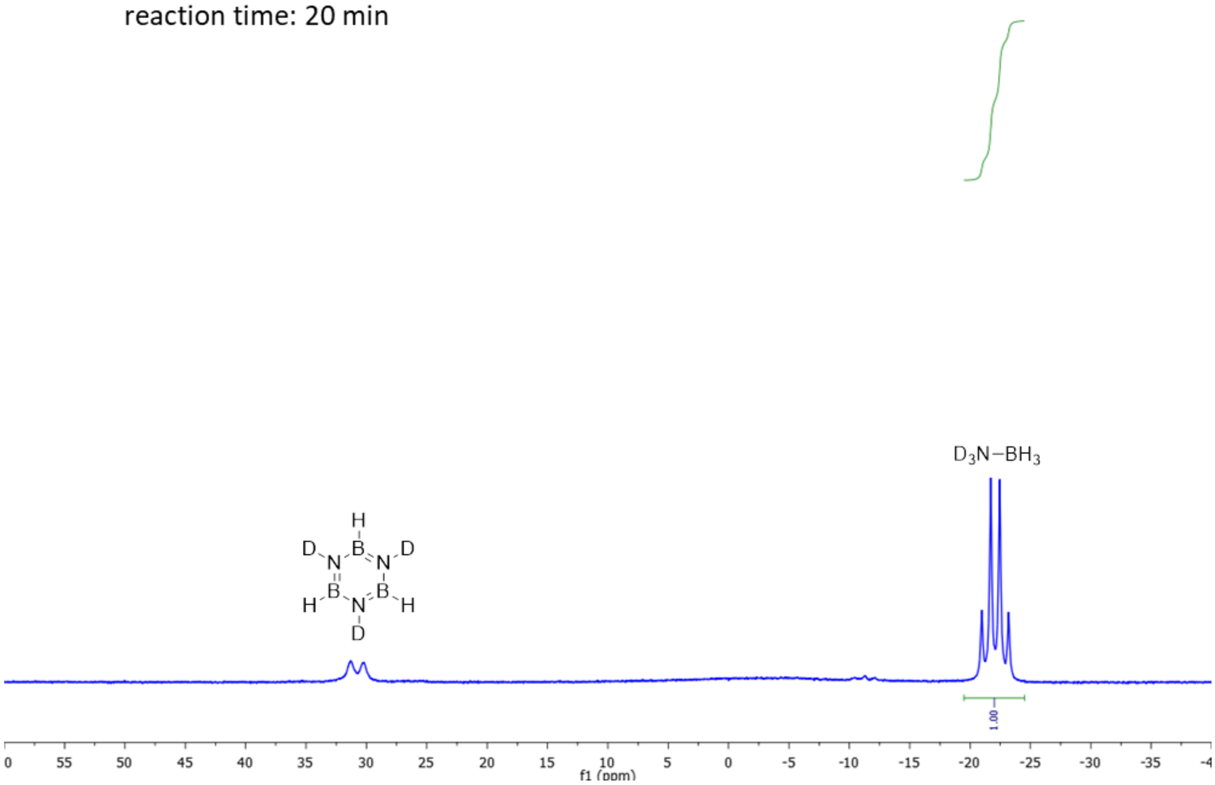

Figure SI 83: ^{11}B NMR of the first catalytic dehydrogenation of D_3NBH_3 after 20 min (128 MHz, THF with benzene-d_6 glass capillary). Formal charges are omitted for clarity.

2. catalytic dehydrogenation of D_3NBH_3
reaction time: 20 min

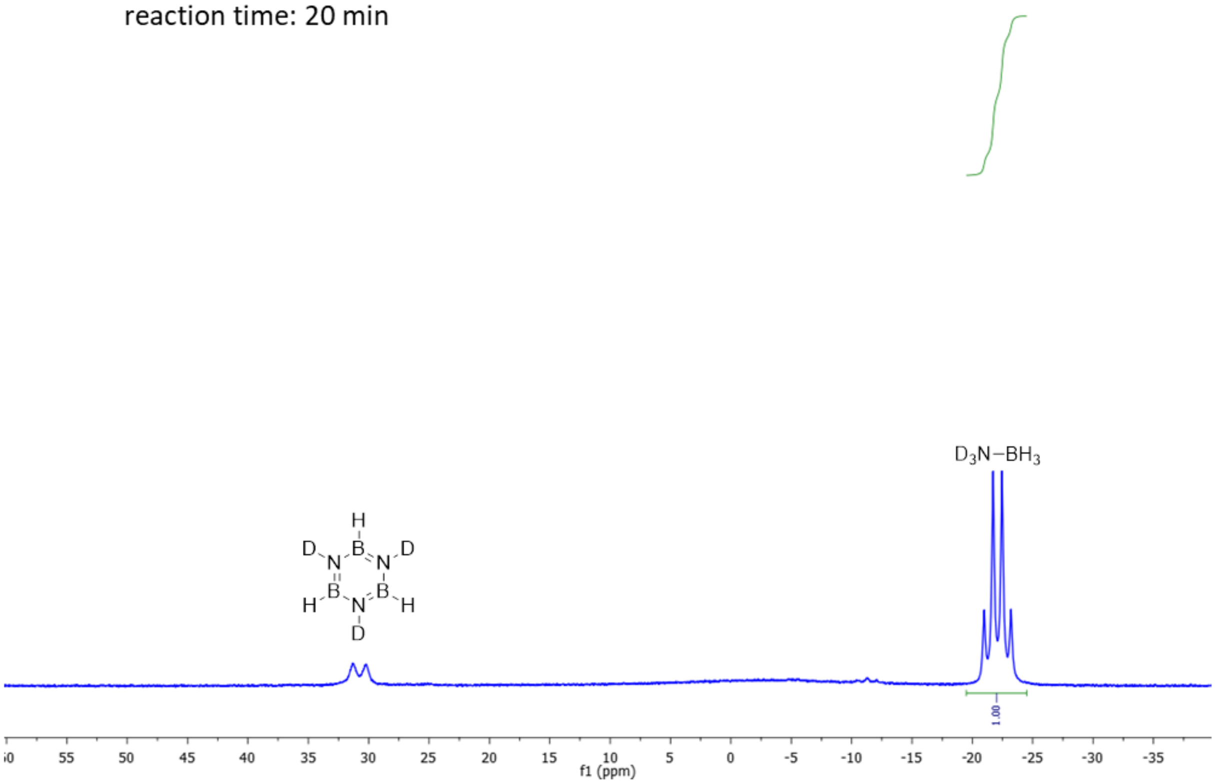

Figure SI 84: ^{11}B NMR of the second catalytic dehydrogenation of D_3NBH_3 after 20 min (128 MHz, THF with benzene-d_6 glass capillary). Formal charges are omitted for clarity.

1. catalytic dehydrogenation of D_3NBH_3
reaction time: 30 min

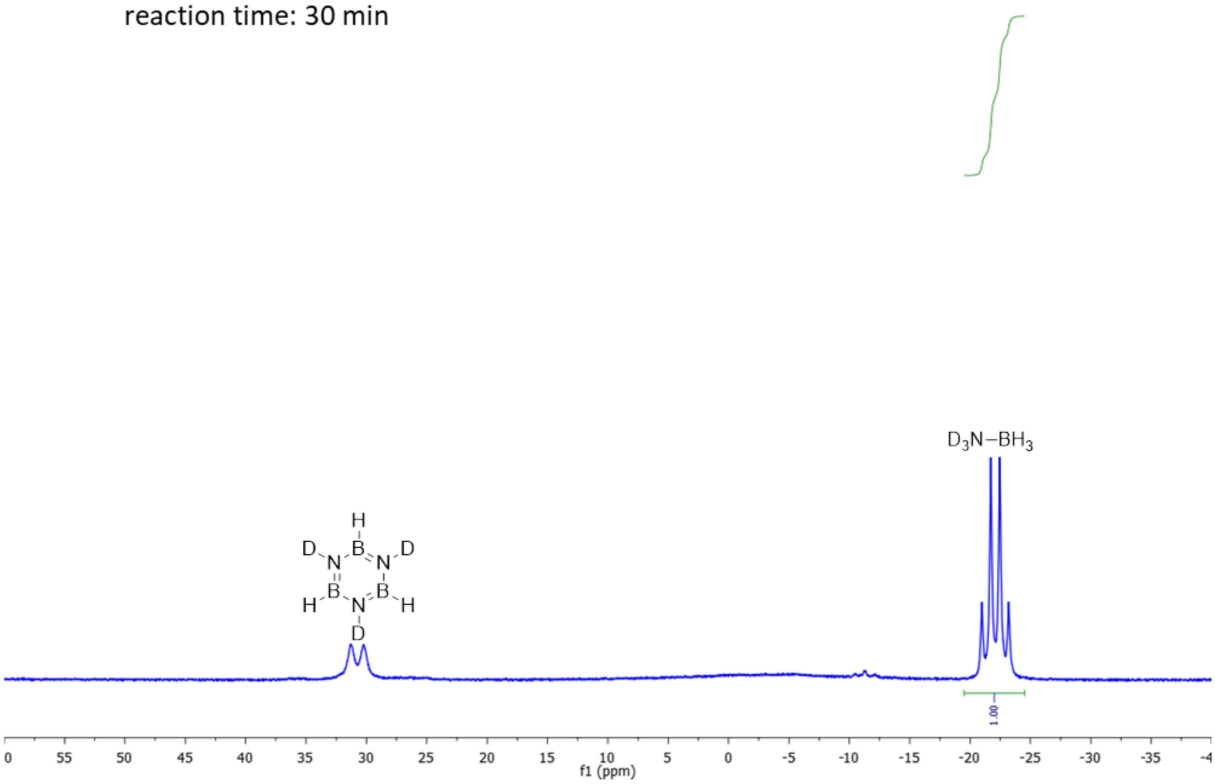

Figure SI 85: ^{11}B NMR of the first catalytic dehydrogenation of D_3NBH_3 after 30 min (128 MHz, THF with benzene-d_6 glass capillary). Formal charges are omitted for clarity.

2. catalytic dehydrogenation of D_3NBH_3
reaction time: 30 min

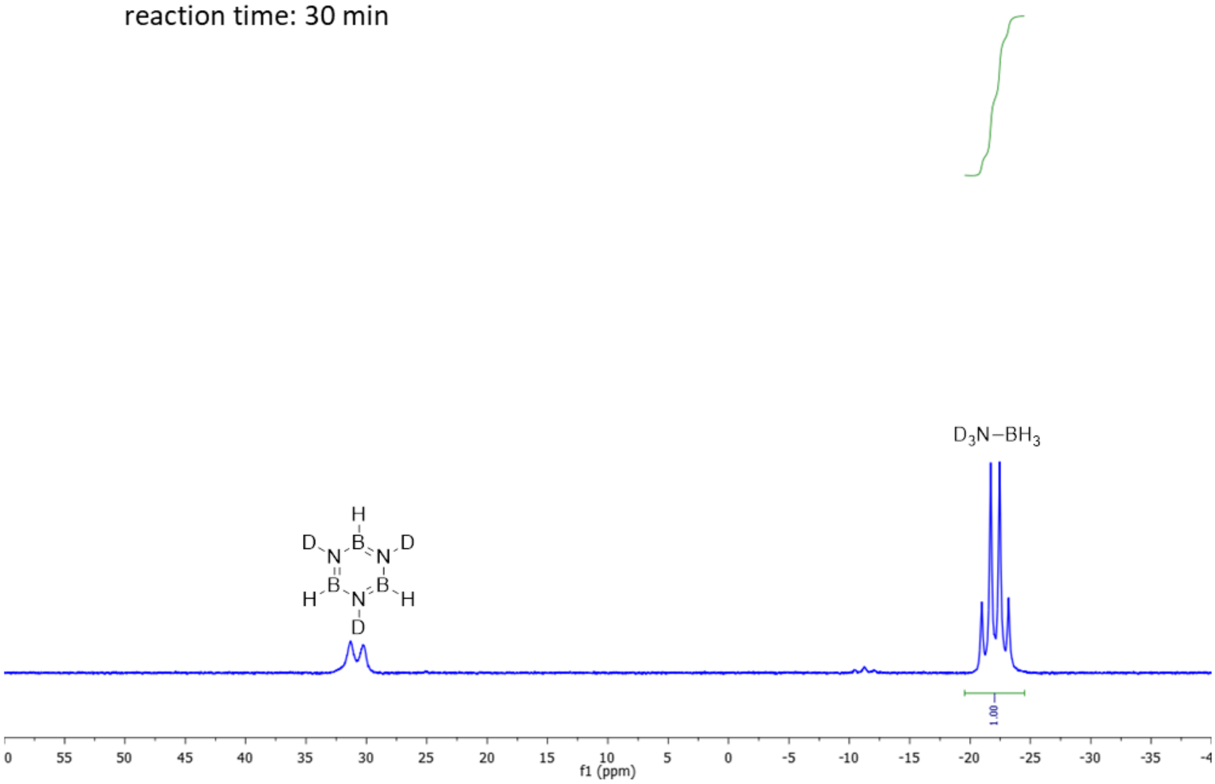

Figure SI 86: ^{11}B NMR of the second catalytic dehydrogenation of D_3NBH_3 after 30 min (128 MHz, THF with benzene-d_6 glass capillary). Formal charges are omitted for clarity.

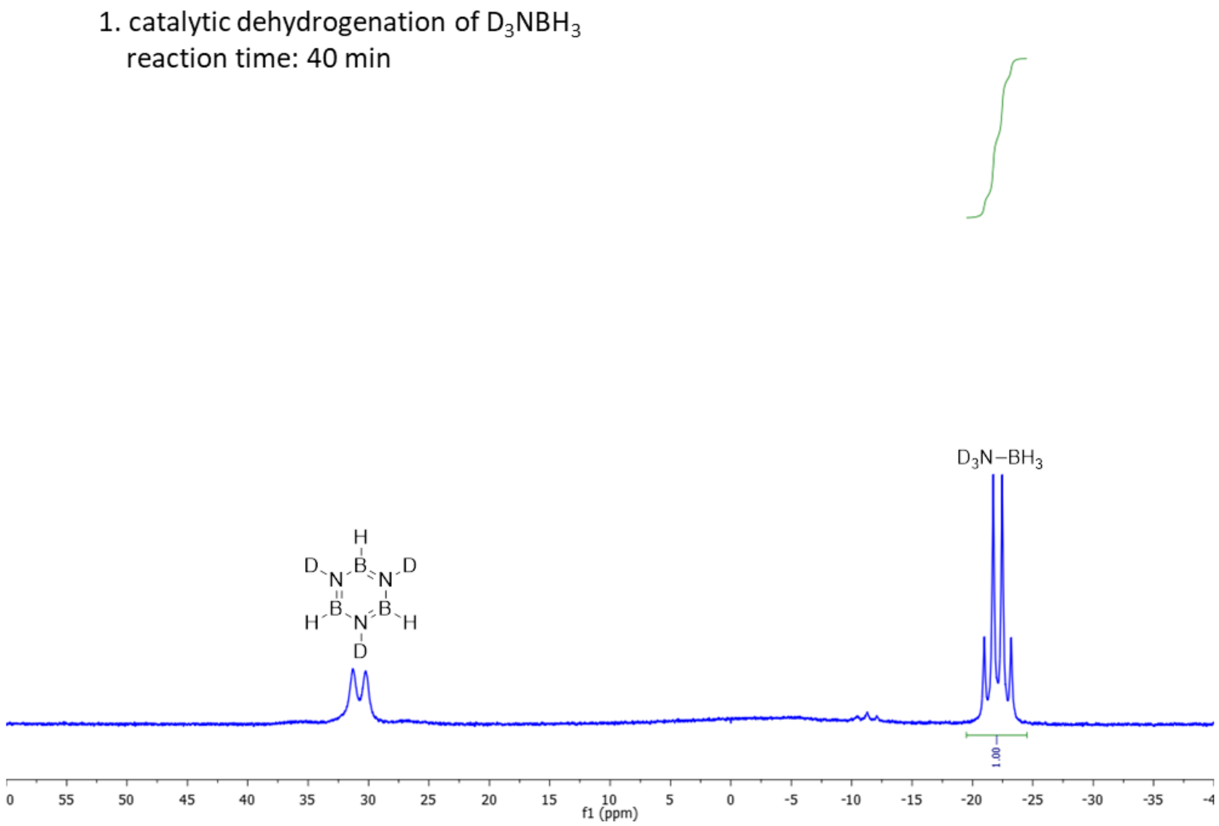

Figure SI 87: ^{11}B NMR of the first catalytic dehydrogenation of D_3NBH_3 after 40 min (128 MHz, THF with benzene-d_6 glass capillary). Formal charges are omitted for clarity.

2. catalytic dehydrogenation of D_3NBH_3
reaction time: 40 min

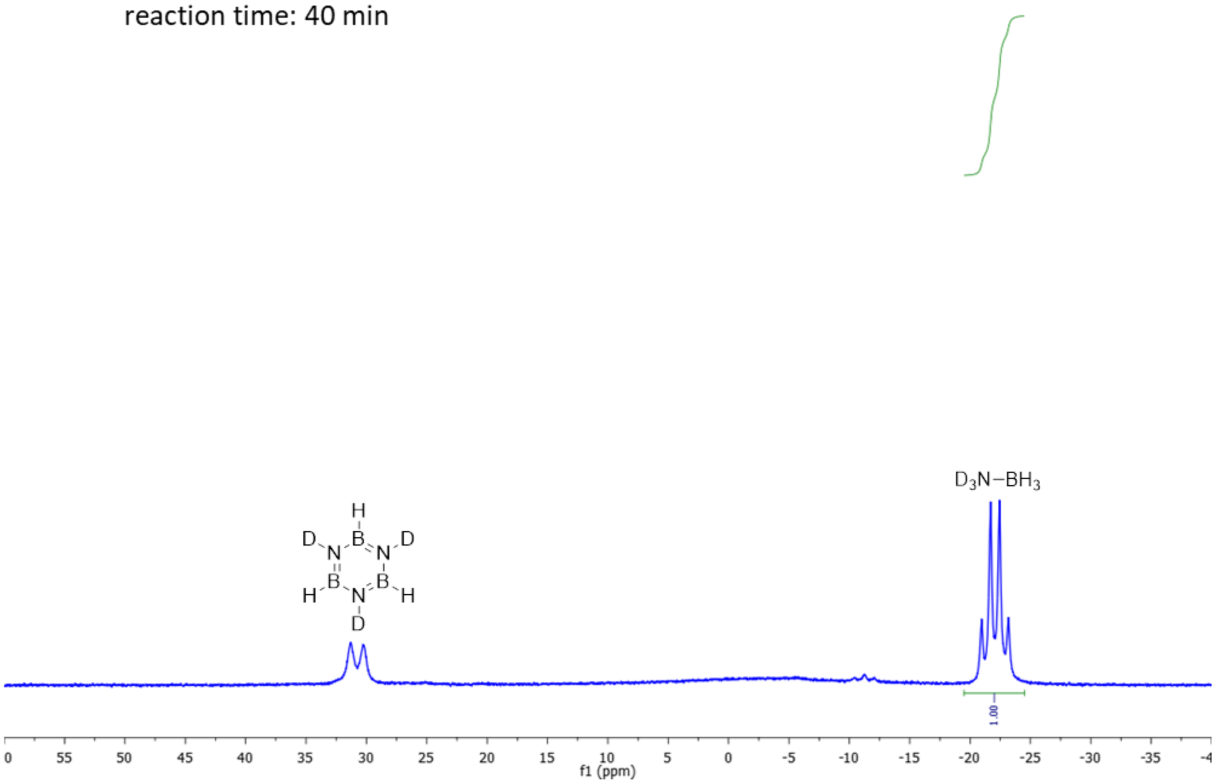

Figure SI 88: ^{11}B NMR of the second catalytic dehydrogenation of D_3NBH_3 after 40 min (128 MHz, THF with benzene-d_6 glass capillary). Formal charges are omitted for clarity.

17 Computational details

All structures were fully optimized with the hybrid version of the Perdew-Burke-Ernzerhof functional containing 25% exact HF-exchange (PBE0).[13] Dispersion interactions were taken into account with Grimme's D3 correction in conjunction with Becke-Johnson damping.[14] Solvent effects were taken into account implicitly using the SMD model for THF.[15] Thermodynamic properties were obtained at the same level of theory from a frequency computation. The computed free energies were corrected regarding the standard state by adding RT ln(c0s/c0g) (i.e., about 1.89 kcal/mol) to energies of all structures. Single-Point energies were computed at the DLPNO-CCSD(T) level with TightPNO settings.[16]

For structure optimizations and NMR calculations Gaussian16 B.01 and for DLPNO-CCSD(T) calculations ORCA program packages were used.[17]

All structure optimization and frequency computations were done with Ahlrichs triple zeta def2-TZVP and single point computations with the quadruple zeta def2-QZVPP basis set.[18]

17.1 Cartesian coordinates and energies

4_2

C	3.252932	-1.119130	0.293267
C	3.844797	-2.348981	0.393140
C	3.071255	-3.503182	0.189459
C	1.741702	-3.410732	-0.095843
C	1.115152	-2.145250	-0.187900
N	1.926547	-1.065965	0.006414
S	-0.524597	-1.972049	-0.514003
C	3.982403	0.196110	0.475436
C	3.941637	0.978100	-0.842924
H	4.457559	0.426027	-1.632183
H	4.443042	1.939694	-0.712772
H	2.924190	1.178955	-1.178387
C	5.442758	-0.035087	0.849574
H	5.982906	-0.578574	0.071027
H	5.540104	-0.581340	1.790666
H	5.931197	0.933030	0.975401
C	3.317628	0.997509	1.600568
H	2.284052	1.260712	1.369853
H	3.865270	1.930484	1.752098
H	3.336262	0.434492	2.537029
H	4.895314	-2.428208	0.623205
H	3.540237	-4.478381	0.263193
H	1.123719	-4.283472	-0.252560
S	0.524597	1.972049	-0.514003
C	-1.115152	2.145250	-0.187900
N	-1.926547	1.065965	0.006414
C	-3.252932	1.119130	0.293267
C	-3.844797	2.348981	0.393140
C	-3.071255	3.503182	0.189459
C	-1.741702	3.410732	-0.095843
H	-1.123719	4.283472	-0.252560
H	-4.895314	2.428208	0.623205
H	-3.540237	4.478381	0.263193
C	-3.982403	-0.196110	0.475436
C	-3.317628	-0.997509	1.600568
H	-2.284052	-1.260712	1.369853
H	-3.865270	-1.930484	1.752098
H	-3.336262	-0.434492	2.537029
C	-5.442758	0.035087	0.849574
H	-5.540104	0.581340	1.790666
H	-5.931197	-0.933030	0.975401
H	-5.982906	0.578574	0.071027
C	-3.941637	-0.978100	-0.842924
H	-2.924190	-1.178955	-1.178387
H	-4.457559	-0.426027	-1.632183
H	-4.443042	-1.939694	-0.712772
H	-1.460660	0.150997	-0.094839
H	1.460660	-0.150997	-0.094839

	Hatree	kcal/mol
E PBE0-D3BJ/def2TZVP	-1606.621276	-1008170.113831
zpv	0.410736	257.740742
H	0.435416	273.227676
G	0.356948	223.988261
E SMD-PBE0-D3BJ/def2TZVP	-1606.645857	-1008185.538403
E DLPNO-CCSD(T)/def2-TZVP	-1605.387951	-1007396.190458

3_2

C	-3.898337	1.851217	0.000000
C	-2.538297	1.922465	0.000000
C	-4.479363	0.569966	0.000000
C	-3.698368	-0.551119	0.000000
C	-2.282875	-0.459155	0.000000
N	-1.788148	0.808171	0.000000
S	-1.267148	-1.799846	0.000000
H	-4.495792	2.751352	0.000000
H	-1.981885	2.851506	0.000000
H	-5.559038	0.469554	0.000000
H	-4.129861	-1.542542	0.000000
N	1.788148	-0.808171	0.000000
C	2.282875	0.459155	0.000000
C	3.698368	0.551119	0.000000
C	4.479363	-0.569966	0.000000
C	3.898337	-1.851217	0.000000
C	2.538297	-1.922465	0.000000
S	1.267148	1.799846	0.000000
H	-0.756831	0.943516	0.000000
H	0.756831	-0.943516	0.000000
H	1.981885	-2.851506	0.000000
H	4.495792	-2.751352	0.000000
H	5.559038	-0.469554	0.000000
H	4.129861	1.542542	0.000000

	Hatree	kcal/mol
E PBE0-D3BJ/def2TZVP	-1292.359702	-810967.990441
zpv	0.185408	116.345281
H	0.199087	124.928984
G	0.142125	89.184788
E SMD-PBE0-D3BJ/def2TZVP	-1292.383408	-810982.866005
E DLPNO-CCSD(T)/def2-TZVP	-1291.358338	-810339.625242

$TS_{42/4SH2}$

C	-2.713836	0.115915	0.381372
C	-2.799654	-1.259607	1.020184
C	-3.67943	1.088261	0.563907
C	-3.841604	-1.282124	2.136112
C	-1.441152	-1.633521	1.619302
C	-3.207818	-2.285538	-0.044079
N	-1.643237	0.382959	-0.391308
C	-3.531294	2.327672	-0.050649
H	-4.540374	0.891063	1.184349
H	-4.854937	-1.126791	1.758853
H	-3.634301	-0.530116	2.901489
H	-3.822577	-2.262494	2.616044
H	-1.518468	-2.605081	2.112760
H	-1.126243	-0.897032	2.362846
H	-0.667666	-1.706396	0.856321
H	-4.162594	-2.013537	-0.500835
H	-3.321488	-3.266948	0.423627
H	-2.453568	-2.375058	-0.825990
C	-1.448324	1.591775	-0.934070
C	-2.406518	2.596781	-0.791704
H	-4.290794	3.090279	0.080289
S	0.017335	1.910555	-1.802389
H	-2.231197	3.565615	-1.240279
H	0.777404	0.892885	-1.146029
N	1.625884	-0.325710	-0.409357
C	1.362636	-1.474020	-1.046012
C	2.734396	-0.187495	0.333686
C	2.276779	-2.536475	-1.015318
S	-0.143229	-1.642530	-1.890389
C	2.911492	1.140132	1.056969
C	3.662477	-1.211091	0.417301
C	3.430841	-2.390222	-0.286081
H	2.044062	-3.450658	-1.545258
H	-0.942515	-0.554663	-0.980116
C	4.014732	1.066661	2.108633
C	1.603379	1.520543	1.753899
C	3.28914	2.217221	0.034757
H	4.552673	-1.103638	1.019146
H	4.152257	-3.198973	-0.240086
H	4.996473	0.893078	1.662209
H	3.822895	0.278402	2.840757
H	4.064712	2.017411	2.644044
H	1.726635	2.472108	2.277268
H	1.319435	0.761323	2.487246
H	0.786535	1.626160	1.041604
H	4.212121	1.951573	-0.486324
H	3.446564	3.172783	0.542471
H	2.503279	2.360364	-0.706562

	Hatree	kcal/mol
E PBE0-D3BJ/def2TZVP	-1606.592535	-1008152.078461

zpv	0.399824	250.893358
H	0.424147	266.156272
G	0.347291	217.928402
E SMD-PBE0-D3BJ/def2TZVP	-1606.613830	-1008165.441131
E TightPNO-DLPNO-CCSD(T)/def2-QZVPP	-1605.356461	-1007376.430216

$TS_{32/3SH2}$

C	-2.397924	-1.481658	0.516759
C	-3.713376	-1.230366	0.824603
N	-1.592238	-0.543319	0.022349
C	-4.194686	0.057703	0.594578
H	-4.344586	-2.011858	1.224943
C	-2.021532	0.705322	-0.188710
C	-3.355904	1.026619	0.093461
H	-5.227229	0.302145	0.816562
S	-0.937491	1.898691	-0.814511
H	-3.700151	2.038351	-0.077377
H	0.258143	1.199478	-0.459932
N	1.614133	0.569032	0.044817
C	2.007355	-0.68758	-0.203345
C	2.470505	1.454861	0.548063
C	3.337023	-1.063722	0.052143
S	0.876692	-1.828068	-0.846853
C	3.785271	1.157043	0.829686
C	4.218924	-0.140595	0.563781
H	3.64104	-2.081908	-0.153397
H	-0.443196	-1.016601	-0.393039
H	4.448011	1.908931	1.236919
H	5.245843	-0.426532	0.763230
H	-1.939184	-2.457217	0.646619
H	2.066904	2.449484	0.721025

	Hatree	kcal/mol
E PBE0-D3BJ/def2TZVP	-1292.332327	-810950.812193
zpv	0.175281	109.990493
H	0.188507	118.289933
G	0.134019	84.098196
E SMD-PBE0-D3BJ/def2TZVP	-1292.352592	-810963.528792
E TightPNO-DLPNO-CCSD(T)/def2-QZVPP	-1291.327773	-810320.445211

$4SH_2$

C	-2.762309	0.529449	0.462349
C	-3.685011	1.562168	0.564606
C	-3.468432	2.733681	-0.148067
C	-2.334175	2.863739	-0.918516
C	-1.454719	1.782017	-0.961898
N	-1.678373	0.646916	-0.313591

S	0.013813	1.933612	-1.914736
C	-2.925914	-0.795753	1.191465
C	-3.327820	-1.864490	0.169959
H	-4.265417	-1.599189	-0.325142
H	-3.467186	-2.825313	0.672612
H	-2.559002	-1.993621	-0.591524
C	-4.002347	-0.722160	2.269847
H	-4.995513	-0.548175	1.848860
H	-3.791487	0.065233	2.997916
H	-4.039459	-1.672577	2.806732
C	-1.598442	-1.181523	1.845943
H	-0.794849	-1.234757	1.112793
H	-1.695214	-2.155929	2.331295
H	-1.312691	-0.448722	2.605042
H	-4.559441	1.462623	1.191084
H	-4.178836	3.550358	-0.082855
H	-2.111624	3.774604	-1.459539
S	-0.013813	-1.933612	-1.914736
C	1.454719	-1.782017	-0.961898
N	1.678373	-0.646916	-0.313591
C	2.762309	-0.529449	0.462349
C	3.685011	-1.562168	0.564606
C	3.468432	-2.733681	-0.148067
C	2.334175	-2.863739	-0.918516
H	2.111624	-3.774604	-1.459539
H	4.559441	-1.462623	1.191084
H	4.178836	-3.550358	-0.082855
C	2.925914	0.795753	1.191465
C	1.598442	1.181523	1.845943
H	0.794849	1.234757	1.112793
H	1.695214	2.155929	2.331295
H	1.312691	0.448722	2.605042
C	4.002347	0.722160	2.269847
H	3.791487	-0.065233	2.997916
H	4.039459	1.672577	2.806732
H	4.995513	0.548175	1.848860
C	3.327820	1.864490	0.169959
H	2.559002	1.993621	-0.591524
H	4.265417	1.599189	-0.325142
H	3.467186	2.825313	0.672612
H	0.656788	0.898214	-1.312650
H	-0.656788	-0.898214	-1.312650

	Hatree	kcal/mol
E PBE0-D3BJ/def2TZVP	-1606.600525	-1008157.092412
zpv	0.40252	252.585124
H	0.427882	268.500020
G	0.348068	218.415977
E SMD-PBE0-D3BJ/def2TZVP	-1606.619574	-1008169.045590
E TightPNO-DLPNO-CCSD(T)/def2-QZVPP	-1605.372061	-1007386.219097

$3SH_2$

C	3.969202	-1.268387	0.582255
C	2.612439	-1.478496	0.431944
C	4.452089	0.015641	0.358851
C	3.573375	1.018375	0.008840
C	2.216006	0.707442	-0.118756
N	1.754814	-0.523101	0.085065
S	1.097335	1.972772	-0.572072
H	4.625341	-2.081559	0.863714
H	2.179754	-2.462669	0.592417
H	5.509085	0.233033	0.463249
H	3.913756	2.032276	-0.160845
N	-1.754789	0.523097	0.085067
C	-2.215995	-0.707456	-0.118663
C	-3.573364	-1.018368	0.008978
C	-4.452063	-0.015602	0.358941
C	-3.969160	1.268435	0.582254
C	-2.612398	1.478522	0.431901
S	-1.097340	-1.972821	-0.571924
H	0.044199	-1.243174	-0.336741
H	-0.044191	1.243130	-0.336803
H	-2.179701	2.462702	0.592302
H	-4.625285	2.081631	0.863674
H	-5.509058	-0.232979	0.463374
H	-3.913759	-2.032276	-0.160635

	Hatree	kcal/mol
E PBE0-D3BJ/def2TZVP	-1292.336268	-810953.285365
zpv	0.177954	111.667826
H	0.192033	120.502532
G	0.134526	84.416343
E SMD-PBE0-D3BJ/def2TZVP	-1292.354061	-810964.450641
E TightPNO-DLPNO-CCSD(T)/def2-QZVPP	-1291.338562	-810327.215242

4SH

N	0.439422	-0.494923	-0.004443
C	1.665735	-0.004693	0.001561
C	1.945127	1.362033	0.008710
C	0.871635	2.226092	0.009320
C	-0.421336	1.719260	0.002965
C	-0.601981	0.342495	-0.003785
S	3.013550	-1.138320	0.000794
C	-1.968057	-0.325258	-0.011442
H	-1.268010	2.390506	0.003374

H	1.037076	3.297721	0.014662
H	2.966388	1.721700	0.013510
C	-2.079703	-1.209834	1.232826
C	-3.106755	0.686714	-0.010586
C	-2.071263	-1.198264	-1.264571
H	-1.263510	-1.930297	-1.287549
H	-3.027019	-1.728686	-1.274297
H	-2.008945	-0.590866	-2.171336
H	-2.024245	-0.610752	2.145547
H	-3.035241	-1.740761	1.230880
H	-1.271738	-1.941641	1.254762
H	-4.062522	0.157988	-0.016348
H	-3.080175	1.329248	-0.894369
H	-3.086230	1.320960	0.879308
H	2.225585	-2.223903	-0.006643

	Hatree	kcal/mol
E PBE0-D3BJ/def2TZVP	-803.2927997	-504073.863088
zpv	0.200422	125.766709
H	0.21283	133.552847
G	0.163504	102.600313
E SMD-PBE0-D3BJ/def2TZVP	-803.303765	-504080.744122
E TightPNO-DLPNO-CCSD(T)/def2-QZVPP	-802.679995	-503689.322481

3SH

N	0.494067	-0.520507	-0.000027
C	1.705447	0.018347	0.000057
C	1.931233	1.395578	-0.000050
C	0.834640	2.233508	-0.000049
C	-0.439903	1.683042	0.000006
C	-0.549605	0.303640	-0.000006
S	3.092826	-1.063311	-0.000310
H	-1.325254	2.305703	0.000031
H	0.972929	3.308755	-0.000067
H	2.940498	1.788655	-0.000132
H	2.343892	-2.176474	-0.000488
H	-1.525911	-0.172987	-0.000004

	Hatree	kcal/mol
E PBE0-D3BJ/def2TZVP	-646.1603807	-405471.777409
zpv	0.08828	55.396539
H	0.095131	59.695606
G	0.058235	36.543016
E SMD-PBE0-D3BJ/def2TZVP	-646.170228	-405477.956956
E TightPNO-DLPNO-CCSD(T)/def2-QZVPP	-645.663863	-405160.207782

NMe_2BH_2

H	-0.00007900	2.11259500	1.04830900
H	-0.00007900	2.11259500	-1.04830900
N	0.00002600	0.14903900	0.00000000
B	0.00000400	1.53376700	0.00000000
C	0.00000400	-0.64737600	1.20598800
H	-0.88432000	-1.29374200	1.24645600
H	0.88429300	-1.29378900	1.24641700
H	-0.00001500	0.00314100	2.07850600
C	0.00000400	-0.64737600	-1.20598800
H	0.88429300	-1.29378900	-1.24641700
H	-0.88432000	-1.29374200	-1.24645600
H	-0.00001500	0.00314100	-2.07850600

	Hartree	kcal/mol
E PBE0-D3BJ/def2TZVP	-160.507448	-100719.948
zpv	0.104834	65.7843309
H	0.111145	69.7445434
G	0.077606	48.6985023
E PBE0-D3BJ/def2TZVP SMD (THF)	-160.510226	-100721.692
E TightPNO-DLPNO-CCSD(T)/def2-QZVPP	-160.397578	-100651.004

$(NMe_2BH_2)_2$

H	-0.00002100	-1.21563900	1.61334900
H	-0.00012700	0.80212900	1.85735700
H	0.00010000	-1.21565300	-1.61332000
H	0.00004900	0.80208800	-1.85741500
N	-1.16469500	-0.02459600	-0.00003700
N	1.16466300	-0.02450500	0.00003100
B	-0.00003600	-0.12987900	1.08864600
B	0.00002400	-0.12986900	-1.08865000
C	-2.12287700	-1.12774000	0.00001700
H	-2.75552400	-1.07841100	0.89081600
H	-2.75666100	-1.07743600	-0.88991800
H	-1.58730700	-2.07384900	-0.00086100
C	-1.88251300	1.24989000	-0.00002600
H	-2.50959400	1.32930300	-0.89224300
H	-2.51023700	1.32887300	0.89177100
H	-1.17380700	2.07509700	0.00044600
C	2.12263300	-1.12795000	-0.00001100
H	2.75532400	-1.07869200	-0.89078000
H	2.75636000	-1.07777200	0.88996700
H	1.58681600	-2.07390200	0.00079000
C	1.88278300	1.24972800	0.00002700
H	2.50993700	1.32898800	0.89221100
H	2.51050500	1.32859800	-0.89178100
H	1.17431800	2.07515800	-0.00037500

	Hartree	kcal/mol

E PBE0-D3BJ/def2TZVP	-321.0522221	-201463.319
zpv	0.215758	135.390195
H	0.226525	142.146589
G	0.182156	114.30462
E TightPNO-DLPNO-CCSD(T)/def2-QZVPP	-320.8310746	-201324.547

5$_{Me2}$

C	1.71285800	2.70084900	0.13097600
C	0.34453600	2.79015500	0.04863200
C	2.32767500	1.45192700	0.10915100
C	-0.39797200	1.60566900	-0.04648500
C	1.53376900	0.32215700	0.01359300
H	3.40351400	1.37374500	0.16599300
H	2.31416500	3.60071400	0.20169200
H	-0.16710000	3.74389500	0.04329700
N	0.19945300	0.41265500	-0.05081500
S	-2.14166900	1.71990000	-0.11479300
C	2.09795800	-1.09149400	-0.02411700
C	1.66871200	-1.75468200	-1.33503100
H	2.01284300	-2.79191300	-1.36432700
H	0.58403100	-1.74347500	-1.44465000
H	2.09347200	-1.22908500	-2.19356700
C	3.61994400	-1.10650500	0.05720300
H	3.98086000	-0.66502000	0.98931200
H	3.97633200	-2.13840400	0.01967600
H	4.07432100	-0.56937700	-0.77853200
C	1.54124100	-1.88425400	1.16123000
H	1.92860900	-2.90627600	1.14479900
H	1.82922200	-1.42442100	2.10968000
H	0.45212900	-1.93501500	1.13212700
H	-2.17921100	-0.11350800	-1.94934800
H	-3.88501600	0.02334800	-0.85567000
H	-1.11016100	-0.87476000	0.09875800
B	-2.67654700	0.04641800	-0.86015400
N	-2.11769300	-1.13777000	0.06260600
C	-2.27563000	-2.44016300	-0.58742100
H	-1.82121700	-3.22980100	0.01545100
H	-3.33913300	-2.64698200	-0.71522900
H	-1.80707100	-2.40627500	-1.56909600
C	-2.65374400	-1.14646100	1.42615900
H	-3.72436100	-1.35018800	1.38506100
H	-2.15767900	-1.91138300	2.02784000
H	-2.49751700	-0.16369700	1.86830900

	Hartree	kcal/mol
E PBE0-D3BJ/def2TZVP	-963.8410545	-604819.4182
zpv	0.312308	195.9762369
H	0.330239	207.2281098
G	0.268563	168.5258338

E PBE0-D3BJ/def2TZVP SMD (THF)	-963.860719	-604831.758
E TightPNO-DLPNO-CCSD(T)/def2-QZVPP	-963.1139961	-604363.1821

TS$_{5Me2/4+NMe2BH2}$

C	-2.43446100	2.27484700	-0.18160000
C	-1.16967000	2.77007000	-0.09398200
C	-2.67655900	0.88655300	-0.14997800
C	-0.06091700	1.89295400	0.03063800
C	-1.62008800	0.03024200	-0.03314900
H	-3.68268400	0.50392100	-0.21658900
H	-3.27302300	2.95619900	-0.27530500
H	-0.96422900	3.83113200	-0.11379800
N	-0.37243500	0.56020900	0.04693000
S	1.52835700	2.38589000	0.14698300
C	-1.72609300	-1.47843700	0.03138200
C	-1.22054700	-1.94986000	1.39980400
H	-1.26596900	-3.03994300	1.45544400
H	-0.18878300	-1.65082300	1.58611700
H	-1.83988800	-1.53968600	2.20038200
C	-3.16917900	-1.93817300	-0.13786500
H	-3.58218400	-1.62799900	-1.10017900
H	-3.20360100	-3.02858800	-0.09594900
H	-3.81204500	-1.55618100	0.65792500
C	-0.88490100	-2.09333700	-1.09123400
H	-0.94192800	-3.18290800	-1.04103400
H	-1.25249300	-1.77558700	-2.06945000
H	0.16717700	-1.81521300	-1.02255000
H	3.10434200	0.49565900	1.73401100
H	3.90352800	1.06995400	-0.10212600
H	0.43962300	-0.05055200	0.10867900
B	3.16420400	0.38315000	0.54193600
N	2.71562900	-0.85189300	-0.01713500
C	2.47703100	-2.00826400	0.81323100
H	1.66028100	-2.63006200	0.42670200
H	3.36559000	-2.65362600	0.86862000
H	2.23038800	-1.69327600	1.82738100
C	3.01440000	-1.18955100	-1.38929500
H	3.91628200	-1.81339400	-1.46699600
H	2.19440400	-1.75080600	-1.85458900
H	3.17401800	-0.27869000	-1.96437800

	Hartree	kcal/mol
E PBE0-D3BJ/def2TZVP	-963.8055939	-604797.1663
zpv	0.310527	194.8586425
H	0.328715	206.2717853
G	0.265622	166.6803284
E PBE0-D3BJ/def2TZVP SMD (THF)	-963.8272285	-604810.7422
E TightPNO-DLPNO-CCSD(T)/def2-QZVPP	-963.0764425	-604339.6169

H_2

H	-8.948268	1.360715	0.000000
H	-8.202252	1.346835	0.000000

	Hatree	kcal/mol
E PBE0-D3BJ/def2TZVP	-1.168278634	-733.105872
zpv	0.010053	6.308352
H	0.013358	8.382271
G	-0.001442	-0.904869
E SMD-PBE0-D3BJ/def2TZVP	-1.167808	-732.810626
E TightPNO-DLPNO-CCSD(T)/def2-QZVPP	-1.173897	-736.631317

5

C	0.24574200	2.75151700	0.01642400
C	-1.04325700	2.27588800	0.03398800
C	1.31259100	1.85802700	-0.00916600
C	-1.24396400	0.88987900	0.03622100
C	1.04327200	0.50018300	-0.00048600
H	2.32858500	2.22381200	-0.03514200
H	0.43203600	3.81993300	0.00853200
H	-1.89801100	2.93975900	0.02933600
N	-0.21390900	0.04103000	0.03638900
S	-2.88680600	0.28566600	0.07103400
C	2.13092500	-0.56462800	-0.02573200
C	1.91118000	-1.46445800	-1.24434100
H	2.64924500	-2.27078900	-1.25555200
H	0.91318900	-1.90414200	-1.23840800
H	2.01357200	-0.89502000	-2.17114600
C	3.52725900	0.04098600	-0.11062000
H	3.75193400	0.66722300	0.75613400
H	4.26978200	-0.75959400	-0.14106300
H	3.65200400	0.64276900	-1.01383100
C	2.04145500	-1.39825300	1.25523600
H	2.80903300	-2.17650300	1.25157500
H	2.19171900	-0.77408000	2.13941200
H	1.06906100	-1.88354700	1.35026000
H	-2.19530300	-1.50583600	-1.69228500
H	-3.81389500	-1.99234200	-0.56550600
H	-1.48079100	-3.18555700	0.07009000
H	-0.87701200	-1.62940800	0.37302100
H	-2.07648100	-2.30101200	1.33612000
B	-2.71865500	-1.48978700	-0.60453600
N	-1.71576400	-2.25135000	0.38867500

	Hartree	kcal/mol
E PBE0-D3BJ/def2TZVP	-885.29258	-555529.504
zpv	0.255898	160.578426
H	0.271052	170.087705
G	0.215659	135.328071
E PBE0-D3BJ/def2TZVP SMD (THF)	-885.314736	-555543.407

E TightPNO-DLPNO-CCSD(T)/def2-QZVPP	-884.623757	-555109.812

$TS_{5/4+NH2BH2}$

C	-0.39823000	2.91279800	0.14832400
C	0.92219300	2.59526700	0.06159400
C	-1.39660300	1.91452800	0.12512800
C	1.33818600	1.23981200	-0.05519000
C	-1.01906200	0.60859200	0.01309500
H	-2.43994200	2.17892600	0.19538000
H	-0.69452500	3.95235800	0.23796000
H	1.69618400	3.34954100	0.07958500
N	0.30644800	0.33280700	-0.07235800
S	2.91166300	0.71435500	-0.16421500
C	-1.95732000	-0.58008600	-0.02494400
C	-1.65308900	-1.50159800	1.16206800
H	-2.31491600	-2.37055000	1.13332100
H	-0.62327400	-1.86349200	1.16128400
H	-1.81657800	-0.98047300	2.10768600
C	-3.41203500	-0.13547100	0.06516700
H	-3.68857800	0.51268500	-0.76913100
H	-4.05930700	-1.01422300	0.03352400
H	-3.61445300	0.39297400	0.99906800
C	-1.75954400	-1.33555800	-1.34429000
H	-2.42336100	-2.20281000	-1.37601600
H	-1.99315500	-0.69502600	-2.19739500
H	-0.73533100	-1.69244800	-1.46854400
H	1.65864000	-2.77739000	1.83437300
H	3.40854000	-2.55252900	0.70875200
H	0.55643400	-3.35838400	-0.36273200
H	0.61336400	-0.63045800	-0.15380000
H	1.94090600	-3.07944200	-1.28113600
B	2.24047700	-2.77106300	0.78604500
N	1.51546400	-3.05471200	-0.36916700

	Hartree	kcal/mol
E PBE0-D3BJ/def2TZVP	-885.26587	-555512.743
zpv	0.253919	159.336585
H	0.270049	169.458313
G	0.210805	132.28214
E PBE0-D3BJ/def2TZVP SMD (THF)	-885.289624	-555527.65
E TightPNO-DLPNO-CCSD(T)/def2-QZVPP	-884.599706	-555094.719

$3_{SH.AB}$

C	3.135570	-1.522802	0.295605
C	2.990420	-0.158932	0.438920
C	2.067634	-2.276765	-0.174444
C	1.769674	0.422848	0.089489
C	0.900297	-1.611787	-0.494303
H	2.136479	-3.350616	-0.287655
H	4.072854	-1.998752	0.560379
H	3.794649	0.455810	0.823533

N	0.752917	-0.292702	-0.380878
H	-1.576023	2.807172	-0.414358
H	-2.441030	-0.116110	-1.231358
H	-0.861823	0.362963	-1.067386
H	-1.850112	1.057751	-2.224195
H	0.255587	2.200311	0.105199
H	-3.429344	2.044693	-0.344693
H	-1.985577	1.349749	0.895415
N	-1.820767	0.697366	-1.277044
B	-2.242901	1.811696	-0.207808
S	1.594130	2.157019	0.276469
H	-3.513355	0.347162	0.975683
N	-4.061803	-0.510971	1.115293
H	-3.874772	-0.844976	2.055008
H	-5.043682	-0.260117	1.065271
H	-3.983285	-1.169295	-1.067700
H	-2.497117	-1.847267	0.132189
H	-4.354653	-2.621717	0.267728
B	-3.690907	-1.641042	0.019445
H	0.031179	-2.153498	-0.856439

	Hatree	kcal/mol
E PBE0-D3BJ/def2TZVP	-812.4813926	-509839.792431
zpv	0.232352	145.803087
H	0.248791	156.118716
G	0.187413	117.603438
E PBE0-D3BJ/def2TZVP SMD THF	-812.505673	-509855.028616
E TightPNO-DLPNO-CCSD(T)/def2-QZVPP	-811.882327	-509463.873077

$TS_{3SH.AB/6+H2}$

C	0.454199	-2.462277	-0.248825
C	1.332371	-1.411084	-0.393476
C	-0.901569	-2.208789	-0.062204
C	0.839384	-0.100683	-0.352704
C	-1.308314	-0.893077	-0.035216
H	-1.619512	-3.008830	0.062082
H	0.828368	-3.479552	-0.270800
H	2.393797	-1.588463	-0.516525
N	-0.469947	0.134193	-0.179254
H	0.444704	2.597473	1.409620
H	-1.827804	3.117036	-0.712242
H	-0.887930	1.712507	-0.383435
H	-0.295219	2.965130	-1.309801
H	0.967915	2.314367	0.673687
H	0.310214	4.450923	0.581605
H	-1.166383	3.516151	1.651106
N	-0.891249	2.787767	-0.505395
B	-0.325389	3.458372	0.792989
S	1.915393	1.265707	-0.536695

H	5.074282	0.280497	1.453404
N	4.857729	-0.021833	0.509575
H	3.937050	0.364854	0.259562
H	5.539121	0.400923	-0.112173
H	6.040268	-1.971058	0.660779
H	4.094232	-2.058829	1.200157
H	4.600529	-1.905130	-0.752297
B	4.902936	-1.640304	0.396041
H	-2.353840	-0.635739	0.108233

	Hatree	kcal/mol
E PBE0-D3BJ/def2TZVP	-812.4454942	-509817.217696
zpv	0.228792	143.569140
H	0.244525	153.441746
G	0.185569	116.446299
E SMD-PBE0-D3BJ/def2TZVP	-812.4721732	-509834.0072
E TightPNO-DLPNO-CCSD(T)/def2-QZVPP	-811.8386621	-509436.473

6

C	0.250851	2.760086	0.007838
C	-1.037181	2.274840	0.022913
C	1.317168	1.865949	-0.001000
C	-1.247800	0.889220	0.036885
C	1.022717	0.518526	0.016839
H	2.344660	2.205085	-0.019532
H	0.428841	3.829605	-0.008258
H	-1.891926	2.939152	0.009932
N	-0.217930	0.033856	0.044914
S	-2.888573	0.287744	0.082402
H	-2.225852	-1.500614	-1.693740
H	-3.823047	-1.989284	-0.538971
H	-1.485428	-3.191618	0.038400
H	-0.865872	-1.646776	0.357279
H	-2.057034	-2.323215	1.325541
B	-2.727249	-1.489554	-0.596243
N	-1.709483	-2.260700	0.373929
H	1.819271	-0.220148	0.015348

	Hatree	kcal/mol
E PBE0-D3BJ/def2TZVP	-728.1609638	-456927.922338
zpv	0.14386	90.273517
H	0.153423	96.274390
G	0.110127	69.105739
E PBE0-D3BJ/def2TZVP SMD THF	-728.179055	-456939.274801
E TightPNO-DLPNO-CCSD(T)/def2-QZVPP	-727.608324	-456581.135737

$TS_{6/3+NH2BH2}$

C	2.724795	-0.461637	0.005849
C	1.643924	-1.266072	0.218607
C	2.568340	0.923862	-0.216693
C	0.325438	-0.732764	0.222666
C	1.303013	1.423286	-0.210675
H	3.414517	1.573541	-0.386635
H	3.720151	-0.891944	0.007449
H	1.750161	-2.328471	0.390216
N	0.250710	0.614387	0.000830
S	-1.083618	-1.593625	0.468189
H	-3.389332	0.114653	1.409527
H	-3.538383	-0.233317	-0.647521
H	-2.440976	2.350622	0.752463
H	-0.711433	0.977800	0.008100
H	-2.559878	2.071105	-0.898193
B	-3.122029	0.391955	0.279889
N	-2.525171	1.662854	0.021715
H	1.072488	2.469172	-0.370641

	Hatree	kcal/mol
E PBE0-D3BJ/def2TZVP	-728.1349516	-456911.599441
zpv	0.141956	89.078739
H	0.151877	95.304260
G	0.106554	66.863647
E PBE0-D3BJ/def2TZVP SMD THF	-728.153282	-456923.101786
E TightPNO-DLPNO-CCSD(T)/def2-QZVPP	-727.582683	-456565.045903

$4_{SH.AB}$

C	0.425313	-2.548910	-0.401264
C	1.307183	-1.507089	-0.600133
C	-0.905295	-2.277511	-0.122975
C	0.794826	-0.217186	-0.523333
C	-1.333303	-0.958011	-0.061137
H	-1.598030	-3.087992	0.050104
H	0.780752	-3.572114	-0.441247
H	2.362966	-1.680162	-0.769296
N	-0.480414	0.052445	-0.278758
H	0.728067	3.267179	0.956966
H	-1.950555	3.231493	-0.692240
H	-0.929451	1.934623	-0.462004
H	-0.459517	3.234919	-1.368571
H	1.339973	2.021692	0.042170
H	-0.405213	4.879574	0.577256
H	-1.133834	3.357383	1.700962
N	-0.983074	2.967204	-0.540793
B	-0.410180	3.686725	0.787158
S	1.914520	1.128255	-0.811877
C	-2.773590	-0.570923	0.238777

C	-2.798905	0.366242	1.448738
H	-2.183474	1.253830	1.303332
H	-3.824211	0.687788	1.648370
H	-2.426648	-0.145378	2.339647
C	-3.359591	0.131358	-0.989693
H	-3.392622	-0.545498	-1.846959
H	-4.378587	0.465819	-0.779847
H	-2.766648	0.999109	-1.278244
C	-3.640358	-1.786383	0.551087
H	-3.699351	-2.476446	-0.294070
H	-3.271135	-2.333416	1.421718
H	-4.656419	-1.455355	0.776069
H	4.693121	-0.002844	1.898705
N	4.674158	-0.094122	0.888413
H	3.806720	0.327055	0.545331
H	5.443320	0.452081	0.514331
H	5.902716	-2.018155	0.779361
H	3.907624	-2.239948	1.012687
H	4.661481	-1.656382	-0.772641
B	4.799498	-1.651736	0.436109

	Hatree	kcal/mol
E PBE0-D3BJ/def2TZVP	-969.5999683	-412.3837869
zpv	0.344007	0.146310761
H	0.366356	0.155816089
G	0.292431	0.124374801
E PBE0-D3BJ/def2TZVP SMD THF	-969.631455	-412.3971786
E TightPNO-DLPNO-CCSD(T)/def2-QZVPP	-968.8849441	-412.0796776

$TS_{4SH.AB/5+H2}$

C	0.473240	-2.462924	-0.257293
C	1.329518	-1.399153	-0.414804
C	-0.884568	-2.238061	-0.057925
C	0.797556	-0.106288	-0.371882
C	-1.350853	-0.936012	-0.030650
H	-1.559258	-3.070360	0.077679
H	0.862825	-3.474627	-0.276077
H	2.393598	-1.550755	-0.545223
N	-0.513263	0.100087	-0.192360
H	0.430471	2.581500	1.444521
H	-1.790999	3.156323	-0.729547
H	-0.902179	1.727402	-0.386355
H	-0.236657	2.966903	-1.267291
H	0.948460	2.297407	0.699982
H	0.283987	4.449478	0.658990
H	-1.203491	3.473482	1.675090
N	-0.872127	2.799165	-0.490739
B	-0.342038	3.444088	0.835768

S	1.858975	1.277062	-0.561877
C	-2.819466	-0.588191	0.170380
C	-2.959016	0.352113	1.369987
H	-2.381368	1.268126	1.246932
H	-4.007679	0.626464	1.510622
H	-2.610108	-0.133827	2.284170
C	-3.346820	0.091648	-1.096581
H	-3.288351	-0.584022	-1.953050
H	-4.391738	0.382707	-0.961634
H	-2.773590	0.985371	-1.345029
C	-3.669733	-1.826864	0.433231
H	-3.648533	-2.522136	-0.408998
H	-3.342013	-2.358377	1.329511
H	-4.708726	-1.526768	0.586595
H	5.019361	0.357943	1.444432
N	4.816240	0.041161	0.502373
H	3.887737	0.402829	0.243355
H	5.490459	0.475255	-0.119366
H	6.048768	-1.874320	0.679236
H	4.102467	-2.008604	1.208901
H	4.615580	-1.860548	-0.742174
B	4.904273	-1.576748	0.405076

	Hatree	kcal/mol
E PBE0-D3BJ/def2TZVP	-969.5766169	-608418.5381
zpv	0.341086	214.0347053
H	0.362349	227.3774398
G	0.291785	183.0978595
E PBE0-D3BJ/def2TZVP SMD THF	-969.6051606	-608436.4495
E TightPNO-DLPNO-CCSD(T)/def2-QZVPP	-968.8541018	-607965.153

AB

H	1.674092	-1.067745	1.779029
N	1.206938	-0.890402	2.662411
H	1.690860	-1.416213	3.383063
H	1.306327	0.096876	2.875760
B	-0.372380	-1.316243	2.584932
H	-0.825700	-1.065340	3.680961
H	-0.844038	-0.633473	1.701482
H	-0.372145	-2.501023	2.327492

	Hatree	kcal/mol
E PBE0-D3BJ/def2TZVP	-83.1381945	-52170.00686
zpv	0.069998	43.92440998
H	0.074734	46.89629497
G	0.046558	29.2155873
E PBE0-D3BJ/def2TZVP SMD THF	-83.1569567	-52181.78032
E TightPNO-DLPNO-CCSD(T)/def2-QZVPP	-83.089520	-52139.463348

AB_2

H	1.186548	1.080333	1.014342
H	1.141318	2.815724	0.000000
H	1.186548	1.080333	-1.014342
H	-1.177808	2.036311	-0.819977
H	-1.119830	0.605472	0.000000
H	-1.177808	2.036311	0.819977
H	-1.186548	-1.080333	-1.014342
H	-1.186548	-1.080333	1.014342
H	-1.141318	-2.815724	0.000000
H	1.177808	-2.036311	-0.819977
H	1.119830	-0.605472	0.000000
H	1.177808	-2.036311	0.819977
B	-0.822005	-1.649789	0.000000
N	0.792654	-1.579194	0.000000
B	0.822005	1.649789	0.000000
N	-0.792654	1.579194	0.000000

	Hatree	kcal/mol
E PBE0-D3BJ/def2TZVP	-166.3032082	-104356.8431
zpv	0.142578	89.46904949
H	0.151714	95.20197628
G	0.112111	70.35071755
E PBE0-D3BJ/def2TZVP SMD THF	-166.3238343	-104369.7861
E TightPNO-DLPNO-CCSD(T)/def2-QZVPP	-166.203335	-104294.171481

$TS'_{4SH.AB/5+H2}$

C	2.742766	0.142278	0.018213
C	1.743902	1.072285	-0.062485
C	2.441981	-1.224181	0.064168
C	0.401072	0.653593	-0.105749
C	1.131560	-1.626339	0.000020
H	3.231429	-1.954557	0.148353
H	3.777592	0.463731	0.059753
H	1.946847	2.134001	-0.076976
N	0.165304	-0.682739	-0.092996
H	-1.931918	0.701812	0.782107
H	-2.688204	-0.568456	-1.387723
H	-2.809202	-2.058261	-0.734439
H	-0.868216	-0.966450	-0.260309
H	-2.256963	0.114443	1.429162
H	-4.047791	0.269880	0.525217
H	-3.379161	-1.375152	1.537180
N	-2.500122	-1.112443	-0.554873
B	-3.169192	-0.540941	0.684428
S	-0.894772	1.753684	-0.154300

C	0.682668	-3.073907	0.015682
C	-0.332766	-3.281472	1.144909
H	-1.220100	-2.659010	1.031395
H	-0.653573	-4.325687	1.157942
H	0.116294	-3.053849	2.114497
C	0.053289	-3.412363	-1.341196
H	0.784957	-3.301953	-2.144917
H	-0.293372	-4.448517	-1.336045
H	-0.797912	-2.769692	-1.564510
C	1.860005	-4.015033	0.247453
H	2.600811	-3.948531	-0.552498
H	2.355334	-3.817580	1.200835
H	1.492866	-5.042858	0.270502

	Hatree	kcal/mol
E PBE0-D3BJ/def2TZVP	-886.4199545	-556236.9424
zpv	0.268736	168.634393
H	0.284547	178.5559457
G	0.22812	143.1474671
E PBE0-D3BJ/def2TZVP SMD THF	-886.4361577	-556247.1101
E TightPNO-DLPNO-CCSD(T)/def2-QZVPP	-885.746084	-555814.082041

$TS_{BH3NH2BHNH2}$

H	-2.327035	-0.804359	-0.250496
H	-0.119299	1.226672	0.749013
H	-0.606312	-0.545129	1.673032
H	1.625868	-1.263237	0.534835
H	0.799488	-1.205151	-0.931540
H	2.008401	1.257087	0.644796
H	0.987230	1.325967	-1.167615
H	-1.871547	0.637076	-0.991352
N	1.203494	-0.686167	-0.171481
N	-1.704997	-0.016572	-0.247389
B	1.319269	0.735246	-0.181070
B	-0.676048	0.139397	0.696913

	Hatree	kcal/mol
E PBE0-D3BJ/def2TZVP	-163.9250217	-102864.5084
E+zpv	0.098552	61.84231624
H	0.105342	66.10310575
G	0.07069	44.35864656
E PBE0-D3BJ/def2TZVP SMD THF	-163.929464	-102867.296
E TightPNO-DLPNO-CCSD(T)/def2-QZVPP	-163.819324	-102798.182395

$BH_3NH_2BHNH_2$

H	-0.017021	-2.209282	0.632377

H	0.912906	1.396015	1.202688
H	2.063409	-1.030129	1.510716
H	3.065726	0.470715	-0.178024
H	2.031299	0.100381	-1.399591
H	1.801310	2.546741	-0.201610
H	0.153998	1.440468	-0.661154
H	-0.096969	-1.302494	-0.783109
N	2.103555	0.252202	-0.401283
N	0.436530	-1.539947	0.036805
B	1.151663	1.550547	0.018301
B	1.530772	-0.808951	0.469093

	Hatree	kcal/mol
E PBE0-D3BJ/def2TZVP	-163.948292	-102879.1107
E+zpv	0.100501	63.06533226
H	0.107509	67.46291884
G	0.072186	45.29740077
E PBE0-D3BJ/def2TZVP SMD THF	-163.9633151	-102888.5379
E TightPNO-DLPNO-CCSD(T)/def2-QZVPP	-163.843776	-102813.525873

TS_{NH2BH2_B2N2H8}

H	-0.287076	-2.124411	0.155508
H	-2.416163	-1.125513	0.938676
H	-2.491291	-0.930741	-1.133952
H	-2.213513	1.565132	-0.700985
H	-2.174615	1.402213	0.969934
H	0.366250	0.934224	0.295998
H	0.732259	-0.398490	1.737117
H	2.980765	-0.074072	0.717620
H	2.538762	-0.677594	-0.767322
H	1.897905	2.160641	0.311346
H	1.233034	1.322155	-1.458917
H	-0.008582	-1.255236	-1.227644
N	2.252055	-0.119823	0.022457
N	-0.107997	-1.208609	-0.224514
N	-2.215277	0.935939	0.081561
B	1.502742	1.209905	-0.297264
B	0.798271	-0.345749	0.543577
B	-2.238703	-0.469646	-0.052584

	Hatree	kcal/mol
E PBE0-D3BJ/def2TZVP	-245.9159544	-154314.5976
E+zpv	0.152066	95.42285963
H	0.161658	101.4419308
G	0.118981	74.66170782
E PBE0-D3BJ/def2TZVP SMD THF	-245.9276247	-154321.9208
E TightPNO-DLPNO-CCSD(T)/def2-QZVPP	-245.755133	-154213.680499

$NH_2BH_2\_B_2N_2H_8$

H	-0.372248	-1.926183	-0.468472
H	-2.081785	-1.204464	1.098820
H	-2.659510	-0.942262	-0.836053
H	-2.599047	1.478827	-0.321694
H	-2.222575	1.244980	1.256733
H	0.428652	0.975741	0.438890
H	0.607407	-0.626868	1.617158
H	2.849175	-0.550963	0.551016
H	2.282663	-0.741810	-1.000488
H	2.172502	1.875849	0.667342
H	1.369628	1.552463	-1.217349
H	-0.331687	-0.575972	-1.395061
N	2.116075	-0.317519	-0.100680
N	-0.400292	-0.911935	-0.438562
N	-2.034050	0.941380	0.315327
B	1.626695	1.161161	-0.117077
B	0.668898	-0.337760	0.462152
B	-1.980610	-0.530920	0.090230

	Hatree	kcal/mol
E PBE0-D3BJ/def2TZVP	-245.9199197	-154317.0858
E+zpv	0.154694	97.07195459
H	0.163965	102.8895952
G	0.122663	76.9721978
E PBE0-D3BJ/def2TZVP SMD THF	-245.938746	-154328.8995
E TightPNO-DLPNO-CCSD(T)/def2-QZVPP	-245.759125	-154216.185881

$TS_{B3N3H12}$

H	0.645059	2.091268	-0.560805
H	2.166416	0.884830	1.039205
H	2.634652	0.558955	-0.908511
H	1.927564	-1.751546	-0.511521
H	1.613250	-1.511816	1.073944
H	-0.386820	-0.005291	1.345394
H	-1.091053	1.848918	1.096387
H	-2.791646	0.297186	0.381424
H	-2.151235	0.453703	-1.135573
H	-1.636600	-1.846880	0.923436
H	-0.794191	-1.648031	-0.981930
H	0.261176	0.752489	-1.393367
N	-1.973193	0.136550	-0.191269
N	0.449215	1.101838	-0.458858
N	1.493442	-1.126057	0.150143
B	-1.307780	-1.197791	-0.015192
B	-0.704084	0.854340	0.539390
B	1.852949	0.333131	0.001619

	Hatree	kcal/mol
E PBE0-D3BJ/def2TZVP	-245.8867465	-154296.2693
E+zpv	0.153043	96.03593641
H	0.161888	101.5862579
G	0.121833	76.45136491
E PBE0-D3BJ/def2TZVP SMD THF	-245.9054336	-154307.9957
E TightPNO-DLPNO-CCSD(T)/def2-QZVPP	-245.727212	-154196.159951

$B_3N_3H_{12}$

H	0.000000	2.394136	0.234126
H	1.431619	0.826546	1.445679
H	2.282892	1.318028	-0.325059
H	1.439708	-0.831216	-1.195534
H	2.073382	-1.197068	0.234126
H	-1.431619	0.826546	1.445679
H	-2.282892	1.318028	-0.325059
H	-2.073382	-1.197068	0.234126
H	-1.439708	-0.831216	-1.195534
H	0.000000	-1.653092	1.445679
H	0.000000	-2.636057	-0.325059
H	0.000000	1.662432	-1.195534
N	-1.276897	-0.737217	-0.196249
N	0.000000	1.474433	-0.196249
N	1.276897	-0.737217	-0.196249
B	0.000000	-1.561694	0.242897
B	-1.352467	0.780847	0.242897
B	1.352467	0.780847	0.242897

	Hatree	kcal/mol
E PBE0-D3BJ/def2TZVP	-245.9762362	-154352.425
zpv	0.159179	99.8863347
H	0.16691	104.7376106
G	0.130451	81.85924178
E PBE0-D3BJ/def2TZVP SMD THF	-245.9945952	-154363.9454
E TightPNO-DLPNO-CCSD(T)/def2-QZVPP	-245.817819	-154253.016715

4

N	0.394532	0.417387	0.000880
C	-0.718151	-0.357316	-0.000158
C	-0.551536	-1.710969	-0.001486
C	0.760392	-2.234810	-0.001676
C	1.851273	-1.420619	-0.000563
C	1.701663	-0.005617	0.000799
S	2.931158	1.111593	0.002237
H	2.858784	-1.811933	-0.000683
H	0.896624	-3.310890	-0.002728

H	-1.406489	-2.368639	-0.002392
C	-2.043173	0.376781	0.000482
C	-2.136336	1.255529	-1.252337
H	-1.352328	2.015284	-1.288902
H	-3.096612	1.775830	-1.265520
H	-2.060723	0.651666	-2.159050
C	-2.137041	1.251575	1.256035
H	-2.062146	0.644794	2.160855
H	-3.097224	1.772028	1.270239
H	-1.352838	2.010981	1.295496
C	-3.209684	-0.603636	-0.001426
H	-3.199894	-1.239637	-0.889112
H	-3.200669	-1.242186	0.884436
H	-4.148638	-0.046664	-0.001011
H	0.298417	1.423513	0.001819

	Hatree	kcal/mol
E PBE0-D3BJ/def2TZVP	-803.2958916	-504075.8033
zpv	0.204755	128.4857077
H	0.216797	136.0421771
G	0.168004	105.424106
E SMD-PBE0-D3BJ/def2TZVP	-803.316706	-504088.864300
E DLPNO-CCSD(T)/def2-TZVP	-802.680992	-503689.948100

3

N	-6.046927	-0.142792	-0.445313
C	-7.275871	-0.570081	0.004410
C	-7.922168	-1.490882	-0.872797
C	-7.345100	-1.888055	-2.040456
C	-6.075984	-1.399789	-2.429672
C	-5.457017	-0.523508	-1.598481
S	-7.874225	-0.013381	1.446948
H	-5.605514	-1.705467	-3.352633
H	-7.865581	-2.589638	-2.682960
H	-8.891608	-1.857504	-0.565324
H	-5.582084	0.510018	0.170469
H	-4.486182	-0.090631	-1.801229

	Hatree	kcal/mol
E PBE0-D3BJ/def2TZVP	-646.1631408	-405473.5094
E+zpv	0.092417	57.99254546
H	0.098901	62.06131706
G	0.062579	39.268917
E SMD-PBE0-D3BJ/def2TZVP	-646.182233	-405485.489700
E DLPNO-CCSD(T)/def2-TZVP	-645.664403	-405160.546800

NH_2BH_2

N	0.000000	0.000000	0.609560
H	0.841345	-0.000048	1.160809
B	0.000000	0.000000	-0.775208
H	-0.841345	0.000048	1.160809
H	1.046785	0.000039	-1.356250
H	-1.046785	-0.000039	-1.356250

	Hatree	kcal/mol
E PBE0-D3BJ/def2TZVP	-81.96475329	-51433.66135
E+zpv	0.048053	30.153714
H	0.052223	32.77042862
G	0.026313	16.511657
E PBE0-D3BJ/def2TZVP SMD THF	-81.967242	-51435.223060
E TightPNO-DLPNO-CCSD(T)/def2-QZVPP	-81.914922	-51402.391450

18 References

[1] V. Miranda-Soto, J. J. Pérez-Torrente, L. A. Oro, F. J. Lahoz, M. L. Martín, M. Parra-Hake, D. B. Grotjahn, *Organometallics* **2006**, *25*, 4374-4390.

[2] L. Hintermann, T. T. Dang, A. Labonne, T. Kribber, L. Xiao, P. Naumov, *Chemistry – A European Journal* **2009**, *15*, 7167-7179.

[3] T. M. Maier, S. Sandl, I. G. Shenderovich, A. Jacobi von Wangelin, J. J. Weigand, R. Wolf, *Chemistry – A European Journal* **2019**, *25*, 238-245.

[4] Y.-Q. Zou, N. von Wolff, A. Anaby, Y. Xie, D. Milstein, *Nature Catalysis* **2019**, *2*, 415-422.

[5] a) M. Roselló-Merino, J. López-Serrano, S. Conejero, *Journal of the American Chemical Society* **2013**, *135*, 10910-10913; b) Z. Mo, A. Rit, J. Campos, E. L. Kolychev, S. Aldridge, *Journal of the American Chemical Society* **2016**, *138*, 3306-3309; c) L. Luconi, E. S. Osipova, G. Giambastiani, M. Peruzzini, A. Rossin, N. V. Belkova, O. A. Filippov, E. M. Titova, A. A. Pavlov, E. S. Shubina, *Organometallics* **2018**, *37*, 3142-3153.

[6] L. Krause, R. Herbst-Irmer, G. M. Sheldrick, D. Stalke, *Journal of Applied Crystallography* **2015**, *48*, 3-10.

[7] G. Sheldrick, *Acta Crystallographica Section A* **2015**, *71*, 3-8.

[8] G. Sheldrick, *Acta Crystallographica Section C* **2015**, *71*, 3-8.

[9] A. Spek, *Acta Crystallographica Section C* **2015**, *71*, 9-18.

[10] A. Spek, *Acta Crystallographica Section D* **2009**, *65*, 148-155.

[11] Ł. Szyc, J. Guo, M. Yang, J. Dreyer, P. M. Tolstoy, E. T. J. Nibbering, B. Czarnik-Matusewicz, T. Elsaesser, H.-H. Limbach, *The Journal of Physical Chemistry A* **2010**, *114*, 7749-7760.

[12] D. Moran, K. Sukcharoenphon, R. Puchta, H. F. Schaefer, P. v. R. Schleyer, C. D. Hoff, *The Journal of Organic Chemistry* **2002**, *67*, 9061-9069.

[13] a) J. P. Perdew, K. Burke, M. Ernzerhof, *Physical Review Letters* **1996**, *77*, 3865-3868; b) C. Adamo, V. Barone, *The Journal of Chemical Physics* **1999**, *110*, 6158-6170.

[14] S. Grimme, S. Ehrlich, L. Goerigk, *Journal of Computational Chemistry* **2011**, *32*, 1456-1465.

[15] A. V. Marenich, C. J. Cramer, D. G. Truhlar, *The Journal of Physical Chemistry B* **2009**, *113*, 6378-6396.

[16] C. Riplinger, B. Sandhoefer, A. Hansen, F. Neese, *The Journal of Chemical Physics* **2013**, *139*, 134101.

[17] a) F. Neese, *Wiley Interdisciplinary Reviews: Computational Molecular Science* **2018**, *8*, e1327; b) G. W. T. M. J. Frisch, H. B. Schlegel, G. E. Scuseria, M. A. Robb, J. R. Cheeseman, G. Scalmani, V. Barone, G. A. Petersson, H. Nakatsuji, X. Li, M. Caricato, A. V. Marenich, J. Bloino, B. G. Janesko, R. Gomperts, B. Mennucci, H. P. Hratchian, J. V. Ortiz, A. F. Izmaylov, J. L. Sonnenberg, D. Williams-Young, F. Ding, F. Lipparini, F. Egidi, J. Goings, B. Peng, A. Petrone, T. Henderson, D. Ranasinghe, V. G. Zakrzewski, J. Gao, N. Rega, G. Zheng, W. Liang, M. Hada, M. Ehara, K. Toyota, R. Fukuda, J. Hasegawa, M. Ishida, T. Nakajima, Y. Honda, O. Kitao, H. Nakai, T. Vreven, K. Throssell, J. A. Montgomery, Jr., J. E. Peralta, F. Ogliaro, M. J. Bearpark, J. J. Heyd, E. N. Brothers, K. N. Kudin, V. N. Staroverov, T. A. Keith, R. Kobayashi, J. Normand, K. Raghavachari, A. P. Rendell, J. C. Burant, S. S. Iyengar, J. Tomasi, M. Cossi, J. M. Millam, M. Klene, C. Adamo, R. Cammi, J. W. Ochterski, R. L. Martin, K. Morokuma, O. Farkas, J. B. Foresman, and D. J. Fox, Gaussian, Inc., Wallingford CT, 2016., **2017**.

[18] F. Weigend, R. Ahlrichs, *Phys Chem Chem Phys* **2005**, *7*, 3297-3305.
